# Signatures of positive selection in Toll-like receptor (TLR) genes in mammals

**DOI:** 10.1186/1471-2148-11-368

**Published:** 2011-12-20

**Authors:** Helena Areal, Joana Abrantes, Pedro J Esteves

**Affiliations:** 1 CIBIO-UP, Centro de Investigacao em Biodiversidade e Recursos Geneticos - Universidade do Porto, Campus Agrario de Vairao, Rua Padre Armando Quintas, nr.7, 4485-661 Vairao, Portugal; 2 Departamento de Zoologia e Antropologia da Faculdade de Ciencias, Universidade do Porto, Porto, Portugal; 3 INSERM, U892, Universite de Nantes, 44007 Nantes, France; 4 CITS, Centro de Investigacao em Tecnologias de Saude, CESPU, Gandra, Portugal

**Keywords:** Toll-like receptors, PAMPs, host-pathogen interaction, adaptive evolution, positive selection, viral TLRs, non-viral TLRs

## Abstract

**Background:**

Toll-like receptors (TLRs) are a major class of pattern recognition receptors (PRRs) expressed in the cell surface or membrane compartments of immune and non-immune cells. TLRs are encoded by a multigene family and represent the first line of defense against pathogens by detecting foreigner microbial molecular motifs, the pathogen-associated molecular patterns (PAMPs). TLRs are also important by triggering the adaptive immunity in vertebrates. They are characterized by the presence of leucine-rich repeats (LRRs) in the ectodomain, which are associated with the PAMPs recognition. The direct recognition of different pathogens by TLRs might result in different evolutionary adaptations important to understand the dynamics of the host-pathogen interplay. Ten mammal TLR genes, viral (TLR3, 7, 8, 9) and non-viral (TLR1-6, 10), were selected to identify signatures of positive selection that might have been imposed by interacting pathogens and to clarify if viral and non-viral TLRs might display different patterns of molecular evolution.

**Results:**

By using Maximum Likelihood approaches, evidence of positive selection was found in all the TLRs studied. The number of positively selected codons (PSC) ranged between 2-26 codons (0.25%-2.65%) with the non-viral TLR4 as the receptor with higher percentage of positively selected codons (2.65%), followed by the viral TLR8 (2.50%). The results indicated that viral and non-viral TLRs are similarly under positive selection. Almost all TLRs have at least one PSC located in the LRR ectodomain which underlies the importance of the pathogen recognition by this region.

**Conclusions:**

Our results are not in line with previous studies on primates and birds that identified more codons under positive selection in non-viral TLRs. This might be explained by the fact that both primates and birds are homogeneous groups probably being affected by only a restricted number of related viruses with equivalent motifs to be recognized. The analyses performed in this work encompassed a large number of species covering some of the most representative mammalian groups - Artiodactyla, Rodents, Carnivores, Lagomorphs and Primates - that are affected by different families of viruses. This might explain the role of adaptive evolution in shaping viral TLR genes.

## Background

Toll-like receptors (TLRs) are a major class of pattern recognition receptors (PRRs) in *Drosophila *and in mammals. Mammalian TLRs have essential roles in recognizing infectious agents and initiating intracellular signal transduction pathways that trigger the expression of genes leading to both innate and adaptive immune responses [1,2]. TLRs belong to the type I transmembrane glycoprotein receptor family and can be expressed either in the cell surface or membrane compartments of immune and non-immune cells (e.g. epithelial cells) [3,4].

Thirteen TLRs have been identified to date of which 10 members are present in the human genome (TLR1-10) and thirteen in rodents (TLR1-13) [5]. According to their structure, phylogenetic position and class of microbial compound that they recognize, vertebrate TLRs can be classified into six subfamilies: the TLR1 subfamily encompasses TLR1, 2, 6 and 10; the TLR9 subfamily includes TLR7, 8 and 9; TLR11, 12 and 13 constitute the subfamily TLR11 and TLR3, TLR4 and TLR5 each form a subfamily [6,7]. TLRs can be also classified into non-viral (TLR1, 2, 4, 5, 6, 10) and viral TLRs (TLR3, 7, 8, 9) with respect to their ligand recognition [8-10].

The TLR family is structurally characterized by the presence of an ectodomain, a signal transmembrane segment and a highly conserved cytoplasmic domain homologous to the human interleukin-1 receptor (IL1R) and human IL-18 receptor (IL-18R) and designated TIR domain [11,12]. Crystallographic studies of the ectodomain revealed a solenoid horseshoe-like structure constituted by a high but variable number (16-28) of leucine-rich repeats (LRRs) responsible for binding the "pathogen associated molecular patterns" (PAMPs). PAMPs are present in pathogens and not in host components, thus allowing the innate immune system to distinguish between what is self and what is non-self [13]. Nevertheless, TLRs not only sense microbial components but they can also target endogenous molecules that have resulted from host dying cells and which can activate the inflammatory response [14,15]. PAMPs are characteristic molecular signatures of the pathogens, highly conserved during evolution since they are involved in critical functions and are essential for survival of the pathogens. Indeed, mutations or loss of these patterns can be lethal to the pathogens and therefore are quite conserved [13]. This means that a limited number of PRRs is needed to detect the presence of an infection [13]. Their main recognition molecules, the LRRs, are capped in the amino and carboxy termini by LRR-NT and LRR-CT molecules, respectively [16,17], that stabilize the protein structure by protecting the hydrophobic core from exposure to solvent [18]. Delimitation of these domains is, however, not consensual among the different software programs used for their determination [19,20]. For some TLRs crystallographic models have been made available where these domains are well defined (e.g. the TLR1/TLR2, TLR2/TLR6 heterodimers, TLR3 and TLR4 of *Homo sapiens *and of *Mus musculus *[16,17,21-23]). However, this is not the case for all species which further complicates the correct delimitation of each domain.

Upon PAMPs recognition, TLRs dimerize leading to the recruitment of the adaptor proteins (e.g. MyD88, TRIF) and triggering signaling pathways of the innate immune response (reviewed in [24]). TLR1/TLR2 and TLR2/TLR6 exist as heterodimers on the cell surface and detect bacterial triacylated and diacylated lipoproteins, respectively [25]. Also on the cell surface, TLR4 and TLR5 detect lipopolysaccharide (LPS) and bacterial flagellin, correspondingly [25]. TLR10 seems to heterodimerize with TLR2 to also recognize triacyl lipoproteins [26,27]. On the other hand, TLR3, 7, 8 and 9 are expressed on endosomes and detect microbial nucleic acids [4,10,28] with TLR3 being associated with the recognition of double-strand RNA (dsRNA) [29], while TLR7 and 8 target viral components such as single-strand RNA (ssRNA) and TLR9 respond to double-strand DNA viruses (dsDNA) and bacteria by recognizing non-methylated CpG-containing DNA [10,30].

TLRs are evolutionary conserved proteins and their characterization and of their ligands has contributed to the understanding of the function of the TLRs and to the host defense processes against infections [31]. They are candidate molecules to examine how natural selection molds innate immunity receptors. Several studies have been performed and purifying selection was suggested as the major force driving TLRs evolution, at least in humans [32,33]. However, other studies on Primate species revealed different degrees of positive selection acting on their evolutionary history. Evidence of positive selection was found in TLR1 [34] and TLR4 [35]. In a broader study in a primate group, Wlasiuk and Nachman (2010) showed evidence of positive selection in six TLR genes, TLR1, TLR4, TLR6, TLR7, TLR8 and TLR9, with the non-viral TLR4 having the highest number of positively selected codons (PSC). A study performed by Alcaide and Edwards (2011) in birds showed evidence of positive selection in TLR4. The most recent analysis of the TLR1 subfamily showed evidence of positive selection in TLR1, 2 and 6 in mammals and TLR2A/B in birds [36]. Overall, these studies also showed that non-viral TLRs tend to be more prone to positive selection than viral TLRs. From an evolutionary point of view, proteins involved in direct recognition of pathogens might have been shaped by these interactions. Here, we have studied ten mammal TLR genes in order to look for evidence of positive selection and to further clarify if viral and non-viral TLRs display different patterns of molecular evolution due to the different nature of the PAMPs they recognize.

## Results and Discussion

### Signatures of positive selection

Genes of the immune system, in particular those involved in the recognition of pathogens, and genes involved in the host-pathogen interaction have been shown to be highly prone to adaptive selection (e.g. [37,38]). By using Maximum-Likelihood (ML) approaches, evidence of positive selection was detected in all the TLRs studied (Table 1). For seven of the TLRs (TLR1-6, TLR10), analyses included species belonging to some of the most representative mammalian groups, i.e. Artiodactyla, Rodents, Carnivores, Lagomorphs and Primates, while for the remaining three TLRs, the Lagomorph group was not included due to the lack of data (Additional file 1, Table S1; Additional file 2, Table S2; Additional file 3, Table S3; Additional file 4, Table S4; Additional file 5, Table S5; Additional file 6, Table S6; Additional file 7, Table S7; Additional file 8, Table S8; Additional file 9, Table S9; Additional file 10, Table S10).

**Table 1:** Phylogenetic Tests of Positive Selection

**Sites under positive selection identified by different methods **^**a**^										
**Gene**	**No. of species**	**lnL M7**	**lnL M8**	**-2lnΔL**	**PAML M8**	**SLAC**^**b**^	**FEL**^**c**^	**REL**^**d**^	**Total no. of sites**	**%. of sites**

**TLR1**	17	-14643.639	-14633.663	20.00	**174**, 318	174, 293	4,15,27,46, 61, 186, 231, 275, 289, 293, 317, 339, 352, 366, 378, 386, 417, 447, 458, 463, 477, 536, 539, 555, 590, 597, 599, 602, 606	-	2	0.25

**TLR2**	23	-21078.575	-21069.646	17.86	296, 453	3, 161, 182, 302, 602, 636	3, 63, 93, 111, 133, 161, 182, 215, 220, 261, 266, 302, 303, 321, 602, 636, 770	302, 602, 636	6	0.77

**TLR3**	20	-18074.201	-18060.466	27.47	4, 269, 506, 749	4,12, 712	4, 7, 11, 12, 25, 69, **79**, 139, 167, 258, 285, 326, 406, 432, 456, 616, 693, 712, 715, 741, 780, 782, 854	4, 12, 25, 79, 258, 285, 473, 558, 588, 712, 749, 780	9	0.99

**TLR4**	19	-20735.203	-20658.940	152.53	5, 271, 276, 295, 298, 321, 322, 325, 349, 351, 364, 368, 370, 371, 394, 400, 437, 460, 468, 471, 505, 520, 542	204, 300, 301, 317, 363, 382, 394, 468, 471, 487, 542, 604, 673	9, 56, 58, 120, 189, 193, 203, **204**, 240, 250, 270, 276, 290, 295, **300**, 301, 317, **319**, 323, 324, 329, 336, 342, 356, 357, 363, 370, 382, 394, 396, 447, 468, **471**,487, 493, 500, **542**, 604, 622, **639**, 673, 822	4, 161, 204, 240, 270, 276, 295, 300, 301, 317, 319, 329, 338, 356, 360, 363, 370, 447, 468, 471, 474, 487, 493, 500, 537, 542, 613, 616, 639, 648	22	2.65

**TLR5**	17	-23742.766	-23737.519	10.49	305, 466, 592	674, 742	14, 71, 128, 154, 170, 207, 340, 382, 400, 408, 420, 674, 721, 742	14, 128, 207, 400, 721	7	0.81

**TLR6**	20	-17602.606	-17594.945	15.32	293, 471	604, 607, 796	2, 20, 32, 56, 72, 89, 149, 219, 315, 412, 428, 463, 482, 501, 541, 544, 595, 602, 604, 607, 611, 626, 740, 743, 796	-	3	0.38

**TLR7**	21	-15564.048	-15551.690	24.72	697	359, 667	19, 89, 103, 113, 125, 151, 162, 283, 357, 359, 386, 398, 413, 425, 487, 496, 530, 566, 599, 667, 697, 719, 776, 885, 1017	39, 111, 283, 359, 386, 388, 528, 599, 667, 693, 697, 737, 776	7	0.67

**TLR8**	18	-23527.219	-23483.262	87.91	1, 100, 109, 110, 138, 146, 160, 174, 186, 188, 191, 214, 235, 249, 268, 288, 338, 349, 361, 367, 392, 416, 441, 459, 472, 481, 498, 603, 608, 637, 639, 675, 691, 699, 739, 761, 770	174, 388, 418, 459, 472, 712, 766	22, 24, 39, 101, 160, 214, 236, 238, 249, 290, 331, 388, 413, 416, 418, 441, 442, 459, 472, 481, 498, 508, 593, 606, 629, 633, 677, 712, 766, 826, 833, 840	5, 39, 102, 146, 174, 191, 214, 236, 246, 249, 285, 312, 338, 349, 350, 371, 388, 413, 416, 418,441, 451, 470, 472, 481, 498, 606, 629, 633, 639, 761, 766	26	2.50

**TLR9**	21	-18708.518	-18689.712	37.61	91, 185, 217, 302, 355, 449, 674	71, 161, 217, 332, 702	8, 42, 71, 161, 332, 625	71, 161, 397, 728	4	0.39

**TLR10**	13	-11931.949	-11928.377	7.14	-	91, 392, 469, 545	34, 71, 76, 89, 91, 238, 246, 273, 369, 392, 395, 396, 423, 469, 492, 545, 552, 600, 679, 775, 799, 803	110, 235, 238, 261, 345, 392, 400, 492, 803	7	0.86

^a ^Codons identified by more than one ML method are underlined. The identified codons that are in agreement with Wlasiuk and Nachman (2010) are in bold.

^b, c ^Codons with P values <0.1

^d ^Codons with Bayes factor >50

The number of positively selected codons observed for each TLR studied ranged between 2-26 which corresponded to 0.25%-2.65% of codons under positive selection. The non-viral TLR4 was the receptor with the highest proportion of PSC (22 positions, 2.65%), followed by the viral TLR8 (26 positions, 2.50%). Description of the amino acids present in each species for each site under positive selection and their location in the receptors can be found in Additional file 11, Table S11; Additional file 12, Table S12; Additional file 13, Table S13; Additional file 14, Table S14; Additional file 15, Table S15; Additional file 16, Table S16; Additional file 17, Table S17; Additional file 18, Table S18; Additional file 19, Table S19; Additional file 20, Table S20. Previous studies argued that viral TLRs are under a stronger purifying selection than non-viral TLRs [33,39,40] since viral TLRs recognize viral nucleic acids but also target self components [1,2,41]. Therefore, these TLRs have the dual role of maintaining their function and avoid autoimmunity, and so they are not expected to accumulate non-synonymous substitutions as this might affect their functional integrity. On the other hand, non-viral TLRs that exist on the cell surface have a more flexible evolution and easily tolerate non-synonymous mutations which, in some circumstances, can be subject to positive selection and become fixed in some populations [33]. This higher tolerance is because the function of non-viral TLRs is more redundant than of viral TLRs. Indeed, several surface TLRs are able to recognize the same bacteria and fungi components, so one microorganism can be recognized by different TLRs. Therefore, a non-synonymous mutation in one TLR does not necessarily mean the extinction of the function and does not compromise immunity [33].

The viral TLR8 has never been identified as a candidate for being under positive selection; however, our results indicate a similar level of positive selection acting in TLR8 as in the non-viral TLR4. This might be the result of the inclusion of a larger group of species that might be affected by different pathogens with implications in their PAMPs recognition. Indeed, the groups previously analyzed by others are homogeneous probably being affected by only a restricted number of related viruses which accounts for their conservation. In addition, the presence of the positive selection signature may not mean a recent event but could result from ancient functional adaptations from each species that lead to the actual taxon specificities [42]. Furthermore, as the recognition of viral RNA is essential for host defense, the mutations that could affect the function should have been removed by purifying selection and then, only the polymorphisms that are advantageous, i.e. that confer resistance to the pathogen, might have become fixed and are now reflected in the differences between species [43].

The high number of PSC observed in the non-viral TLR4 is in line with results previously reported in primates and birds [39,40]. TLR8 and TLR4 recognize very different ligands. For PAMPs recognition, TLR8 forms a homodimer that is associated with response to ssRNA viruses while the TLR4-MD-2 heterodimer mostly recognizes LPS that are present in the outer membrane of Gram-negative bacteria [13,44]. In addition, TLR4 also targets components of yeast, trypanossoma and even viruses [45]. Despite this, the reason why these two TLRs have a remarkable picture of adaptive evolution is not yet clearly understood. Some recent studies showed that in different species, the same TLR molecule recognizes specific ligands or that the same ligand triggers responses with different intensities (reviewed in [46]). For example, in rodent species, TLR8 does not respond to synthetic ligands such as imiquimod (R837), resiquimod (R848), and some guanine nucleotide analogs, as non-rodent species do [47]. This is probably caused by the variation in the surface charge and the existence of different secondary structures in different species. In addition, for TLR4, differences in ligand recognition between humans, bovines, equines and murines have also been described (reviewed in [46]). Although more studies are required to fully assess the specificity of ligand recognition and the responses that are triggered in each species, this might explain the similar patterns of evolution observed for these TLRs. The reason for the difference observed between the number of PSC in TLR7 (0.67%) and TLR8 (2.50%) is also unclear since both recognize ssRNA. A similar degree of positive selection acting in both receptors would be expected. Functional or structural differences in ligand recognition or in ligand specificity should be at the basis of the observed differences. Structural differences do exist in ligand recognition, but more studies are required. Indeed, TLR8 and TLR9 exist as preformed dimers while TLR7, along with the others TLRs, exists as monomer and just form the dimer after ligand binding [48]. Differences in tissue expression have also been observed which might account for the different pattern. Although both TLR7 and TLR8 are expressed in the lung, TLR7 is also expressed in the placenta and spleen while TLR8 is expressed also in peripheral blood leukocytes [49].

TLR1, TLR6 and TLR9 were the receptors with the lower percentage of codons under positive selection (Table 1). TLR1 has been shown to be mostly under purifying selection, but it has previously been shown to have also been subject to positive selection in chicken, contrary to the remaining avian TLRs [50], and more recently four PSC were found in the vertebrates [36]. The study of Huang and co-workers also showed evidence of one PSC in vertebrate TLR6 which was the first report of adaptive selection acting on this receptor now further supported by the present study. TLR9 has also low proportion of PSC (0.39%), but positive selection has been previously found in Primates [40] and Teleosts [51].

Seven codons were found to be under positive selection in TLR10. The study of Huang and co-workers revealed no positive selection on TLR10 [36]. Their analyses of TLR10 encompassed sixteen species, including a marsupial, a monotreme and an amphibian, which were not included in our study. In addition, their analyses only focused on PAML (CODEML) results whether ours also included the different models implemented in the Data Monkey Web Server which might explain the difference observed.

The TLR10 interacts with TLR2 to recognize triacyl lipoproteins [26,27]. In turn, TLR2 also form heterodimers with TLR1 or TLR6 to recognize the largest variety of ligands of all the TLRs (e.g. peptidoglycan, bacterial lipoproteins, zymosan, a phenol soluble factor from *Staphylococcus epidermidis*) [52]. In TLR2, six codons were found under positive selection in mammals, in line with the observation of Huang and co-workers (2011). Signatures of positive selection in this gene were also found in bovines [42], primates [53], rodents [54] and in birds [55]. The wide range of ligands as well as the need for heterodimerization (TLR1-TLR2, TLR2-TLR6, TLR6-TLR10) make the TLR2 prone to contrasting evolutionary patterns: conservation of its function, including the capacity of heterodimerization, and adaptive evolution to the environment and the pathogens specific from each species [6,36,42].

### Location and characterization of the PSC in the TLR domains

The LRRfinder software [20] was used to delimitate the functional domains of each TLR gene in order to assess the functional significance of the putatively selected sites. Human TLR sequences were used as a reference (Table 2 and Additional file 21, Table S21; Additional file 22, Table S22; Additional file 23, Table S23; Additional file 24, Table S24; Additional file 25, Table S25; Additional file 26, Table S26; Additional file 27, Table S27; Additional file 28, Table S28; Additional file 29, Table S29; Additional file 30, Table S30). The characterization of the charge and polarity of each amino acid possibility in sites under selection is also available in Additional file 31, Table S31; Additional file 32, Table S32; Additional file 33, Table S33; Additional file 34, Table S34; Additional file 35, Table S35; Additional file 36, Table S36; Additional file 37, Table S37; Additional file 38, Table S38; Additional file 39, Table S39; Additional file 40, Table S40.

**Table 2:** Identification of the domain location of each positively selected site

Number of sites under positive selection identified in each domain of gene								
**Gene**	**No. of species**	**Total no. of sites**^**a**^	**Domains**^b^					
			
			**Signal**	**LRR-NT**	**LRR**	**LRR-CT**	**Transmembrane**	**TIR**

**TLR1**	17	2	---	---	2	---	---	---

**TLR2**	23	6	1	---	3	---	1	1

**TLR3**	20	9	3	---	3	---	1	2

**TLR4**	19	22	---	---	19	1	1	1

**TLR5**	17	7	1	---	3	---	---	3

**TLR6**	20	3	---	---	---	---	2	1

**TLR7**	21	7	---	---	7	---	---	---

**TLR8**	18	26	---	1	25	---	---	---

**TLR9**	21	4	---	---	4	---	---	---

**TLR10**	13	7	---	---	5	1	---	1

^a ^total number of sites under selection.

^b ^In comparison with the delimitation of human domains of each TLR gene.

The TLRs are composed of an extracellular domain that binds the PAMPs, a signal transmembrane domain and an intracellular domain, designated the TIR domain that binds adapter molecules and that triggers the intracellular cascades leading to the innate immune response. The convex surface of the extracellular LRR domains, by being involved in the recognition of the PAMPs, is highly variable. At variance, the TIR domain is highly conserved as it is involved in the signaling cascades [56]. This suggests that the different domains of the TLR molecules are under different evolutionary pressures.

Most positively selected sites were located in the extracellular LRRs. A few instances of positive selection were detected in the remaining domains. All TLRs, with the exception of TLR6, have at least one codon under positive selection located in the LRR ectodomain and most of the PSC found within each TLR are mostly located in this domain (Table 2). The LRR ectodomain is the main point of interaction with PAMPs [11,57], which are conserved motifs [13]. Therefore, some functional constrain is expected in order to preserve the TLR ability in identifying pathogens. However, as pathogens are evolving constantly to evade host recognition, it is likely that TLRs should co-evolve with them. Our results suggest that this constant evolving nature of pathogens is accompanied by TLRs. Some species-specific substitutions may be reflected on the high number of PSC found in this domain. This could be related to the PAMPs they recognize.

In general, the LRRs are composed by a concave surface, more conservative, composed by a leucine-rich sequence, XLXXLXLXX, and by a convex surface, more exposed and more variable, XΦXXΦX4FXXLX, where X represents any amino acid and Φ a hydrophobic amino acid. Of the seventy one codons identified under positive selection in the LRRs of all TLRs, forty eight were localized in the variable segment which supports our hypothesis of co-evolution between host and pathogen (Table 2 and Additional file 21, Table S21; Additional file 22, Table S22; Additional file 23, Table S23; Additional file 24, Table S24; Additional file 25, Table S25; Additional file 26, Table S26; Additional file 27, Table S27; Additional file 28, Table S28; Additional file 29, Table S29; Additional file 30, Table S30). Interestingly, the three TLRs with more PSC in variable segment of LRRs are the viral TLR7, 8 and 9, with 71.43%, 84.0% and 75.0%, respectively. This high proportion of PSC in the variable segment of LRRs found in three viral TLRs may be indicative of the receptors' evolution shaped by viral nucleic acids (ssRNA and CpG DNA) characteristic from each species. As nucleic acids are supposed to directly interact with this convex surface, variation or evidence of positive selection in it may be the result of host adaptation to viral evolution.

The two receptors with more signatures of natural selection, TLR8 and TLR4, showed a large number of PSC in the LRR domain, even though ligand recognition is made differently [25]. Along with TLR7 and 9, TLR8 has a longer amino acid sequence in its ectodomain domain than other TLRs and contains an irregular segment of 26 to 31 amino acids between LRR14 and 15 [18]. The ectodomain is cleaved in the endolysosome to enable ligand recognition [18,58]. Thus, the functional ectodomain of human TLR8 comprises LRR15-25 and C-terminal LRR. Following ligand binding, TLR8 recruits the TIR adaptor proteins and initiates signaling [58]. Our results show that fifteen of the PSC are located in the region comprising LRR-NT-LRR14 that is cleaved. Four amino acids are located to the irregular LRR insertion before LRR15 (Table 3); however the functional importance of this region has not yet been clarified and is regarded as a new N-terminal LRR of the truncated structure [58]. Recently, it has also been described for its crucial importance in TLR8 activation, especially the Alanine substitutions in this region that can affect the activation of this receptor [47]. Alanine at the amino acid position 481 that was found to be under selective pressure may be interesting to study in greater detail. Furthermore, Govindaraj et al. (2011) proposed that this undefined region is responsible for the species-specificity in ligand recognition that is found at least between non-rodents and rodents (rodents lack the undefined region 438-442) [47]. The surface charge variation among species is crucial for the species specific pathogen recognition even though this region is not directly involved in ligand interaction [47]. In all four positions identified under selection in this irregular insertion, the amino acid possibilities may result in charge variation which might suggest a role for the specificity in ligand recognition (Additional file 38, Table S38). Of the other eight residues identified in this molecule, only one is in LRR15. LRR15 has been described for its importance in ligand recognition together with LRR17 and 18 (Table 3) [58].

**Table 3:** Positively selected sites predicted to affect TLR function based on their location in the three-dimensional structures

Gene	Position	Functional Information	Reference
**TLR1**	318	Close to sites involved in heterodimerization TLR1-TLR2 (315 and 320)	[16]
	
**TLR2**	302	LRR10 - involved in ligand binding	

**TLR3**	79	LRR2 - dsRNA-TLR3 interaction site (LRR-NT to LRR3)	[22]
		
	285	Adjacent to site of SNP (N284I) that confers partial or total loss of function	

	295	Adjacent to sites (294 and 296) involved in ligand binding	
		
	300	In the region involved in ligand binding	
		
**TLR4**	301	In the region involved in ligand binding	[23]
		
	317	In the region involved in ligand binding	
		
	363	In the region involved in ligand binding	

	441		
			
	459	Irregular LRR insertion before LRR15	[18]
			
**TLR8**	472		
	
	481	Irregular LRR insertion before LRR15 - Alanine residue mutation could affect TLR8 activation.	[18] and [47]
	
	498	LRR15 - involved in ligand binding	[18]

**Figure 1:**
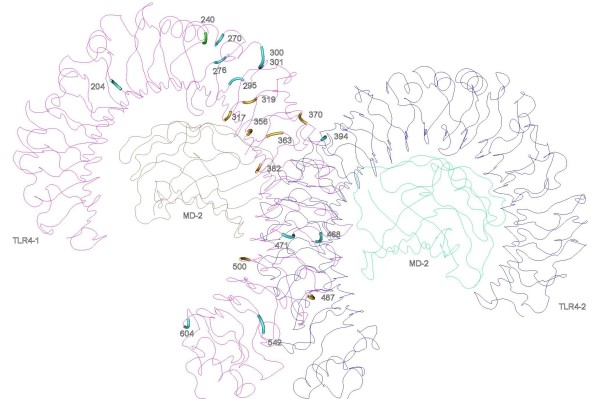
**Positively selected sites in the three-dimensional structures of TLR4**. TLR4 and myeloid differentiation factor 2 (MD-2) forms a heterodimer in which are identified the PSC detected.

The TLR4 forms a dimer with MD-2. The LPS interacts with a large hydrophobic pocket in MD-2 and directly bridges m-shaped receptor dimer composed of two copies arranged symmetrically [23]. Nineteen of the twenty two PSC in the TLR4 are located in the LRR domain. At least six of these codons have been previously identified as sites under positive selection in primates [40] and some have functional importance in PAMP recognition (Table 3) [23]. From the three-dimensional structure of the TLR4 heterodimer (Figure 1), it is possible to observe that some of the positively selected residues identified in this molecule are in the region that participates in the interaction between TLR4, MD-2 and LPS (codons 300, 301, 317, 319, 356, and 363). In all those positions, with the exception of position 356 where the three amino acid possibilities (Leu, Phe, Trp) are all conservative, amino acid substitutions might result in changes in polarity and charge (Additional file 34, Table S34). Nevertheless, the result of these variations in the function and structure of the molecule remains to be assessed. In addition, some of the identified PSC are located in close contact in the TLR4 homodimers (eg. 370, 394, 468, 471, 487, 542) and might have implications for dimerization.

**Figure 2:**
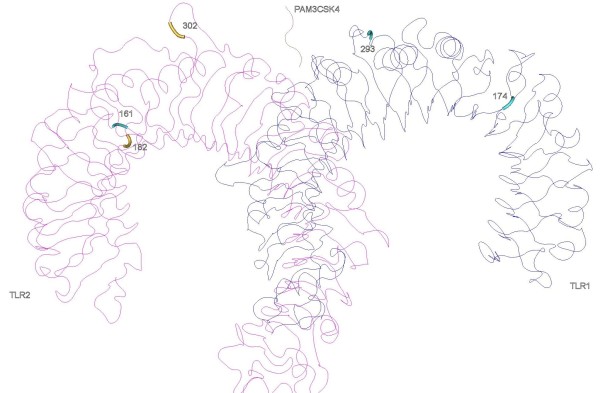
**Positively selected sites in the three-dimensional structures of TLR2-TLR1 heterodimer with the synthetic triacylated lipoprotein Pam3CSK4**.

**Figure 3:**
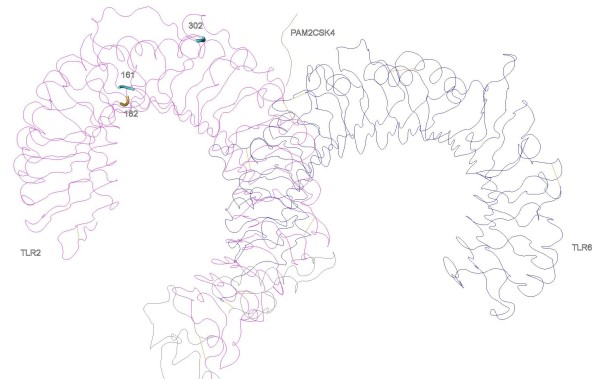
**Positively selected sites in the three-dimensional structures of TLR2-TLR6 heterodimer with the synthetic diacylated lipoprotein Pam2CSK4**.

In TLR2, three PSC lay in LRR5, 6 and 10. The LRR10 PSC is in the variable segment and this LRR has been previously described as under positive selection in bovines [42]. Despite that, these LRRs have not been recognized as sites of direct interaction with PAMPs neither involved in heterodimerization, so this result may not necessarily reflect any present functional importance but the result of ancient selective events [42]. In the heterodimers TLR1-TLR2 (Figure 2) and TLR2-TLR6 (Figure 3) only one of the identified amino acids (302 in LRR10 of the TLR2) are located in significant regions for ligand binding (LRR9-LRR12) and one (318 in TLR1) in close proximity with sites involved in heterodimerization (LRR11-LRR14) (Table 3) [16]. It is interesting to note that in TLR2, the regions identified as important for dimerization (LRR11-13) have not been subject to positive selection, which is a trace of functional conservation, particularly important as TLR2 dimerizes with three other TLRs to recognize different PAMPs [16,42].

**Figure 4:**
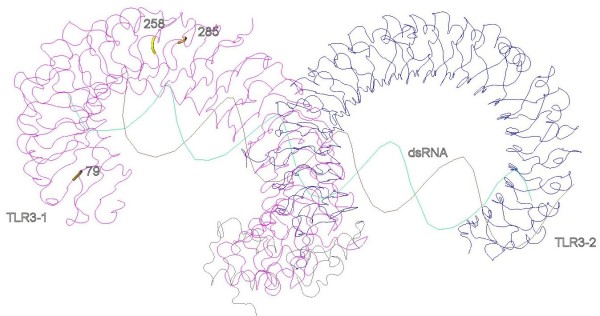
**Positively selected sites in the three-dimensional structures of TLR3 homodimer where the residues under positive selection in the ectodomain are represented**. The recognition of the dsRNA ligand is also represented.

In the viral TLR3, nine PSC were identified, 3 of which are located in the LRR domain (Figure 4). The residue 79 belongs to a dsRNA-TLR3 interaction site in the LRR-NT to LRR3 region (Table 3) [22]. In this site, five amino acid possibilities were detected along the species studied which could be a species adaptation to the recognition of specific dsRNA viruses and reflect co-evolution.

For the TLR5, three PSC were detected in the LRR domain. The residues 207 and 400 are located within the 228 amino acid region identified by Andersen-Nissen et al (2007) as important for flagellin recognition [59] and were previously reported as being under positive selection in primates [60].

The TIR domain of the TLRs is highly conserved across multiple species of animals, plants [61] and microbes [62] due to its significance as signaling domain [63]. Three Box regions of the TIR domain, which are important in signal transduction, are highly conserved in the TLRs genes (Boxes 1, 2, and 3) and should be rather under a strong purifying selection [63,64]. As expected, due to their functional constraints, we verified that none of the nine sites identified as under positive selection in the TIR domain were located in these boxes. This observation is expected as these boxes, due to their functional constraints, should be rather under a strong purifying selection [63,64].

The TLR with more PSC in the TIR domain was TLR5 where three codons under selection were located within the highly conserved TIR domain and although amino acid alterations at codon 674 are conservative, alterations at codons 721 and 742 might induce differences in charge and polarity of the protein, respectively (Additional file 35, Table S35). Despite the expected functional constraints specific of this domain, it seems that the TLR5 protein may present some flexibility with regards to amino acid composition in this domain. In human, TLR5 has been suggested to be functionally redundant. Indeed, TLR5^392STOP ^is a non-functional allele that may reach considerable frequencies in some human populations (up to 23%; [65]) despite increasing the susceptibility to the Legionnaires' disease. Positive selection has been proposed as a mechanism for favoring gene loss in human evolution [66,67]. This suggests that other proteins exist, yet to be determined, that might be able to compensate for a loss of function of this TLR.

In the remaining domains a few PSCs were identified although they can have functional or evolutionary importance. Indeed, in the signal domain, we identified five PSCs. This domain mostly mediates or regulates the transport of the secretory proteins to their destination compartment in the cell [68]. Given the role of this domain, it is likely that these five amino acids might interfere with the correct location of the secretory proteins in the different cell compartments. In the LRR- NT domain we identified one PSC in TLR8. This domain is known for its importance in stabilizing the LRR structure by protecting the hydrophobic core [18]. However, in the TLR8 molecule this domain is cleaved along with LRR1-14 to enable ligand recognition [18,58], thus no particular importance can be attributed. In the 3' end of the LRR structure we find the LRR-CT where two PSC were found. The role of this domain is similar to LRR-NT so these residues might be important for the stabilization of the molecule in particular for the formation of the dimers TLR4-MD2 and TLR10-TLR2 [18,69]. In the PSC identified in the LRR-CT, amino acid variation between species does not alter the charge (Additional file 34, Table S34; Additional file 40, Table S40). This also happens in all five positions under positive selection in the transmembrane domain of TLR 2, 3, 4 and 6 (Additional file 32, Table S32; Additional file 33, Table S33; Additional file 34, Table S34; Additional file 36, Table S36). The transmembrane segment is responsible for the junction between the TLR and the plasmatic membrane. In some cases, it is also associated with the localization of the TLR in intracellular compartments and its interaction with accessory molecules [70,71]. This domain is expected to be highly conserved and only few mutations have been described [72,73]. Nevertheless, we found five codons under positive selection in this domain.

In summary, pathogens usually develop strategies to evade recognition by the host immune system. Therefore, motifs in the pathogen that are involved in the recognition tend to evolve faster to avoid this recognition. If the pathogen is evolving, the receptor that recognizes the pathogen should also evolve to keep pace with the changes that occur in the pathogen. This arms-race is responsible for this continuum of alterations in both the pathogen and the receptor which can be detected as signatures of positive selection. For example, positive selection in RNA viruses has been shown to occur as the result of this arms-race [74,75]. Thus, changes in the sequence of RNA or DNA of the pathogen will cause the receptor not to recognize them. This will force alterations in the receptor which might change the geometry of the interaction. For this reason, if different ligands (i.e. pathogens) are recognized by one receptor, it is likely that more changes are observed than for receptors that recognize only one ligand. In line with this, our results may reflect the co-evolution between each host and its pathogens and commensals, especially in viral TLRs (due to the wide variety of viruses they might recognize) and in the non-viral TLR4. In addition, the fact that the mutation rates for RNA and DNA viruses tend to be generally higher than for bacteria and yeast [76] also correlates with our results. Viral TLRs will thus encounter ligands that evolve faster than non-viral TLR. TLR4 might appear as an exception due to the wide variety of ligands that it recognizes, which include viruses.

## Conclusions

Evidence of positive selection was found for all the mammalian TLRs studied. Adaptive selection has clearly played a role in shaping the diversity of both viral and non-viral TLRs. Location of some of the positively selected codons indicate that pathogens exert most of the selective pressures that lead to the changes observed mostly in the LRR ectodomain, especially in its variable segment responsible for direct interaction with PAMPS. This suggests that they are the result of co-evolution. Further studies are important to clarify the ligand for each TLR in each species as it could give new clues for the interpretation of these results. Also, crystallographic studies would be helpful for assessing the functional relevance of the PSCs detected.

## Methods

### Sequences

The sequences of the mammalian TLRs used in the analyses were retrieved from GenBank (http://www.ncbi.nlm.nih.gov/genbank/) and Ensembl (http://www.ensembl.org/index.html). For each TLR, a subset of 13-23 species was used, that included species from some of the most representative mammalian groups, Artiodactyla, Rodents, Carnivores, Lagomorphs and Primates. Lagomorphs were not included in the TLR7, 8 and 9 analyses due to the lack of data. The identification of the species used for each TLR and the accession numbers are presented in Additional files (Additional file 1, Table S1; Additional file 2, Table S2; Additional file 3, Table S3; Additional file 4, Table S4; Additional file 5, Table S5; Additional file 6, Table S6; Additional file 7, Table S7; Additional file 8, Table S8; Additional file 9, Table S9; Additional file 10, Table S10).

### Codon-based analyses of positive selection

Under neutrality, coding sequences are expected to present a ratio of non-synonymous substitutions (d_N_) over synonymous substitutions (d_S_) that does not significantly deviate from 1 (ω = d_N_/d_S _= 1) while significant deviations may be interpreted as either the result of positive selection (ω >> 1) or of negative selection (ω << 1).

To test for positive selection in individual codons of mammalian TLR sequences, the d_N _to d_S _ratios were compared using two maximum likelihood (ML) frameworks, the Hyphy package implemented in the Data Monkey Web Server (http://www.datamonkey.org[77] and the CODEML (PAML version 4) [78] as proposed by Wlasiuk and Nachman (2010).

In the Data Monkey Web Server, the best fitting nucleotide substitution model was searched for through the automatic model selection tool available on the server. All sequences of each TLR were analyzed under three distinct models, single likelihood ancestor counting [64], fixed-effect likelihood (FEL) and random effect likelihood (REL). The SLAC model is based on the reconstruction of the ancestral sequences and the counts of *d*_*S*_, *d*_*N *_at each codon position of the phylogeny. The FEL model estimates the ratio of *d*_*N*_/*d*_*S *_on a site-by-site basis, without assuming an *a priori *distribution across sites. The REL model first fits a distribution of rates across sites and then infers the substitution rate for individual sites. The criteria to identify codons under positive selection were the same used by Wlasiuk and Nachman (2010). Sites with P values <0.1 for SLAC and FEL, and Bayes Factor >50 for REL were considered as candidates to be under positive selection.

In CODEML, two alternative models M7 and M8 were implemented. M7 only allows codons to evolve neutrally or under purifying selection while M8 adds a class of sites under positive selection. The two previous nested models were compared using the likelihood ratio test (LRT) with 2 degrees of freedom [79]. Amino acids under selection for M8 were identified using Bayes Empirical Bayes approach (BEB) with posterior probability >90%. For each gene, a neighbor-joining tree was used as the working topology which was constructed using Mega 5 [80] with the options p-distance as the substitution model and complete deletion to gaps and missing data.

In accordance to the methodology adopted in Wlasiuk and Nachman (2010), only sites identified as under positive selection by more than one ML method were considered. The amino acid possibilities were characterized with regards to polarity, charge and location in the protein, and are described in the Additional file 31, Table S31; Additional file 32, Table S32; Additional file 33, Table S33; Additional file 34, Table S34; Additional file 35, Table S35; Additional file 36, Table S36; Additional file 37, Table S37; Additional file 38, Table S38; Additional file 39, Table S39; Additional file 40, Table S40.

### Identification of domains

To determine the delimitation of each of the domains of the TLR molecules, the LRRfinder software was used [20] (http://www.lrrfinder.com/). The human delimitations were used as a reference for the remaining species (Additional file 21, Table S21; Additional file 22, Table S22; Additional file 23, Table S23; Additional file 24, Table S24; Additional file 25, Table S25; Additional file 26, Table S26; Additional file 27, Table S27; Additional file 28, Table S28; Additional file 29, Table S29; Additional file 30, Table S30).

Crystal structures or theoretical models were used, when available and relevant, to map the PSC onto the protein three-dimensional structures using the NCBI application Cn3D [81]. The following models were used: TLR1-TLR2 (MMDB ID: 59994) [16]; TLR2-TLR6 (MMDB ID: 78279) [82]; TLR3 (MMDB ID: 64341) [22]; TLR4 (MMDB ID: 70004) [23].

## List of abbreviations

BEB: Bayes Empirical Bayes; CpG: Cytosine Phosphate Guanine; CT: Carboxy termini; d_N_: Non-synonymous substitutions; d_S_: Synonymous substitutions; dsRNA: Double-strand RNA; FEL: Fixed-effect likelihood; IL-18R: Human IL-18 receptor; IL1R: Human interleukin-1 receptor; LPS: Lipopolysaccharide; LRR: Leucine rich repeats; LRT: Likelihood ratio test; MD-2: Myeloid differentiation factor 2; ML: Maximum-Likelihood; NT: Amino termini; PAMP: Pathogen-associated molecular patterns; PRR: Pattern recognition receptors; PSC: Positively selected codons; REL: Random effect likelihood; SLAC: Single likelihood ancestor counting; ssRNA: Single-strand RNA; TIR: Highly conserved cytoplasmic domain homologous to the IL1R and IL-18R; TLR: Toll-like receptor

## Authors' contributions

HA carried out the analyses performed in the study and drafted the manuscript. JA participated in the study design and coordination and helped to draft the manuscript. PJE conceived of the study, participated in its design and coordination and helped to draft the manuscript by revising it critically. All authors read and approved the final manuscript.

## Supplementary Material

Additional file 1 **Table S1. Identification of the sequences used for the TLR1 alignment**. Microsoft Word document containing the list of accession numbers of the sequences used for the TLR1 alignment. Click here for file

Additional file 2 **Table S2. Identification of the sequences used for the TLR2 alignment**. Microsoft Word document containing the list of accession numbers of the sequences used for the TLR2 alignment. Click here for file

Additional file 3 **Table S3. Identification of the sequences used for the TLR3 alignment**. Microsoft Word document containing the list of accession numbers of the sequences used for the TLR3 alignment. Click here for file

Additional file 4 **Table S4. Identification of the sequences used for the TLR4 alignment**. Microsoft Word document containing the list of accession numbers of the sequences used for the TLR4 alignment. Click here for file

Additional file 5 **Table S5. Identification of the sequences used for the TLR5 alignment**. Microsoft Word document containing the list of accession numbers of the sequences used for the TLR5 alignment. Click here for file

Additional file 6 **Table S6. Identification of the sequences used for the TLR6 alignment**. Microsoft Word document containing the list of accession numbers of the sequences used for the TLR6 alignment. Click here for file

Additional file 7 **Table S7. Identification of the sequences used for the TLR7 alignment**. Microsoft Word document containing the list of accession numbers of the sequences used for the TLR7 alignment. Click here for file

Additional file 8 **Table S8. Identification of the sequences used for the TLR8 alignment**. Microsoft Word document containing the list of accession numbers of the sequences used for the TLR8 alignment. Click here for file

Additional file 9 **Table S9. Identification of the sequences used for the TLR9 alignment**. Microsoft Word document containing the list of accession numbers of the sequences used for the TLR9 alignment. Click here for file

Additional file 10 **Table S10. Identification of the sequences used for the TLR10 alignment**. Microsoft Word document containing the list of accession numbers of the sequences used for the TLR10 alignment. Click here for file

Additional file 11 **Table S11. Amino acid alterations found in TLR1 for each species at each positively selected site**. Microsoft Word document containing the amino acid alterations at each site under selection in TLR1 gene. Click here for file

Additional file 12 **Table S12. Amino acid alterations found in TLR2 for each species at each positively selected site**. Microsoft Word document containing the amino acid alterations at each site under selection in TLR2 gene. Click here for file

Additional file 13 **Table S13. Amino acid alterations found in TLR3 for each species at each positively selected site**. Microsoft Word document containing the amino acid alterations at each site under selection in TLR3 gene. Click here for file

Additional file 14 **Table S14. Amino acid alterations found in TLR4 for each species at each positively selected site**. Microsoft Word document containing the amino acid alterations at each site under selection in TLR4 gene. Click here for file

Additional file 15 **Table S15. Amino acid alterations found in TLR5 for each species at each positively selected site**. Microsoft Word document containing the amino acid alterations at each site under selection in TLR5 gene. Click here for file

Additional file 16 **Table S16. Amino acid alterations found in TLR6 for each species at each positively selected site**. Microsoft Word document containing the amino acid alterations at each site under selection in TLR6 gene. Click here for file

Additional file 17 **Table S17. Amino acid alterations found in TLR7 for each species at each positively selected site**. Microsoft Word document containing the amino acid alterations at each site under selection in TLR7 gene. Click here for file

Additional file 18 **Table S18. Amino acid alterations found in TLR8 for each species at each positively selected site**. Microsoft Word document containing the amino acid alterations at each site under selection in TLR8 gene. Click here for file

Additional file 19 **Table S19. Amino acid alterations found in TLR9 for each species at each positively selected site**. Microsoft Word document containing the amino acid alterations at each site under selection in TLR9 gene. Click here for file

Additional file 20 **Table S20. Amino acid alterations found in TLR10 for each species at each positively selected site**. Microsoft Word document containing the amino acid alterations at each site under selection in TLR10 gene. Click here for file

Additional file 21 **Table S21. Domain characterization of TLR1**. Microsoft Word document containing the list of domains of Human TLR1 gene, their delimitation and sequence. Click here for file

Additional file 22 **Table S22. Domain characterization of TLR2**. Microsoft Word document containing the list of domains of Human TLR2 gene, their delimitation and sequence. Click here for file

Additional file 23 **Table S23. Domain characterization of TLR3**. Microsoft Word document containing the list of domains of Human TLR3 gene, their delimitation and sequence. Click here for file

Additional file 24 **Table S24. Domain characterization of TLR4**. Microsoft Word document containing the list of domains of Human TLR4 gene, their delimitation and sequence. Click here for file

Additional file 25 **Table S25. Domain characterization of TLR5**. Microsoft Word document containing the list of domains of Human TLR5 gene, their delimitation and sequence. Click here for file

Additional file 26 **Table S26. Domain characterization of TLR6**. Microsoft Word document containing the list of domains of Human TLR6 gene, their delimitation and sequence. Click here for file

Additional file 27 **Table S27. Domain characterization of TLR7**. Microsoft Word document containing the list of domains of Human TLR7 gene, their delimitation and sequence. Click here for file

Additional file 28 **Table S28. Domain characterization of TLR8**. Microsoft Word document containing the list of domains of Human TLR8 gene, their delimitation and sequence. Click here for file

Additional file 29 **Table S29. Domain characterization of TLR9**. Microsoft Word document containing the list of domains of Human TLR9 gene, their delimitation and sequence. Click here for file

Additional file 30 **Table S30. Domain characterization of TLR10**. Microsoft Word document containing the list of domains of Human TLR10 gene, their delimitation and sequence. Click here for file

Additional file 31 **Table S31. Characterization of the amino acids possibilities for each residue identified under positive selection in TLR1**. Microsoft Word document containing the list of PSC in TLR1 and their localization in domains of Human TLR1 gene. For each site, we list the amino acid possibilities and the characterization of their polarity and charge. Click here for file

Additional file 32 **Table S32. Characterization of the amino acids possibilities for each residue identified under positive selection in TLR2**. Microsoft Word document containing the list of PSC in TLR2 and their localization in domains of Human TLR2 gene. For each site, we list the amino acid possibilities and the characterization of their polarity and charge. Click here for file

Additional file 33 **Table S33. Characterization of the amino acids possibilities for each residue identified under positive selection in TLR3**. Microsoft Word document containing the list of PSC in TLR3 and their localization in domains of Human TLR3 gene. For each site, we list the amino acid possibilities and the characterization of their polarity and charge. Click here for file

Additional file 34 **Table S34. Characterization of the amino acids possibilities for each residue identified under positive selection in TLR4**. Microsoft Word document containing the list of PSC in TLR4 and their localization in domains of Human TLR4 gene. For each site, we list the amino acid possibilities and the characterization of their polarity and charge. Click here for file

Additional file 35 **Table S35. Characterization of the amino acids possibilities for each residue identified under positive selection in TLR5**. Microsoft Word document containing the list of PSC in TLR5 and their localization in domains of Human TLR5 gene. For each site, we list the amino acid possibilities and the characterization of their polarity and charge. Click here for file

Additional file 36 **Table S36. Characterization of the amino acids possibilities for each residue identified under positive selection in TLR6**. Microsoft Word document containing the list of PSC in TLR6 and their localization in domains of Human TLR6 gene. For each site, we list the amino acid possibilities and the characterization of their polarity and charge. Click here for file

Additional file 37 **Table S37. Characterization of the amino acids possibilities for each residue identified under positive selection in TLR7**. Microsoft Word document containing the list of PSC in TLR7 and their localization in domains of Human TLR7 gene. For each site, we list the amino acid possibilities and the characterization of their polarity and charge. Click here for file

Additional file 38 **Table S38. Characterization of the amino acids possibilities for each residue identified under positive selection in TLR8**. Microsoft Word document containing the list of PSC in TLR8 and their localization in domains of Human TLR8 gene. For each site, we list the amino acid possibilities and the characterization of their polarity and charge. Click here for file

Additional file 39 **Table S39. Characterization of the amino acids possibilities for each residue identified under positive selection in TLR9**. Microsoft Word document containing the list of PSC in TLR9 and their localization in domains of Human TLR9 gene. For each site, we list the amino acid possibilities and the characterization of their polarity and charge. Click here for file

Additional file 40 **Table S40. Characterization of the amino acids possibilities for each residue identified under positive selection in TLR10**. Microsoft Word document containing the list of PSC in TLR10 and their localization in domains of Human TLR10 gene. For each site, we list the amino acid possibilities and the characterization of their polarity and charge. Click here for file

## References

[B1] O'Neill LA Bryant CE Doyle SL Therapeutic targeting of Toll-like receptors for infectious and inflammatory diseases and cancer Pharmacol Rev 2009 61 177 197 10.1124/pr.109.001073 19474110 PMC2846156

[B2] Rakoff-Nahoum S Medzhitov R Toll-like receptors and cancer Nat Rev Cancer 2009 9 57 63 10.1038/nrc2541 19052556

[B3] Barton GM Kagan JC A cell biological view of Toll-like receptor function: regulation through compartmentalization Nat Rev Immunol 2009 9 535 542 10.1038/nri2587 19556980 PMC3934928

[B4] Akira S Uematsu S Takeuchi O Pathogen recognition and innate immunity Cell 2006 124 783 801 10.1016/j.cell.2006.02.015 16497588

[B5] Takeuchi O Akira S Pattern recognition receptors and inflammation Cell 2010 140 805 820 10.1016/j.cell.2010.01.022 20303872

[B6] Roach JC Glusman G Rowen L Kaur A Purcell MK Smith KD Hood LE Aderem A The evolution of vertebrate Toll-like receptors Proc Natl Acad Sci USA 2005 102 9577 9582 10.1073/pnas.0502272102 15976025 PMC1172252

[B7] Takeda K Kaisho T Akira S Toll-like receptors Annu Rev Immunol 2003 21 335 376 10.1146/annurev.immunol.21.120601.141126 12524386

[B8] Carty M Bowie AG Recent insights into the role of Toll-like receptors in viral infection Clin Exp Immunol 2010 10.1111/j.1365-2249.2010.04196.x PMC2962956 20560984

[B9] Chaturvedi APS How location governs toll-like receptor signaling Traffic 2009 10 621 628 10.1111/j.1600-0854.2009.00899.x 19302269 PMC2741634

[B10] Barton GM Viral recognition by Toll-like receptors Semin Immunol 2007 19 33 40 10.1016/j.smim.2007.01.003 17336545

[B11] Bell JK Mullen GE Leifer CA Mazzoni A Davies DR Segal DM Leucine-rich repeats and pathogen recognition in Toll-like receptors Trends Immunol 2003 24 528 533 10.1016/S1471-4906(03)00242-4 14552836

[B12] Beutler B Jiang Z Georgel P Crozat K Croker B Rutschmann S Du X Hoebe K Genetic analysis of host resistance: Toll-like receptor signaling and immunity at large Annu Rev Immunol 2006 24 353 389 10.1146/annurev.immunol.24.021605.090552 16551253

[B13] Medzhitov R Janeway CA Jr Innate immunity: the virtues of a nonclonal system of recognition Cell 1997 91 295 298 10.1016/S0092-8674(00)80412-2 9363937

[B14] Bhattacharjee RN Akira S Toll-Like Receptor Signaling: Emerging Opportunities in Human Diseases and Medicine Current Immunology Reviews 2005 81 90

[B15] Zhu J Mohan C Toll-like receptor signaling pathways--therapeutic opportunities Mediators Inflamm 2010 2010 781235 20981241 10.1155/2010/781235 PMC2963142

[B16] Jin MS Kim SE Heo JY Lee ME Kim HM Paik SG Lee H Lee JO Crystal structure of the TLR1-TLR2 heterodimer induced by binding of a tri-acylated lipopeptide Cell 2007 130 1071 1082 10.1016/j.cell.2007.09.008 17889651

[B17] Kim HM Park BS Kim JI Kim SE Lee J Oh SC Enkhbayar P Matsushima N Lee H Yoo OJ Lee JO Crystal structure of the TLR4-MD-2 complex with bound endotoxin antagonist Eritoran Cell 2007 130 906 917 10.1016/j.cell.2007.08.002 17803912

[B18] Wei T Gong J Jamitzky F Heckl WM Stark RW Rossle SC Homology modeling of human Toll-like receptors TLR7, 8, and 9 ligand-binding domains Protein Sci 2009 18 1684 1691 10.1002/pro.186 19521997 PMC2776956

[B19] Letunic I Doerks T Bork P SMART 6: recent updates and new developments Nucleic Acids Res 2009 37 D229 232 10.1093/nar/gkn808 18978020 PMC2686533

[B20] Offord V Coffey TJ Werling D LRRfinder: a web application for the identification of leucine-rich repeats and an integrative Toll-like receptor database Dev Comp Immunol 2010 34 1035 1041 10.1016/j.dci.2010.05.004 20470819

[B21] Choe J Kelker MS Wilson IA Crystal structure of human toll-like receptor 3 (TLR3) ectodomain Science 2005 309 581 585 10.1126/science.1115253 15961631

[B22] Liu L Botos I Wang Y Leonard JN Shiloach J Segal DM Davies DR Structural basis of toll-like receptor 3 signaling with double-stranded RNA Science 2008 320 379 381 10.1126/science.1155406 18420935 PMC2761030

[B23] Park BS Song DH Kim HM Choi BS Lee H Lee JO The structural basis of lipopolysaccharide recognition by the TLR4-MD-2 complex Nature 2009 458 1191 1195 10.1038/nature07830 19252480

[B24] Chang ZL Important aspects of Toll-like receptors, ligands and their signaling pathways Inflamm Res 2010 59 791 808 10.1007/s00011-010-0208-2 20593217

[B25] Uematsu S Akira S Toll-Like receptors (TLRs) and their ligands Handb Exp Pharmacol 2008 1 20 10.1007/978-3-540-72167-3_1 18071652

[B26] Guan Y Ranoa DR Jiang S Mutha SK Li X Baudry J Tapping RI Human TLRs 10 and 1 share common mechanisms of innate immune sensing but not signaling J Immunol 2010 184 5094 5103 10.4049/jimmunol.0901888 20348427

[B27] Hasan U Chaffois C Gaillard C Saulnier V Merck E Tancredi S Guiet C Briere F Vlach J Lebecque S Human TLR10 is a functional receptor, expressed by B cells and plasmacytoid dendritic cells, which activates gene transcription through MyD88 J Immunol 2005 174 2942 2950 15728506 10.4049/jimmunol.174.5.2942

[B28] Yoneyama M Fujita T Recognition of viral nucleic acids in innate immunity Rev Med Virol 2010 20 4 22 10.1002/rmv.633 20041442

[B29] Alexopoulou L Holt AC Medzhitov R Flavell RA Recognition of double-stranded RNA and activation of NF-kappaB by Toll-like receptor 3 Nature 2001 413 732 738 10.1038/35099560 11607032

[B30] Takeuchi O Akira S Recognition of viruses by innate immunity Immunol Rev 2007 220 214 224 10.1111/j.1600-065X.2007.00562.x 17979849

[B31] Janssens S Beyaert R Role of Toll-like receptors in pathogen recognition Clin Microbiol Rev 2003 16 637 646 10.1128/CMR.16.4.637-646.2003 14557290 PMC207104

[B32] Mukherjee S Sarkar-Roy N Wagener DK Majumder PP Signatures of natural selection are not uniform across genes of innate immune system, but purifying selection is the dominant signature Proc Natl Acad Sci USA 2009 106 7073 7078 10.1073/pnas.0811357106 19359493 PMC2678448

[B33] Barreiro LB Ben-Ali M Quach H Laval G Patin E Pickrell JK Bouchier C Tichit M Neyrolles O Gicquel B Evolutionary dynamics of human Toll-like receptors and their different contributions to host defense PLoS Genet 2009 5 e1000562 10.1371/journal.pgen.1000562 19609346 PMC2702086

[B34] Ortiz M Kaessmann H Zhang K Bashirova A Carrington M Quintana-Murci L Telenti A The evolutionary history of the CD209 (DC-SIGN) family in humans and non-human primates Genes Immun 2008 9 483 492 10.1038/gene.2008.40 18528403 PMC2701223

[B35] Nakajima T Ohtani H Satta Y Uno Y Akari H Ishida T Kimura A Natural selection in the TLR-related genes in the course of primate evolution Immunogenetics 2008 60 727 735 10.1007/s00251-008-0332-0 18810425

[B36] Huang Y Temperley ND Ren L Smith J Li N Burt DW Molecular evolution of the vertebrate TLR1 gene family - a complex history of gene duplication, gene conversion, positive selection and co-evolution BMC Evol Biol 2011 11 149 10.1186/1471-2148-11-149 21619680 PMC3125219

[B37] Hamblin MT Thompson EE Di Rienzo A Complex signatures of natural selection at the Duffy blood group locus Am J Hum Genet 2002 70 369 383 10.1086/338628 11753822 PMC419988

[B38] Vallender EJ Lahn BT Positive selection on the human genome Hum Mol Genet 2004 13 Spec No 2 R245 254 15358731 10.1093/hmg/ddh253

[B39] Alcaide M Edwards SV Molecular evolution of the Toll-like receptor multigene family in birds Mol Biol Evol 2011 10.1093/molbev/msq351 21239391

[B40] Wlasiuk G Nachman MW Adaptation and constraint at Toll-like receptors in primates Mol Biol Evol 2010 27 2172 2186 10.1093/molbev/msq104 20410160 PMC3107592

[B41] Rassa JC Ross SR Viruses and Toll-like receptors Microbes Infect 2003 5 961 968 10.1016/S1286-4579(03)00193-X 12941388

[B42] Jann OC Werling D Chang JS Haig D Glass EJ Molecular evolution of bovine Toll-like receptor 2 suggests substitutions of functional relevance BMC Evol Biol 2008 8 288 10.1186/1471-2148-8-288 18937834 PMC2588590

[B43] Morozumi T Uenishi H Polymorphism distribution and structural conservation in RNA-sensing Toll-like receptors 3, 7, and 8 in pigs Biochim Biophys Acta 2009 10.1016/j.bbagen.2009.01.002 19714804

[B44] Shimazu R Akashi S Ogata H Nagai Y Fukudome K Miyake K Kimoto M MD-2, a molecule that confers lipopolysaccharide responsiveness on Toll-like receptor 4 J Exp Med 1999 189 1777 1782 10.1084/jem.189.11.1777 10359581 PMC2193086

[B45] Kumar H Kawai T Akira S Toll-like receptors and innate immunity Biochem Biophys Res Commun 2009 388 621 625 10.1016/j.bbrc.2009.08.062 19686699

[B46] Werling D Jann OC Offord V Glass EJ Coffey TJ Variation matters: TLR structure and species-specific pathogen recognition Trends Immunol 2009 30 124 130 10.1016/j.it.2008.12.001 19211304

[B47] Govindaraj RG Manavalan B Basith S Choi S Comparative analysis of species-specific ligand recognition in Toll-like receptor 8 signaling: a hypothesis PLoS One 2011 6 e25118 10.1371/journal.pone.0025118 21949866 PMC3176813

[B48] Manavalan B Basith S Choi S Similar Structures but Different Roles - An Updated Perspective on TLR Structures Front Physiol 2011 2 41 21845181 10.3389/fphys.2011.00041 PMC3146039

[B49] Du X Poltorak A Wei Y Beutler B Three novel mammalian toll-like receptors: gene structure, expression, and evolution Eur Cytokine Netw 2000 11 362 371 11022119

[B50] Downing T Lloyd AT O'Farrelly C Bradley DG The differential evolutionary dynamics of avian cytokine and TLR gene classes J Immunol 2010 184 6993 7000 10.4049/jimmunol.0903092 20483729

[B51] Chen JS Wang TY Tzeng TD Wang CY Wang D Evidence for positive selection in the TLR9 gene of teleosts Fish Shellfish Immunol 2008 24 234 242 10.1016/j.fsi.2007.11.005 18164212

[B52] Janeway CA Jr Medzhitov R Innate immune recognition Annu Rev Immunol 2002 20 197 216 10.1146/annurev.immunol.20.083001.084359 11861602

[B53] Takaki A Yamazaki A Maekawa T Shibata H Hirayama K Kimura A Hirai H Yasunami M Positive selection of Toll-like receptor 2 polymorphisms in two closely related old world monkey species, rhesus and Japanese macaques Immunogenetics 2011 10.1007/s00251-011-0556-2 21744114

[B54] Tschirren B Raberg L Westerdahl H Signatures of selection acting on the innate immunity gene Toll-like receptor 2 (TLR2) during the evolutionary history of rodents J Evol Biol 2011 24 1232 1240 10.1111/j.1420-9101.2011.02254.x 21418116

[B55] Cormican P Lloyd AT Downing T Connell SJ Bradley D O'Farrelly C The avian Toll-Like receptor pathway--subtle differences amidst general conformity Dev Comp Immunol 2009 33 967 973 10.1016/j.dci.2009.04.001 19539094

[B56] Beutler B Rehli M Evolution of the TIR, tolls and TLRs: functional inferences from computational biology Curr Top Microbiol Immunol 2002 270 1 21 10.1007/978-3-642-59430-4_1 12467241

[B57] Kobe B Deisenhofer J A structural basis of the interactions between leucine-rich repeats and protein ligands Nature 1995 374 183 186 10.1038/374183a0 7877692

[B58] Ewald SE Lee BL Lau L Wickliffe KE Shi GP Chapman HA Barton GM The ectodomain of Toll-like receptor 9 is cleaved to generate a functional receptor Nature 2008 456 658 662 10.1038/nature07405 18820679 PMC2596276

[B59] Andersen-Nissen E Smith KD Bonneau R Strong RK Aderem A A conserved surface on Toll-like receptor 5 recognizes bacterial flagellin J Exp Med 2007 204 393 403 10.1084/jem.20061400 17283206 PMC2118731

[B60] Wlasiuk G Khan S Switzer WM Nachman MW A history of recurrent positive selection at the toll-like receptor 5 in primates Mol Biol Evol 2009 26 937 949 10.1093/molbev/msp018 19179655 PMC2734149

[B61] Jebanathirajah JA Peri S Pandey A Toll and interleukin-1 receptor (TIR) domain-containing proteins in plants: a genomic perspective Trends Plant Sci 2002 7 388 391 10.1016/S1360-1385(02)02309-9 12234729

[B62] Turner JD A bioinformatic approach to the identification of bacterial proteins interacting with Toll-interleukin 1 receptor-resistance (TIR) homology domains FEMS Immunol Med Microbiol 2003 37 13 21 10.1016/S0928-8244(03)00095-6 12770755

[B63] Xu Y Tao X Shen B Horng T Medzhitov R Manley JL Tong L Structural basis for signal transduction by the Toll/interleukin-1 receptor domains Nature 2000 408 111 115 10.1038/35040600 11081518

[B64] Slack JL Schooley K Bonnert TP Mitcham JL Qwarnstrom EE Sims JE Dower SK Identification of two major sites in the type I interleukin-1 receptor cytoplasmic region responsible for coupling to pro-inflammatory signaling pathways J Biol Chem 2000 275 4670 4678 10.1074/jbc.275.7.4670 10671496

[B65] Hawn TR Verbon A Lettinga KD Zhao LP Li SS Laws RJ Skerrett SJ Beutler B Schroeder L Nachman A A common dominant TLR5 stop codon polymorphism abolishes flagellin signaling and is associated with susceptibility to legionnaires' disease J Exp Med 2003 198 1563 1572 10.1084/jem.20031220 14623910 PMC2194120

[B66] Olson MV When less is more: gene loss as an engine of evolutionary change Am J Hum Genet 1999 64 18 23 10.1086/302219 9915938 PMC1377697

[B67] Olson MV Varki A Sequencing the chimpanzee genome: insights into human evolution and disease Nat Rev Genet 2003 4 20 28 10.1038/nrg981 12509750

[B68] Choo KH Tan TW Ranganathan S A comprehensive assessment of N-terminal signal peptides prediction methods BMC Bioinformatics 2009 10 Suppl 15 S2 10.1186/1471-2105-10-S15-S2 19958512 PMC2788353

[B69] Botos I Liu L Wang Y Segal DM Davies DR The toll-like receptor 3:dsRNA signaling complex Biochim Biophys Acta 2009 1789 667 674 19595807 10.1016/j.bbagrm.2009.06.005 PMC2784288

[B70] Brinkmann MM Spooner E Hoebe K Beutler B Ploegh HL Kim YM The interaction between the ER membrane protein UNC93B and TLR3, 7, and 9 is crucial for TLR signaling J Cell Biol 2007 177 265 275 10.1083/jcb.200612056 17452530 PMC2064135

[B71] Nishiya T Kajita E Miwa S Defranco AL TLR3 and TLR7 are targeted to the same intracellular compartments by distinct regulatory elements J Biol Chem 2005 280 37107 37117 10.1074/jbc.M504951200 16105838

[B72] Cargill EJ Womack JE Detection of polymorphisms in bovine toll-like receptors 3, 7, 8, and 9 Genomics 2007 89 745 755 10.1016/j.ygeno.2007.02.008 17442537

[B73] Mikula I Bhide M Pastorekova S Characterization of ovine TLR7 and TLR8 protein coding regions, detection of mutations and Maedi Visna virus infection Vet Immunol Immunopathol 2010 138 51 59 10.1016/j.vetimm.2010.06.015 20638136

[B74] Esteves PJ Abrantes J Carneiro M Muller A Thompson G van der Loo W Detection of positive selection in the major capsid protein VP60 of the rabbit haemorrhagic disease virus (RHDV) Virus Res 2008 137 253 256 10.1016/j.virusres.2008.07.025 18761043

[B75] Snoeck J Fellay J Bartha I Douek DC Telenti A Mapping of positive selection sites in the HIV-1 genome in the context of RNA and protein structural constraints Retrovirology 2011 8 87 10.1186/1742-4690-8-87 22044801 PMC3229471

[B76] Drake JW The distribution of rates of spontaneous mutation over viruses, prokaryotes, and eukaryotes Ann N Y Acad Sci 1999 870 100 107 10.1111/j.1749-6632.1999.tb08870.x 10415476

[B77] Pond SL Frost SD Datamonkey: rapid detection of selective pressure on individual sites of codon alignments Bioinformatics 2005 21 2531 2533 10.1093/bioinformatics/bti320 15713735

[B78] Yang Z PAML 4: phylogenetic analysis by maximum likelihood Mol Biol Evol 2007 24 1586 1591 10.1093/molbev/msm088 17483113

[B79] Yang Z Nielsen R Goldman N Pedersen AM Codon-substitution models for heterogeneous selection pressure at amino acid sites Genetics 2000 155 431 449 10790415 10.1093/genetics/155.1.431 PMC1461088

[B80] Tamura K Peterson D Peterson N Stecher G Nei M Kumar S MEGA5: Molecular Evolutionary Genetics Analysis using Maximum Likelihood, Evolutionary Distance, and Maximum Parsimony Methods Mol Biol Evol 2011 in press 10.1093/molbev/msr121 PMC3203626 21546353

[B81] Wang Y Geer LY Chappey C Kans JA Bryant SH Cn3D: sequence and structure views for Entrez Trends Biochem Sci 2000 25 300 302 10.1016/S0968-0004(00)01561-9 10838572

[B82] Kang JY Nan X Jin MS Youn SJ Ryu YH Mah S Han SH Lee H Paik SG Lee JO Recognition of lipopeptide patterns by Toll-like receptor 2-Toll-like receptor 6 heterodimer Immunity 2009 31 873 884 10.1016/j.immuni.2009.09.018 19931471

