# Supplementary material for: Signatures of positive selection in Toll-like receptor (TLR) genes in mammals
Source: BMC Evol Biol. 2011 Dec 20;11:368. doi: 10.1186/1471-2148-11-368 (PMC3276489; doi:10.1186/1471-2148-11-368)
Supplement: Additional file 1 — Table S1. Identification of the sequences used for the TLR1 alignment. Microsoft Word document containing the list of accession numbers of the sequences used for the TLR1 alignment. [file 1471-2148-11-368-S1.DOC]

**Table S1. Identification of the sequences used for the TLR1 alignment**.

| **Species** | **TLR1** |
| --- | --- |
| *Ailuropoda melanoleuca* | XP_002928172.1 |
| *Callithrix jacchus* | XM_002745914.1 |
| *Canis lupus familiaris* | NM_001146143.1 |
| *Cavia porcellus* | ENSCPOT00000012321 |
| *Equus caballus* | XM_001498644.1 |
| *Erinaceus europaeus* | ENSEEUT00000015863 |
| *Homo sapiens* | NM_003263.3 |
| *Macaca mulatta* | NM_001130424.1 |
| *Mus musculus* | NM_030682.1 |
| *Oryctolagus cuniculus* | ENSOCUT00000001824 |
| *Pan troglodytes* | NM_001130465.1 |
| *Pongo abelii* | XM_002814670.1 |
| *Pongo pygmaeus* | ENSPPYT00000017054 |
| *Rattus norvegicus* | NM_001172120.1 |
| *Sus scrofa* | NM_001031775.1 |
| *Tarsius syrichta* | ENSTSYT00000005518 |
| *Tursiops truncatus* | ENSTTRT00000004025 |
