# Supplementary material for: Signatures of positive selection in Toll-like receptor (TLR) genes in mammals
Source: BMC Evol Biol. 2011 Dec 20;11:368. doi: 10.1186/1471-2148-11-368 (PMC3276489; doi:10.1186/1471-2148-11-368)
Supplement: Additional file 2 — Table S2. Identification of the sequences used for the TLR2 alignment. Microsoft Word document containing the list of accession numbers of the sequences used for the TLR2 alignment. [file 1471-2148-11-368-S2.DOC]

**Table S2. Identification of the sequences used for the TLR2 alignment**.

| **Species** | **TLR2** |
| --- | --- |
| *Bos taurus* | NM_174197.2 |
| *Callithrix jacchus* | ENSCJAT00000042796 |
| *Canis lupus familiaris* | NM_001005264.2 |
| *Cavia porcellus* | ENSCPOT00000025766 |
| *Equus caballus* | NM_001081796.1 |
| *Gorilla gorilla* | ENSGGOT00000003080 |
| *Homo sapiens* | NM_003264.3 |
| *Loxodonta africana* | ENSLAFT00000005070 |
| *Macaca mulatta* | NM_001130425.1 |
| *Microcebus murinus* | ENSMICT00000003193 |
| *Monodelphis domestica* | ENSMODT00000037954 |
| *Mus musculus* | NM_011905.3 |
| *Ochotona princeps* | ENSOPRT00000008718 |
| *Oryctolagus cuniculus* | NM_001082781.1 |
| *Ovis aries* | NM_001048231.1 |
| *Pan troglodytes* | NM_001130469.1 |
| *Pongo abelii* | XM_002815224.1 |
| *Pongo pygmaeus* | ENSPPYT00000017592 |
| *Rattus norvegicus* | NM_198769.2 |
| *Sus scrofa* | NM_213761.1 |
| *Tarsius syrichta* | ENSTSYT00000011049 |
| *Tursiops truncatus* | ENSTTRT00000004526 |
| *Vicugna pacos* | ENSVPAT00000006391 |
