# Supplementary material for: Signatures of positive selection in Toll-like receptor (TLR) genes in mammals
Source: BMC Evol Biol. 2011 Dec 20;11:368. doi: 10.1186/1471-2148-11-368 (PMC3276489; doi:10.1186/1471-2148-11-368)
Supplement: Additional file 3 — Table S3. Identification of the sequences used for the TLR3 alignment. Microsoft Word document containing the list of accession numbers of the sequences used for the TLR3 alignment. [file 1471-2148-11-368-S3.DOC]

**Table S3. Identification of the sequences used for the TLR3 alignment**.

| **Species** | **TLR3** |
| --- | --- |
| *Bos taurus* | NM_001008664.1 |
| *Canis lupus familiaris* | XM_540020.2 |
| *Cavia porcellus* | NM_001173029.1 |
| *Equus caballus* | NM_001081798.1 |
| *Erinaceus europaeus* | ENSEEUG00000008588 |
| *Felis catus* | NM_001079829.1 |
| *Homo sapiens* | NM_003265.2 |
| *Macaca mulatta* | NM_001036685.1 |
| *Mus musculus* | NM_126166.4 |
| *Oryctolagus cuniculus* | NM_001082219.1 |
| *Ovis aries* | NM_001135928.1 |
| *Pan troglodytes* | NM_001130470.1 |
| *Pongo abelii* | XM_002815350.1 |
| *Pongo pygmaeus* | ENSPPYT00000017734 |
| *Pteropus vampyrus* | ENSPVAG00000005843 |
| *Rattus norvegicus* | NM_198791.1 |
| *Sorex araneus* | ENSSARG00000011830 |
| *Sus scrofa* | NM_001097444.1 |
| *Tarsius syrichta* | ENSTSYG00000009003 |
| *Tursiops truncatus* | ENSTTRG00000015619 |
