# Supplementary material for: Signatures of positive selection in Toll-like receptor (TLR) genes in mammals
Source: BMC Evol Biol. 2011 Dec 20;11:368. doi: 10.1186/1471-2148-11-368 (PMC3276489; doi:10.1186/1471-2148-11-368)
Supplement: Additional file 4 — Table S4. Identification of the sequences used for the TLR4 alignment. Microsoft Word document containing the list of accession numbers of the sequences used for the TLR4 alignment. [file 1471-2148-11-368-S4.DOC]

**Table S4. Identification of the sequences used for the TLR4 alignment**.

| **Species** | **TLR4** |
| --- | --- |
| *Bos taurus* | NM_174198.6 |
| *Canis lupus familiaris* | NM_001002950.2 |
| *Dipodomys ordii* | ENSDORT00000001288 |
| *Equus caballus* | NM_001099769.1 |
| *Felis catus* | NM_001009223.1 |
| *Homo sapiens* | NM_138554.3 |
| *Loxodonta africana* | ENSLAFT00000006774 |
| *Macaca mulatta* | NM_001037092.1 |
| *Microcebus murinus* | ENSMICT00000004341 |
| *Monodelphis domestica* | XM_001368732.1 |
| *Mus musculus* | NM_021297.2 |
| *Myotis lucifugus* | ENSMLUT00000007409 |
| *Oryctolagus cuniculus* | NM_001082732.1 |
| *Ovis aries* | [NM_001135930.1](http://www.ncbi.nlm.nih.gov/nuccore/NM_001135930.1) |
| *Pan troglodytes* | NM_001144863.1 |
| *Pteropus vampyrus* | ENSPVAT00000014087 |
| *Rattus norvegicus* | NM_019178.1 |
| *Sus scrofa* | NM_001113039.1 |
| *Tarsius syrichta* | ENSTSYT00000003579 |
