# Supplementary material for: Signatures of positive selection in Toll-like receptor (TLR) genes in mammals
Source: BMC Evol Biol. 2011 Dec 20;11:368. doi: 10.1186/1471-2148-11-368 (PMC3276489; doi:10.1186/1471-2148-11-368)
Supplement: Additional file 5 — Table S5. Identification of the sequences used for the TLR5 alignment. Microsoft Word document containing the list of accession numbers of the sequences used for the TLR5 alignment. [file 1471-2148-11-368-S5.DOC]

**Table S5. Identification of the sequences used for the TLR5 alignment**.

| **Species** | **TLR5** |
| --- | --- |
| *Ailuropoda melanoleuca* | NW_003217862.1 |
| *Bos taurus* | NM_001040501.1 |
| *Callithrix jacchus* | XM_002760480.1 |
| *Canis lupus familiaris* | NM_001197176.1 |
| *Echinops telfairi* | ENSETET00000013405 |
| *Gallus gallus* | NM_001024586.1 |
| *Homo sapiens* | NM_003268.5 |
| *Macaca mulatta* | NM_001130429.1 |
| *Monodelphis domestica* | XM_001376152.1 |
| *Mus musculus* | NM_016928.2 |
| *Ochotona princeps* | ENSOPRT00000013962 |
| *Ornithorhynchus anatinus* | NW_001794625.1 |
| *Ovis aries* | NM_001135926.1 |
| *Pan troglodytes* | NM_001130462.1 |
| *Rattus norvegicus* | NM_001145828.1 |
| *Sus scrofa* | NM_001123202.1 |
| *Tarsius syrichta* | ENSTSYT00000011896 |
