# Supplementary material for: Signatures of positive selection in Toll-like receptor (TLR) genes in mammals
Source: BMC Evol Biol. 2011 Dec 20;11:368. doi: 10.1186/1471-2148-11-368 (PMC3276489; doi:10.1186/1471-2148-11-368)
Supplement: Additional file 6 — Table S6. Identification of the sequences used for the TLR6 alignment. Microsoft Word document containing the list of accession numbers of the sequences used for the TLR6 alignment. [file 1471-2148-11-368-S6.DOC]

**Table S6. Identification of the sequences used for the TLR6 alignment**.

| **Species** | **TLR6** |
| --- | --- |
| *Ailuropoda melanoleuca* | *NW_003218729.1* |
| *Bos taurus* | *NM_001001159.1* |
| *Callithrix jacchus* | *XM_002745913.1* |
| *Cavia porcellus* | *ENSCPOT00000005143* |
| *Equus caballus* | *XM_001498630.1* |
| *Erinaceus europaeus* | *ENSEEUT00000015873* |
| *Homo sapiens* | *NM_006068.3* |
| *Loxodonta africana* | *ENSLAFT00000026325* |
| *Macaca mulatta* | *NM_001130430.1* |
| *Microcebus murinus* | *ENSMICT00000007898* |
| *Mus musculus* | *NM_011604.3* |
| *Oryctolagus cuniculus* | *XM_002709388.1* |
| *Otolemur garnettii* | *ENSOGAT00000016500* |
| *Ovis aries* | *NM_001135927.1* |
| *Pan troglodytes* | *NM_001130468.1* |
| *Pongo abelii* | *XM_002814667.1* |
| *Pongo pygmaeus* | *ENSPPYT00000017055* |
| *Rattus norvegicus* | *NM_207604.1* |
| *Sorex araneus* | *ENSSART00000000729* |
| *Sus scrofa* | *NM_213760.1* |
