# Supplementary material for: Signatures of positive selection in Toll-like receptor (TLR) genes in mammals
Source: BMC Evol Biol. 2011 Dec 20;11:368. doi: 10.1186/1471-2148-11-368 (PMC3276489; doi:10.1186/1471-2148-11-368)
Supplement: Additional file 7 — Table S7. Identification of the sequences used for the TLR7 alignment. Microsoft Word document containing the list of accession numbers of the sequences used for the TLR7 alignment. [file 1471-2148-11-368-S7.DOC]

**Table S7. Identification of the sequences used for the TLR7 alignment**.

| **Species** | **TLR7** |
| --- | --- |
| *Bos taurus* | NM_001033761 |
| *Callithrix jacchus* | [XM_002762618.1](http://www.ncbi.nlm.nih.gov/nuccore/XM_002762618.1) |
| *Canis lupus familiaris* | NM_001048124 |
| *Cercocebus torquatus* | EU204942 |
| *Equus caballus* | NM_001081771 |
| *Felis catus* | NM_001080133 |
| *Gorilla gorilla* | AB445662_mRNA |
| *Homo sapiens* | [NM_016562.3](http://www.ncbi.nlm.nih.gov/nuccore/NM_016562.3) |
| *Hylobates lar* | EU488853 |
| *Loxodonta africana* | DQ360413 |
| *Macaca mulatta* | NM_001130426_mRNA |
| *Mus musculus* | NM_133211 |
| *Ovis aries* | NM_001135059 |
| *Pan troglodytes* | NM_001130133_mRNA |
| *Pongo pygmaeus* | AB445663_mRNA |
| *Pteropus vampyrus* | ENSPVAT00000009452 |
| *Rattus norvegicus* | NM_001097582 |
| *Rousettus leschenaultii* | AB472356 |
| *Saguinus oedipus* | EU488862 |
| *Sus scrofa* | NM_001097434 |
| *Tursiops truncatus* | ENSTTRT00000003227 |
