# Supplementary material for: Signatures of positive selection in Toll-like receptor (TLR) genes in mammals
Source: BMC Evol Biol. 2011 Dec 20;11:368. doi: 10.1186/1471-2148-11-368 (PMC3276489; doi:10.1186/1471-2148-11-368)
Supplement: Additional file 8 — Table S8. Identification of the sequences used for the TLR8 alignment. Microsoft Word document containing the list of accession numbers of the sequences used for the TLR8 alignment. [file 1471-2148-11-368-S8.DOC]

**Table S8. Identification of the sequences used for the TLR8 alignment**.

| **Species** | **TLR8** |
| --- | --- |
| *Canis lupus familiaris* | ENSCAFT00000036159 |
| *Cavia porcellus* | ENSCPOT00000004812 |
| *Choloepus hoffmanni* | ENSCHOT00000008493 |
| *Equus caballus* | ENSECAT00000009327 |
| *Erinaceus europaeus* | ENSEEUT00000006688 |
| *Homo sapiens* | NM_138636.4 |
| *Macaca mulatta* | NM_001130427.1 |
| *Microcebus murinus* | ENSMICT00000014444 |
| *Monodelphis domestica* | ENSMODT00000022002 |
| *Mus musculus* | NM_133212.2 |
| *Ovis aries* | NM_001135929.1 |
| *Pan troglodytes* | NM_001130472.1 |
| *Pongo abelii* | XM_002831381.1 |
| *Pongo pygmaeus* | ENSPPYT00000023470 |
| *Rattus norvegicus* | NM_001101009.1 |
| *Sus scrofa* | NM_214187.1 |
| *Tursiops truncatus* | ENSTTRT00000003228 |
| *Vicugna pacos* | ENSVPAT00000000326 |
