# Supplementary material for: Signatures of positive selection in Toll-like receptor (TLR) genes in mammals
Source: BMC Evol Biol. 2011 Dec 20;11:368. doi: 10.1186/1471-2148-11-368 (PMC3276489; doi:10.1186/1471-2148-11-368)
Supplement: Additional file 9 — Table S9. Identification of the sequences used for the TLR9 alignment. Microsoft Word document containing the list of accession numbers of the sequences used for the TLR9 alignment. [file 1471-2148-11-368-S9.DOC]

**Table S9. Identification of the sequences used for the TLR9 alignment**.

| **Species** | **TLR9** |
| --- | --- |
| *Aotus nancymaae* | AY788894 |
| *Bos taurus* | NM_183081.1 |
| *Bos indicus* | EF076730 |
| *Boselaphus tragocamelus* | EU747828 |
| *Bubalus bubalis* | FJ606787 |
| *Canis lupus familiaris* | AY859723 |
| *Capra hircus* | EU747825 |
| *Cercocebus torquatus* | EU204946 |
| *Dipodomys ordii* | ENSDORT00000012817 |
| *Equus caballus* | NM_001081790 |
| *Felis catus* | AY859724 |
| *Gorilla gorilla* | AB445676 |
| *Homo sapiens* | EU170542 |
| *Macaca fascicularis* | AB445678 |
| *Mus musculus* | NM_031178 |
| *Ovis aries* | NM_001011555 |
| *Pan paniscus* | AB445675 |
| *Pan troglodytes* | NM_001144866 |
| *Pongo pygmaeus* | AB445677 |
| *Rousettus leschenaultii* | AB472357 |
| *Sus scrofa* | AY859728 |
