# Supplementary material for: Signatures of positive selection in Toll-like receptor (TLR) genes in mammals
Source: BMC Evol Biol. 2011 Dec 20;11:368. doi: 10.1186/1471-2148-11-368 (PMC3276489; doi:10.1186/1471-2148-11-368)
Supplement: Additional file 10 — Table S10. Identification of the sequences used for the TLR10 alignment. Microsoft Word document containing the list of accession numbers of the sequences used for the TLR10 alignment. [file 1471-2148-11-368-S10.DOC]

**Table S10. Identification of the sequences used for the TLR10 alignment**.

| **Species** | **TLR10** |
| --- | --- |
| *Ailuropoda melanoleuca* | NW_003218729.1 |
| *Bos taurus* | NM_001076918.1 |
| *Callithrix jacchus* | XM_002745915.1 |
| *Canis lupus familiaris* | NM_001173127.1 |
| *Dasypus novemcinctus* | ENSDNOT00000009941 |
| *Homo sapiens* | NM_001017388.2 |
| *Loxodonta africana* | ENSLAFT00000008544 |
| *Macaca mulatta* | NM_001130434.1 |
| *Oryctolagus cuniculus* | ENSOCUT00000003699 |
| *Pan troglodytes* | NM_001130464.1 |
| *Pongo abelii* | XM_002814663.1 |
| *Rattus norvegicus* | NM_001146035.1 |
| *Sus scrofa* | NM_001030534.1 |
