# Supplementary material for: Signatures of positive selection in Toll-like receptor (TLR) genes in mammals
Source: BMC Evol Biol. 2011 Dec 20;11:368. doi: 10.1186/1471-2148-11-368 (PMC3276489; doi:10.1186/1471-2148-11-368)
Supplement: Additional file 11 — Table S11. Amino acid alterations found in TLR1 for each species at each positively selected site. Microsoft Word document containing the amino acid alterations at each site under selection in TLR1 gene. [file 1471-2148-11-368-S11.DOC]

Tabela S11. Amino acid alterations found in TLR1 for each species at each positively selected site.

Dots (.) indicate identity with the human sequence. Amino acid positions are according to the human sequence.

| **Species** | **Amino acid position and location** | |
| --- | --- | --- |
|  | **LRR6** | **LRR10** |
|  | **174** | **293** |
| ***Homo sapiens*** | **T** | **D** |
| *Pongo pygmaeus* | A | N |
| *Pongo abelii* | A | N |
| *Pan troglodytes* | . | . |
| *Macaca mulatta* | H | . |
| *Callithrix jacchus* | P | N |
| *Cavia porcellus* | S | G |
| *Oryctolagus cuniculus* | H | N |
| *Tarsius syrichta* | F | N |
| *Canis lúpus familiaris* | . | . |
| *Ailuropoda melanoleuca* | . | . |
| *Equus caballus* | P | . |
| *Erinaceus europaeus* | S | N |
| *Tursiops truncatus* | . | . |
| *Sus scrofa* | S | . |
| *Mus musculus* | A | N |
| *Rattus norvegicus* | . | H |
