# Supplementary material for: Signatures of positive selection in Toll-like receptor (TLR) genes in mammals
Source: BMC Evol Biol. 2011 Dec 20;11:368. doi: 10.1186/1471-2148-11-368 (PMC3276489; doi:10.1186/1471-2148-11-368)
Supplement: Additional file 12 — Table S12. Amino acid alterations found in TLR2 for each species at each positively selected site. Microsoft Word document containing the amino acid alterations at each site under selection in TLR2 gene. [file 1471-2148-11-368-S12.DOC]

Tabela S12. Amino acid alterations found in TLR2 for each species at each positively selected site.

Dots (.) indicate identity with the human sequence and (-) indicates a gap. Amino acid positions are according to the human sequence.

| **Species** | **Amino acid position and location** | | | | | |
| --- | --- | --- | --- | --- | --- | --- |
|  | **Signal** | **LRR5** | **LRR6** | **LRR10** | **Transmembrane** | **TIR** |
|  | **3** | **161** | **182** | **302** | **602** | **636** |
| ***Homo sapiens*** | **H** | **T** | **D** | **R** | **I** | **S** |
| *Pan troglodytes* | . | . | . | . | . | . |
| *Pongo abelii* | . | . | . | . | . | . |
| *Macaca mulatta* | . | . | . | . | . | N |
| *Gorilla gorilla* | . | . | . | . | . | . |
| *Rattus norvegicus* | Q | . | Q | V | . | C |
| *Mus musculus* | R | . | K | V | . | C |
| *Bos taurus* | R | S | S | . | L | R |
| *Sus scrofa* | C | . | G | T | L | R |
| *Canis lupus familiaris* | R | S | . | K | V | . |
| *Tursiops truncatus* | P | S | N | T | F | R |
| *Vicugna pacos* | . | P | . | T | L | R |
| *Ovis aries* | R | S | S | . | L | R |
| *Oryctolagus cuniculus* | P | A | . | L | T | C |
| *Microcebus murinus* | Y | S | . | I | . | R |
| *Tarsius syrichta* | . | . | E | . | . | G |
| *Callithrix jacchus* | . | . | . | . | V | . |
| *Pongo pygmaeus* | . | . | . | . | . | . |
| *Ochotona princeps* | L | . | . | T | M | . |
| *Equus caballus* | . | H | . | K | . | Q |
| *Cavia porcellus* | . | H | E | - | L | R |
| *Loxodonta africana* | . | Y | . | K | . | N |
| *Monodelphis domestica* | Q | R | E | E | A | D |
