# Supplementary material for: Signatures of positive selection in Toll-like receptor (TLR) genes in mammals
Source: BMC Evol Biol. 2011 Dec 20;11:368. doi: 10.1186/1471-2148-11-368 (PMC3276489; doi:10.1186/1471-2148-11-368)
Supplement: Additional file 13 — Table S13. Amino acid alterations found in TLR3 for each species at each positively selected site. Microsoft Word document containing the amino acid alterations at each site under selection in TLR3 gene. [file 1471-2148-11-368-S13.DOC]

Tabela S13. Amino acid alterations found in TLR3 for each species at each positively selected site.

**Dots (.) indicate identity with the human sequence. Amino acid positions are according to the human sequence.**

| **Species** | **Amino acid position and location** | | | | | | | | |
| --- | --- | --- | --- | --- | --- | --- | --- | --- | --- |
|  | **Signal** | | | **LRR2** | **LRR9** | **LRR10** | **Transmembrane** | **TIR** | |
|  | **4** | **12** | **25** | **79** | **258** | **285** | **712** | **749** | **780** |
| ***Homo sapiens*** | **T** | **G** | **T** | **S** | **S** | **N** | **I** | **R** | **E** |
| *Bos taurus* | P | S | A | T | N | S | . | . | . |
| *Canis familiaris* | S | L | . | I | T | . | V | K | K |
| *Cavia porcellus* | P | L | N | V | T | . | V | G | K |
| *Equus caballus* | S | L | C | I | T | . | F | S | Q |
| *Erinaceus europaeus* | Y | W | S | I | V | K | F | . | Q |
| *Felis catus* | S | L | . | T | T | . | . | . | K |
| *Macaca mulatta* | . | W | . | I | . | . | . | . | . |
| *Mus musculus* | C | . | . | I | N | . | M | T | Q |
| *Oryctolagus cuniculus* | S | W | G | V | . | . | F | G | . |
| *Ovis aries* | P | S | A | I | N | S | . | K | . |
| *Pan troglodytes* | . | . | . | . | . | . | . | . | . |
| *Pongo abelii* | . | . | . | T | . | . | . | . | . |
| *Pongo pygmaeus* | . | . | . | T | . | . | . | . | . |
| *Pteropus vampyrus* | . | F | . | V | T | G | . | S | . |
| *Rattus norvegicus* | R | . | . | L | N | S | T | A | Q |
| *Sorex araneus* | . | W | V | V | V | S | . | K | K |
| *Sus scrofa* | S | W | . | T | I | S | M | K | K |
| *Tarsius syrichta* | N | . | A | I | I | S | . | K | K |
| *Tursiops truncatus* | D | L | . | I | N | S | . | . | . |
