# Supplementary material for: Signatures of positive selection in Toll-like receptor (TLR) genes in mammals
Source: BMC Evol Biol. 2011 Dec 20;11:368. doi: 10.1186/1471-2148-11-368 (PMC3276489; doi:10.1186/1471-2148-11-368)
Supplement: Additional file 14 — Table S14. Amino acid alterations found in TLR4 for each species at each positively selected site. Microsoft Word document containing the amino acid alterations at each site under selection in TLR4 gene. [file 1471-2148-11-368-S14.DOC]

Tabela S14. Amino acid alterations found in TLR4 for each species at each positively selected site.

Dots (.) indicate identity with the human sequence and (-) indicates a gap. Amino acid positions are according to the human sequence.

| **Species** | **Amino acid position and location** | | | | | | | | | | | | | | | | | | | | | |
| --- | --- | --- | --- | --- | --- | --- | --- | --- | --- | --- | --- | --- | --- | --- | --- | --- | --- | --- | --- | --- | --- | --- |
|  | **LRR6** | **LRR8** | **LRR9** | | **LRR10** | | | **LRR11** | | **LRR13** | | | **LRR14** | | **LRR17** | | **LRR18** | **LRR19** | **LRR20** | **LRR-CT** | **Transmembrane** | **TIR** |
|  | **204** | **240** | **270** | **276** | **295** | **300** | **301** | **317** | **319** | **356** | **363** | **370** | **382** | **394** | **468** | **471** | **487** | **500** | **542** | **604** | **639** | **673** |
| ***Homo sapiens*** | **L** | **L** | **E** | **A** | **Y** | **I** | **I** | **S** | **T** | **L** | **G** | **V** | **R** | **S** | **N** | **S** | **F** | **F** | **C** | **V** | **L** | **I** |
| *Bos taurus* | . | S | Q | F | K | D | T | . | S | F | D | F | . | T | T | V | L | V | P | A | V | T |
| *Canis lupus familiaris* | . | T | . | L | S | T | T | H | Y | F | . | M | . | . | D | V | S | I | P | . | F | T |
| *Equus caballus* | . | I | . | . | . | S | . | . | D | . | D | . | . | A | D | V | . | T | P | . | F | . |
| *Felis catus* | . | T | G | I | K | A | . | H | Y | . | V | . | . | R | . | I | . | I | P | . | F | T |
| *Dipodomys ordii* | . | T | D | . | E | V | T | N | Y | . | . | I | . | E | D | T | T | I | H | A | . | A |
| *Loxodonta africana* | . | M | D | . | D | D | . | . | N | F | N | F | G | . | L | Y | . | . | Y | . | . | T |
| *Macaca mulatta* | S | . | . | S | . | . | . | . | S | . | . | . | . | . | D | L | . | . | . | A | F | . |
| *Microcebus murinus* | . | M | N | . | E | A | - | . | G | F | D | . | K | D | A | V | V | . | S | A | V | T |
| *Myotis lucifugus* | F | A | V | . | E | D | L | G | Y | F | . | . | K | R | D | I | L | E | N | A | . | T |
| *Oryctolagus cuniculus* | G | T | K | . | D | . | T | H | Y | . | . | L | N | N | D | Y | R | T | . | . | F | V |
| *Ovis aries* | . | S | Q | C | K | D | T | . | S | F | D | T | . | T | T | V | L | I | P | A | V | T |
| *Pan troglodytes* | . | . | . | . | . | . | . | . | . | . | . | . | . | . | . | . | . | . | . | . | . | . |
| *Mus musculus* | V | S | . | I | D | . | V | G | S | . | . | . | . | . | L | T | T | . | Q | . | V | . |
| *Rattus norvegicus* | V | S | . | V | H | . | Y | G | H | W | E | L | . | . | L | I | T | . | Q | . | V | . |
| *Pteropus vampyrus* | F | T | N | . | D | V | . | . | Y | . | . | . | . | T | D | F | S | . | L | . | . | T |
| *Sus scrofa* | . | S | . | V | E | V | S | . | N | F | H | I | G | N | A | V | L | L | P | A | . | T |
| *Tarsius syrichta* | I | M | . | V | H | A | T | H | N | F | S | F | . | T | . | . | L | . | H | . | . | T |
| *Monodelphis domestica* | - | S | T | I | Q | G | N | N | D | . | D | L | K | K | T | . | I | S | P | S | . | . |
