# Supplementary material for: Signatures of positive selection in Toll-like receptor (TLR) genes in mammals
Source: BMC Evol Biol. 2011 Dec 20;11:368. doi: 10.1186/1471-2148-11-368 (PMC3276489; doi:10.1186/1471-2148-11-368)
Supplement: Additional file 15 — Table S15. Amino acid alterations found in TLR5 for each species at each positively selected site. Microsoft Word document containing the amino acid alterations at each site under selection in TLR5 gene. [file 1471-2148-11-368-S15.DOC]

Tabela S15. Amino acid alterations found in TLR5 for each species at each positively selected site.

Dots (.) indicate identity with the human sequence. Amino acid positions are according to the human sequence.

| **Species** | **Amino acid position and location** | | | | | | |
| --- | --- | --- | --- | --- | --- | --- | --- |
|  | **Signal** | **LRR4** | **LRR7** | **LRR14** | **TIR** | | |
|  | **14** | **128** | **207** | **400** | **674** | **721** | **742** |
| ***Homo sapiens*** | **M** | **F** | **S** | **H** | **A** | **D** | **A** |
| *Pan troglodytes* | . | . | . | . | . | . | . |
| *Macaca mulatta* | V | . | N | . | . | . | . |
| *Bos taurus* | L | S | Q | Y | . | S | . |
| *Tarsius syrichta* | . | A | . | N | V | T | . |
| *Ovis aries* | L | . | H | . | . | N | . |
| *Ornithorhynchus anatinus* | G | Y | Q | . | V | . | S |
| *Ailuropoda melanoleuca* | A | Y | H | S | . | . | . |
| *Echinops telfairi* | V | Y | N | . | . | . | V |
| *Canis lupus familiaris* | V | A | G | S | . | S | . |
| *Ochotona princeps* | . | G | N | S | . | . | T |
| *Mus musculus* | . | S | K | G | I | S | S |
| *Rattus norvegicus* | . | D | . | G | I | S | S |
| *Sus scrofa* | V | S | N | Q | V | . | S |
| *Monodelphis domestica* | K | Y | N | Y | F | P | N |
| *Callithrix jacchus* | V | . | . | Y | . | . | . |
