# Supplementary material for: Signatures of positive selection in Toll-like receptor (TLR) genes in mammals
Source: BMC Evol Biol. 2011 Dec 20;11:368. doi: 10.1186/1471-2148-11-368 (PMC3276489; doi:10.1186/1471-2148-11-368)
Supplement: Additional file 16 — Table S16. Amino acid alterations found in TLR6 for each species at each positively selected site. Microsoft Word document containing the amino acid alterations at each site under selection in TLR6 gene. [file 1471-2148-11-368-S16.DOC]

Tabela S16. Amino acid alterations found in TLR6 for each species at each positively selected site.

Dots (.) indicate identity with the human sequence and (-) indicates a gap. Amino acid positions are according to the human sequence.

| **Species** | **Amino acid position and location** | | |
| --- | --- | --- | --- |
|  | **Transmembrane** | | **TIR** |
|  | **604** | **607** | **796** |
| ***Homo sapiens*** | **S** | **I** | **S** |
| *Pongo abelii* | . | . | . |
| *Pan troglodytes* | . | . | . |
| *Macaca mulatta* | F | . | . |
| *Otolemur garnettii* | F | . | I |
| *Pongo pygmaeus* | . | . | . |
| *Callithrix jacchus* | . | . | . |
| *Sus scrofa* | G | V | T |
| *Ovis aries* | V | . | G |
| *Equus caballus* | I | . | T |
| *Bos taurus* | V | . | - |
| *Ailuropoda melanoleuca* | A | . | T |
| *Microcebus murinus* | F | . | T |
| *Mus musculus* | F | L | - |
| *Rattus norvegicus* | . | L | - |
| *Oryctolagus cuniculus* | C | V | T |
| *Cavia porcellus* | F | C | L |
| *Erinaceus europaeus* | M | . | - |
| *Loxodonta africana* | F | M | T |
| *Sorex araneus* | I | V | . |
