# Supplementary material for: Signatures of positive selection in Toll-like receptor (TLR) genes in mammals
Source: BMC Evol Biol. 2011 Dec 20;11:368. doi: 10.1186/1471-2148-11-368 (PMC3276489; doi:10.1186/1471-2148-11-368)
Supplement: Additional file 17 — Table S17. Amino acid alterations found in TLR7 for each species at each positively selected site. Microsoft Word document containing the amino acid alterations at each site under selection in TLR7 gene. [file 1471-2148-11-368-S17.DOC]

Tabela S17. Amino acid alterations found in TLR7 for each species at each positively selected site.

Dots (.) indicate identity with the human sequence. Amino acid positions are according to the human sequence.

| **Species** | **Amino acid position and location** | | | | | | |
| --- | --- | --- | --- | --- | --- | --- | --- |
|  | **LRR8** | **LRR11** | **LRR12** | **LRR19** | **LRR21** | **LRR22** | **LRR26** |
|  | **283** | **359** | **386** | **599** | **667** | **697** | **776** |
| ***Homo Sapiens*** | **V** | **S** | **K** | **Q** | **S** | **C** | **K** |
| *Macaca mulatta* | . | . | . | . | . | Y | . |
| *Pongo pygmaeus* | . | . | . | . | . | . | . |
| *Gorilla gorilla* | . | . | . | . | . | . | . |
| *Pan troglodytes* | . | . | . | . | . | . | . |
| *Saguinus oedipus* | . | . | . | . | . | S | . |
| *Hylobates lar* | . | . | . | . | . | S | . |
| *Cercocebus torquatus* | . | . | . | . | . | Y | . |
| *Equus caballus* | A | . | . | R | P | L | D |
| *Felis catus* | M | T | S | K | . | Y | E |
| *Canis lupus* | E | A | S | K | . | Y | E |
| *Bos taurus* | P | V | N | R | L | S | N |
| *Ovis aries* | L | V | N | K | L | S | N |
| *Sus scrofa* | L | F | . | K | . | Y | Q |
| *Loxodonta africana* | . | H | . | K | P | V | E |
| *Rousettus leschenaultii* | E | F | N | R | P | Y | . |
| *Mus musculus* | D | . | . | D | P | L | E |
| *Rattus norvegicus* | D | . | . | E | P | L | N |
| *Callithrix jacchus* | E | F | Q | . | P | . | E |
| *Tursiops truncatus* | L | F | R | K | . | Y | N |
| *Pteropus vampyrus* | E | F | N | R | P | Y | . |
