# Supplementary material for: Signatures of positive selection in Toll-like receptor (TLR) genes in mammals
Source: BMC Evol Biol. 2011 Dec 20;11:368. doi: 10.1186/1471-2148-11-368 (PMC3276489; doi:10.1186/1471-2148-11-368)
Supplement: Additional file 18 — Table S18. Amino acid alterations found in TLR8 for each species at each positively selected site. Microsoft Word document containing the amino acid alterations at each site under selection in TLR8 gene. [file 1471-2148-11-368-S18.DOC]

Tabela S18. Amino acid alterations found in TLR8 for each species at each positively selected site.

Dots (.) indicate identity with the human sequence and (-) indicates a gap. Amino acid positions are according to the human sequence.

| **Species** | **Amino acid position and location** | | | | | | | | | | | | | | | | | | | | | | | | | |
| --- | --- | --- | --- | --- | --- | --- | --- | --- | --- | --- | --- | --- | --- | --- | --- | --- | --- | --- | --- | --- | --- | --- | --- | --- | --- | --- |
|  | **LRR-NT** | **LRR3** | **LRR4** | **LRR5** | | | **LRR6** | **LRR7** | **LRR8** | **LRR10** | **LRR11** | **LRR12** | **LRR13** | | **Undefined region (LRR14-LRR15)** | | | | **LRR15** | **LRR19** | **LRR20** | | | **LRR23** | **LRR25** | **LRR26** |
|  | **39** | **146** | **160** | **174** | **186** | **191** | **214** | **236** | **249** | **338** | **349** | **388** | **416** | **418** | **441** | **459** | **472** | **481** | **498** | **606** | **629** | **633** | **639** | **712** | **761** | **766** |
| ***Homo Sapiens*** | **K** | **E** | **N** | **N** | **V** | **N** | **S** | **Y** | **T** | **R** | **I** | **Q** | **N** | **S** | **Y** | **F** | **R** | **A** | **P** | **E** | **N** | **S** | **K** | **S** | **T** | **M** |
| *Canis lupus familiaris* | R | G | W | R | N | . | V | I | R | V | V | E | . | P | E | M | H | V | R | R | D | K | R | . | N | I |
| *Cavia porcellus* | S | . | L | K | N | E | I | A | S | N | V | E | . | . | S | S | Y | R | R | K | T | P | S | . | A | S |
| *Equus caballus* | . | G | S | Y | I | D | . | N | . | S | . | R | H | P | . | . | N | . | Q | N | R | K | R | P | . | V |
| *Macaca mulatta* | . | . | . | Y | . | . | . | . | . | . | . | . | . | P | . | . | . | . | . | . | . | . | T | . | . | I |
| *Mus musculus* | I | . | Q | R | K | K | F | N | . | S | Q | E | . | . | - | D | K | . | K | K | G | . | Q | H | K | I |
| *Ovis aries* | . | . | T | R | C | S | H | N | R | S | E | K | W | P | D | I | H | R | V | K | V | Q | T | . | A | I |
| *Pan troglodytes* | . | . | . | . | . | . | . | . | . | . | . | . | . | . | . | . | . | . | . | . | . | . | . | . | . | . |
| *Pongo abelii* | . | . | . | . | . | . | . | . | . | . | . | . | . | . | F | . | . | . | . | . | . | . | T | . | . | K |
| *Pongo aygmaeus* | . | . | . | . | . | . | . | . | . | . | . | . | . | . | F | . | . | . | . | . | . | . | T | . | . | K |
| *Sus acrofa* | R | . | L | S | C | I | Y | N | R | S | E | . | E | . | N | P | K | R | . | K | D | Q | S | T | . | I |
| *Monodelphis domestica* | R | P | S | S | G | S | L | N | R | N | L | . | D | R | - | G | . | S | S | . | . | K | S | . | S | I |
| *Rattus norvegicus* | . | . | Q | R | K | K | F | T | I | S | . | K | D | P | - | Y | E | T | K | Q | G | . | E | H | K | V |
| *Erinaceus europeus* | . | Q | R | T | S | V | H | T | E | . | R | . | E | . | D | E | N | . | K | S | . | E | . | P | S | V |
| *Choloepus hoffmanni* | R | R | L | R | H | D | V | N | K | S | V | E | H | P | F | S | N | . | K | K | D | K | S | N | A | I |
| *Vicugna pacus* | . | G | V | R | - | A | F | R | R | S | . | R | . | P | - | . | H | S | A | R | V | Q | S | . | A | I |
| *Tursiopus truncatus* | T | K | V | S | - | A | F | N | K | S | E | . | S | . | N | T | N | N | I | K | V | Q | S | . | A | I |
| *Microcebus murinus* | . | G | G | R | . | D | F | K | I | H | V | . | . | . | . | T | S | D | K | . | . | . | T | . | I | L |
