# Supplementary material for: Signatures of positive selection in Toll-like receptor (TLR) genes in mammals
Source: BMC Evol Biol. 2011 Dec 20;11:368. doi: 10.1186/1471-2148-11-368 (PMC3276489; doi:10.1186/1471-2148-11-368)
Supplement: Additional file 19 — Table S19. Amino acid alterations found in TLR9 for each species at each positively selected site. Microsoft Word document containing the amino acid alterations at each site under selection in TLR9 gene. [file 1471-2148-11-368-S19.DOC]

Tabela S19. Amino acid alterations found in TLR9 for each species at each positively selected site.

**Dots (.) indicate identity with the human sequence. Amino acid positions are according to the human sequence.**

| **Species** | **Amino acid position and location** | | | |
| --- | --- | --- | --- | --- |
|  | **LRR1** | **LRR4** | **LRR6** | **LRR10** |
|  | **71** | **161** | **217** | **332** |
| ***Homo sapiens*** | **S** | **A** | **N** | **G** |
| *Mus musculus* | I | N | Q | N |
| *Canis familiaris* | Y | . | G | . |
| *Felis catus* | Y | . | G | . |
| *Equus caballus* | L | T | S | . |
| *Sus scrofa* | L | T | S | . |
| *Bubalus bubalis* | I | T | H | D |
| *Bos indicus* | I | T | R | D |
| *Boselaphus tragocamelus* | I | C | C | N |
| *Capra hircus* | I | T | R | N |
| *Ovis aries* | I | T | R | N |
| *Rousettus leschenaultii* | R | . | R | N |
| *Pan paniscus* | . | . | . | . |
| *Pan troglodytes* | . | . | . | . |
| *Gorilla gorilla* | . | . | . | . |
| *Aotus nancymaae* | . | . | . | . |
| *Pongo pygmaeus* | . | . | . | . |
| *Cercocebus turquatus* | . | D | . | . |
| *Macaca fascicularis* | . | D | . | . |
| *Bos taurus* | I | T | R | D |
| *Dipodomys ordii* | L | S | F | G |
