# Supplementary material for: Signatures of positive selection in Toll-like receptor (TLR) genes in mammals
Source: BMC Evol Biol. 2011 Dec 20;11:368. doi: 10.1186/1471-2148-11-368 (PMC3276489; doi:10.1186/1471-2148-11-368)
Supplement: Additional file 20 — Table S20. Amino acid alterations found in TLR10 for each species at each positively selected site. Microsoft Word document containing the amino acid alterations at each site under selection in TLR10 gene. [file 1471-2148-11-368-S20.DOC]

Tabela S20. Amino acid alterations found in TLR10 for each species at each positively selected site.

Dots (.) indicate identity with the human sequence. Amino acid positions are according to the human sequence.

| **Species** | **Amino acid position and location** | | | | | | |
| --- | --- | --- | --- | --- | --- | --- | --- |
|  | **LRR2** | **LRR8** | **LRR14** | **LRR18** | **LRR19** | **LRR-CT** | **TIR** |
|  | **91** | **238** | **392** | **469** | **492** | **545** | **803** |
| ***Homo sapiens*** | **T** | **S** | **C** | **R** | **V** | **V** | **I** |
| *Rattus norvegicus* | I | . | L | . | S | S | . |
| *Bos taurus* | . | I | H | . | . | . | . |
| *Sus scrofa* | . | I | . | Q | . | . | . |
| *Pan troglodytes* | . | . | . | . | I | . | . |
| *Ailuropoda melanoleuca* | . | T | F | Q | I | T | V |
| *Canis lupus familiaris* | I | T | F | Q | I | I | . |
| *Callithrix jacchus* | I | . | R | Q | . | . | V |
| *Macaca mulatta* | . | . | . | . | I | . | V |
| *Pongo abelii* | . | . | . | . | I | . | . |
| *Dasypus novemcinctus* | V | I | S | Q | I | . | M |
| *Loxodonta africana* | A | R | H | . | I | A | D |
| *Oryctolagus cuniculus* | S | I | . | . | . | L | S |
