# Supplementary material for: Signatures of positive selection in Toll-like receptor (TLR) genes in mammals
Source: BMC Evol Biol. 2011 Dec 20;11:368. doi: 10.1186/1471-2148-11-368 (PMC3276489; doi:10.1186/1471-2148-11-368)
Supplement: Additional file 21 — Table S21. Domain characterization of TLR1. Microsoft Word document containing the list of domains of Human TLR1 gene, their delimitation and sequence. [file 1471-2148-11-368-S21.DOC]

Table S21. Domain characterization of TLR1.

**The conserved segment of each LRR is underlined. The amino acids identified as under positive selection are in bold.**

| **TLR1 – *Homo sapiens*** | | | |
| --- | --- | --- | --- |
| **Domain** | **Start** | **Stop** | **Sequence** |
| **Signal** | 1 | 21 | MTSIFHFAIIFMLILQIRIQL |
| [**LRR**](http://smart.embl-heidelberg.de/smart/do_annotation.pl?DOMAIN=LRR&TYPE=SMART&START=51&END=70&LENGTH=19&E_VALUE=69.0126970495531&BLAST=PTNITVLNLTHNQIKRLPPA)**-NT** | 22 | 46 | SEESEFLVDRSKNGLIHVPKDLSQK |
| **LRR1** | 47 | 70 | TTILNISQNYISELWTSDILSLSK |
| [**LRR**](http://smart.embl-heidelberg.de/smart/do_annotation.pl?DOMAIN=LRR&TYPE=SMART&START=123&END=144&LENGTH=21&E_VALUE=289.551614689825&BLAST=CMNLTELHLMSNSIQKIQNNPF)**2** | 71 | 94 | LRILIISHNRIQYLDISVFKFNQE |
| [**LRR**](http://smart.embl-heidelberg.de/smart/do_annotation.pl?DOMAIN=LRR&TYPE=SMART&START=171&END=194&LENGTH=23&E_VALUE=57.8362009479994&BLAST=LQNLQELLLSKNKIQALKSEELAF)**3** | 95 | 115 | LEYLDLSHNKLVKISCHPTVN |
| [**LRR**](http://smart.embl-heidelberg.de/smart/do_annotation.pl?DOMAIN=LRR&TYPE=SMART&START=197&END=218&LENGTH=21&E_VALUE=384.070417219697&BLAST=NSSLKKLELSSNLIKEFSPGCF)**4** | 116 | 140 | LKHLDLSFNAFDALPICKEFGNMSQ |
| [**LRR**](http://smart.embl-heidelberg.de/smart/do_annotation.pl?DOMAIN=LRR&TYPE=SMART&START=197&END=218&LENGTH=21&E_VALUE=384.070417219697&BLAST=NSSLKKLELSSNLIKEFSPGCF)**5** | 141 | 163 | LKFLGLSTTHLEKSSVLPIAHLN |
| [**LRR**](http://smart.embl-heidelberg.de/smart/do_annotation.pl?DOMAIN=LRR&TYPE=SMART&START=274&END=295&LENGTH=21&E_VALUE=6.4745441770878&BLAST=HTNLTMLDLSHNNLNMIDDDSF)**6** | 164 | 188 | ISKVLLVLGE**T**YGEKEDPEGLQDFN |
| **LRR7** | 189 | 212 | TESLHIVFPTNKEFHFILDVSVKT |
| [**LRR**](http://smart.embl-heidelberg.de/smart/do_annotation.pl?DOMAIN=LRR&TYPE=SMART&START=355&END=378&LENGTH=23&E_VALUE=4.44083621375209&BLAST=LRCLEYLNMEDNDIPSIKRNMFTG)**8** | 213 | 245 | VANLELSNIKCVLEDNKCSYFLSILAKLQTNPK |
| [**LRR**](http://smart.embl-heidelberg.de/smart/do_annotation.pl?DOMAIN=LRR&TYPE=SMART&START=379&END=404&LENGTH=25&E_VALUE=87.3274593046497&BLAST=LINLRYLSLSNSFTNLRTLKNETFSS)**9** | 246 | 272 | LSNLTLNNIETTWNSFIRILQLVWHTT |
| [**LRR**](http://smart.embl-heidelberg.de/smart/do_annotation.pl?DOMAIN=LRR&TYPE=SMART&START=407&END=428&LENGTH=21&E_VALUE=131.25966102461&BLAST=HSPLLILNLTKNKISKIESDAF)**10** | 273 | 298 | VWYFSISNVKLQGQLDFRDF**D**YSGTS |
| [**LRR**](http://smart.embl-heidelberg.de/smart/do_annotation.pl?DOMAIN=LRR&TYPE=SMART&START=431&END=458&LENGTH=27&E_VALUE=324.191955411346&BLAST=LGSLEVLDIGINEIGQELTGQEWRGLEN)**11** | 299 | 322 | LKALSIHQVVSDVFGFPQSYIYEI |
| [**LRR**](http://smart.embl-heidelberg.de/smart/do_annotation.pl?DOMAIN=LRR&TYPE=SMART&START=506&END=524&LENGTH=18&E_VALUE=124.046876494985&BLAST=LHDLTILDLSNNNLANINE)**12** | 323 | 349 | FSNMNIKNFTVSGTRMVHMLCPSKISP |
| [**LRR**](http://smart.embl-heidelberg.de/smart/do_annotation.pl?DOMAIN=LRR&TYPE=SMART&START=530&END=564&LENGTH=34&E_VALUE=72.5089815799162&BLAST=LEKLEVLDLQHNNLARLWKQANPGGPVHFLKGLSH)**13** | 350 | 373 | FLHLDFSNNLLTDTVFENCGHLTE |
| **LRR14** | 374 | 399 | LETLILQMNQLKELSKIAEMTTQMKS |
| [**LRR**](http://smart.embl-heidelberg.de/smart/do_annotation.pl?DOMAIN=LRR&TYPE=SMART&START=586&END=605&LENGTH=19&E_VALUE=520.428720428041&BLAST=LFQLKSINLALNNLNVLPQS)**15** | 400 | 424 | LQQLDISQNSVSYDEKKGDCSWTKS |
| [**LRR**](http://smart.embl-heidelberg.de/smart/do_annotation.pl?DOMAIN=LRR&TYPE=SMART&START=611&END=633&LENGTH=22&E_VALUE=25.3611539551777&BLAST=VSLKSLNLQKNLITSVEKKVFGP)**16** | 425 | 446 | LLSLNMSSNILTDTIFRCLPPR |
| [**LRR**](http://smart.embl-heidelberg.de/smart/do_annotation.pl?DOMAIN=LRRCT&TYPE=SMART&START=646&END=698&LENGTH=52&E_VALUE=6.48840098134863e-10&BLAST=NPFDCTCESIAWFVNWINKTRTNISELSSHYLCNTPPQYHGFSVRLFDTSSCK)**17** | 447 | 469 | IKVLDLHSNKIKSIPKQVVKLEA |
| [**LRR**](http://smart.embl-heidelberg.de/smart/do_annotation.pl?DOMAIN=LRRCT&TYPE=SMART&START=646&END=698&LENGTH=52&E_VALUE=6.48840098134863e-10&BLAST=NPFDCTCESIAWFVNWINKTRTNISELSSHYLCNTPPQYHGFSVRLFDTSSCK)**18** | 470 | 491 | LQELNVAFNSLTDLPGCGSFSS |
| [**LRR**](http://smart.embl-heidelberg.de/smart/do_annotation.pl?DOMAIN=LRRCT&TYPE=SMART&START=646&END=698&LENGTH=52&E_VALUE=6.48840098134863e-10&BLAST=NPFDCTCESIAWFVNWINKTRTNISELSSHYLCNTPPQYHGFSVRLFDTSSCK)**19** | 492 | 515 | LSVLIIDHNSVSHPSADFFQSCQK |
| [**LRR**](http://smart.embl-heidelberg.de/smart/do_annotation.pl?DOMAIN=LRRCT&TYPE=SMART&START=646&END=698&LENGTH=52&E_VALUE=6.48840098134863e-10&BLAST=NPFDCTCESIAWFVNWINKTRTNISELSSHYLCNTPPQYHGFSVRLFDTSSCK)**20** | 516 | 539 | MRSIKAGDNPFQCTCELGEFVKNI |
| **LRR-CT** | 524 | 580 | NPFQCTCELGEFVKNIDQVSSEVLEGWPDSYKCDY PESYRGTLLKDFHMSELSCNIT |
| **Transmembrane** | 581 | 603 | LLIVTIVATMLVLAVTVTSLCSY |
| **TIR** | 604 | 786 | LDLPWYLRMVCQWTQTRRRARNIPLEELQRNLQFH AFISYSGHDSFWVKNELLPNLEKEGMQICLHERNF VPGKSIVENIITCIEKSYKSIFVLSPNFVQSEWCH YELYFAHHNLFHEGSNSLILILLEPIPQYSIPSSY HKLKSLMARRTYLEWPKEKSKRGLFWANLRAAINI KLTEQAKK |
