# Supplementary material for: Signatures of positive selection in Toll-like receptor (TLR) genes in mammals
Source: BMC Evol Biol. 2011 Dec 20;11:368. doi: 10.1186/1471-2148-11-368 (PMC3276489; doi:10.1186/1471-2148-11-368)
Supplement: Additional file 22 — Table S22. Domain characterization of TLR2. Microsoft Word document containing the list of domains of Human TLR2 gene, their delimitation and sequence. [file 1471-2148-11-368-S22.DOC]

Table S22. Domain characterization of TLR2.

**The conserved segment of each LRR is underlined. The amino acids identified as under positive selection are in bold.**

| **TLR2 – *Homo sapiens*** | | | |
| --- | --- | --- | --- |
| **Domain** | **Start** | **Stop** | **Sequence** |
| **Signal** | 1 | 17 | MP**H**TLWMVWVLGVIISL |
| [**LRR**](http://smart.embl-heidelberg.de/smart/do_annotation.pl?DOMAIN=LRR&TYPE=SMART&START=51&END=70&LENGTH=19&E_VALUE=69.0126970495531&BLAST=PTNITVLNLTHNQIKRLPPA)**-NT** | 18 | 53 | SKEESSNQASLSCDRNGICKGSSGSLNSIPSGLTEA |
| **LRR1** | 54 | 77 | VKSLDLSNNRITYISNSDLQRCVN |
| [**LRR**](http://smart.embl-heidelberg.de/smart/do_annotation.pl?DOMAIN=LRR&TYPE=SMART&START=123&END=144&LENGTH=21&E_VALUE=289.551614689825&BLAST=CMNLTELHLMSNSIQKIQNNPF)**2** | 78 | 101 | LQALVLTSNGINTIEEDSFSSLGS |
| [**LRR**](http://smart.embl-heidelberg.de/smart/do_annotation.pl?DOMAIN=LRR&TYPE=SMART&START=171&END=194&LENGTH=23&E_VALUE=57.8362009479994&BLAST=LQNLQELLLSKNKIQALKSEELAF)**3** | 102 | 125 | LEHLDLSYNYLSNLSSSWFKPLSS |
| [**LRR**](http://smart.embl-heidelberg.de/smart/do_annotation.pl?DOMAIN=LRR&TYPE=SMART&START=197&END=218&LENGTH=21&E_VALUE=384.070417219697&BLAST=NSSLKKLELSSNLIKEFSPGCF)**4** | 126 | 150 | LTFLNLLGNPYKTLGETSLFSHLTK |
| [**LRR**](http://smart.embl-heidelberg.de/smart/do_annotation.pl?DOMAIN=LRR&TYPE=SMART&START=197&END=218&LENGTH=21&E_VALUE=384.070417219697&BLAST=NSSLKKLELSSNLIKEFSPGCF)**5** | 151 | 175 | LQILRVGNMD**T**FTKIQRKDFAGLTF |
| [**LRR**](http://smart.embl-heidelberg.de/smart/do_annotation.pl?DOMAIN=LRR&TYPE=SMART&START=274&END=295&LENGTH=21&E_VALUE=6.4745441770878&BLAST=HTNLTMLDLSHNNLNMIDDDSF)**6** | 176 | 196 | LEELEI**D**ASDLQSYEPKSLKS |
| **LRR7** | 197 | 223 | IQNVSHLILHMKQHILLLEIFVDVTSS |
| [**LRR**](http://smart.embl-heidelberg.de/smart/do_annotation.pl?DOMAIN=LRR&TYPE=SMART&START=355&END=378&LENGTH=23&E_VALUE=4.44083621375209&BLAST=LRCLEYLNMEDNDIPSIKRNMFTG)**8** | 224 | 250 | VECLELRDTDLDTFHFSELSTGETNSL |
| [**LRR**](http://smart.embl-heidelberg.de/smart/do_annotation.pl?DOMAIN=LRR&TYPE=SMART&START=379&END=404&LENGTH=25&E_VALUE=87.3274593046497&BLAST=LINLRYLSLSNSFTNLRTLKNETFSS)**9** | 251 | 278 | IKKFTFRNVKITDESLFQVMKLLNQISG |
| [**LRR**](http://smart.embl-heidelberg.de/smart/do_annotation.pl?DOMAIN=LRR&TYPE=SMART&START=407&END=428&LENGTH=21&E_VALUE=131.25966102461&BLAST=HSPLLILNLTKNKISKIESDAF)**10** | 279 | 308 | LLELEFDDCTLNGVGNFRASDND**R**VIDPGK |
| [**LRR**](http://smart.embl-heidelberg.de/smart/do_annotation.pl?DOMAIN=LRR&TYPE=SMART&START=431&END=458&LENGTH=27&E_VALUE=324.191955411346&BLAST=LGSLEVLDIGINEIGQELTGQEWRGLEN)**11** | 309 | 337 | VETLTIRRLHIPRFYLFYDLSTLYSLTER |
| [**LRR**](http://smart.embl-heidelberg.de/smart/do_annotation.pl?DOMAIN=LRR&TYPE=SMART&START=506&END=524&LENGTH=18&E_VALUE=124.046876494985&BLAST=LHDLTILDLSNNNLANINE)**12** | 338 | 361 | VKRITVENSKVFLVPCLLSQHLKS |
| [**LRR**](http://smart.embl-heidelberg.de/smart/do_annotation.pl?DOMAIN=LRR&TYPE=SMART&START=530&END=564&LENGTH=34&E_VALUE=72.5089815799162&BLAST=LEKLEVLDLQHNNLARLWKQANPGGPVHFLKGLSH)**13** | 362 | 388 | LEYLDLSENLMVEEYLKNSACEDAWPS |
| **LRR14** | 389 | 414 | LQTLILRQNHLASLEKTGETLLTLKN |
| [**LRR**](http://smart.embl-heidelberg.de/smart/do_annotation.pl?DOMAIN=LRR&TYPE=SMART&START=586&END=605&LENGTH=19&E_VALUE=520.428720428041&BLAST=LFQLKSINLALNNLNVLPQS)**15** | 415 | 437 | LTNIDISKNSFHSMPETCQWPEK |
| [**LRR**](http://smart.embl-heidelberg.de/smart/do_annotation.pl?DOMAIN=LRR&TYPE=SMART&START=611&END=633&LENGTH=22&E_VALUE=25.3611539551777&BLAST=VSLKSLNLQKNLITSVEKKVFGP)**16** | 438 | 458 | MKYLNLSSTRIHSVTGCIPKT |
| [**LRR**](http://smart.embl-heidelberg.de/smart/do_annotation.pl?DOMAIN=LRRCT&TYPE=SMART&START=646&END=698&LENGTH=52&E_VALUE=6.48840098134863e-10&BLAST=NPFDCTCESIAWFVNWINKTRTNISELSSHYLCNTPPQYHGFSVRLFDTSSCK)**17** | 459 | 478 | LEILDVSNNNLNLFSLNLPQ |
| [**LRR**](http://smart.embl-heidelberg.de/smart/do_annotation.pl?DOMAIN=LRRCT&TYPE=SMART&START=646&END=698&LENGTH=52&E_VALUE=6.48840098134863e-10&BLAST=NPFDCTCESIAWFVNWINKTRTNISELSSHYLCNTPPQYHGFSVRLFDTSSCK)**18** | 479 | 500 | LKELYISRNKLMTLPDASLLPM |
| [**LRR**](http://smart.embl-heidelberg.de/smart/do_annotation.pl?DOMAIN=LRRCT&TYPE=SMART&START=646&END=698&LENGTH=52&E_VALUE=6.48840098134863e-10&BLAST=NPFDCTCESIAWFVNWINKTRTNISELSSHYLCNTPPQYHGFSVRLFDTSSCK)**19** | 501 | 524 | LLVLKISRNAITTFSKEQLDSFHT |
| [**LRR**](http://smart.embl-heidelberg.de/smart/do_annotation.pl?DOMAIN=LRRCT&TYPE=SMART&START=646&END=698&LENGTH=52&E_VALUE=6.48840098134863e-10&BLAST=NPFDCTCESIAWFVNWINKTRTNISELSSHYLCNTPPQYHGFSVRLFDTSSCK)**20** | 525 | 548 | LKTLEAGGNNFICSCEFLSFTQEQ |
| **LRR-CT** | 533 | 587 | NNFICSCEFLSFTQEQQALAKVLIDWPANYLCDSP SHVRGQQVQDVRLSVSECHR |
| **Transmembrane** | 588 | 610 | TALVSGMCCALFLL**I**LLTGVLCH |
| **TIR** | 611 | 784 | RFHGLWYMKMMWAWLQAKRKPRKAP**S**RNICYDAFV SYSERDAYWVENLMVQELENFNPPFKLCLHKRDFI PGKWIIDNIIDSIEKSHKTVFVLSENFVKSEWCKY ELDFSHFRLFDENNDAAILILLEPIEKKAIPQRFC KLRKIMNTKTYLEWPMDEAQREGFWVNLRAAIKS |
