# Supplementary material for: Signatures of positive selection in Toll-like receptor (TLR) genes in mammals
Source: BMC Evol Biol. 2011 Dec 20;11:368. doi: 10.1186/1471-2148-11-368 (PMC3276489; doi:10.1186/1471-2148-11-368)
Supplement: Additional file 23 — Table S23. Domain characterization of TLR3. Microsoft Word document containing the list of domains of Human TLR3 gene, their delimitation and sequence. [file 1471-2148-11-368-S23.DOC]

Table S23. Domain characterization of TLR3.

**The conserved segment of each LRR is underlined. The amino acids identified as under positive selection are in bold.**

| **TLR3 – *Homo sapiens*** | | | |
| --- | --- | --- | --- |
| **Domain** | **Start** | **Stop** | **Sequence** |
| **Signal** | 1 | 25 | MRQ**T**LPCIYFW**G**GLLPFGMLCASS**T** |
| [**LRR**](http://smart.embl-heidelberg.de/smart/do_annotation.pl?DOMAIN=LRR&TYPE=SMART&START=51&END=70&LENGTH=19&E_VALUE=69.0126970495531&BLAST=PTNITVLNLTHNQIKRLPPA)**-NT** | 26 | 52 | TKCTVSHEVADCSHLKLTQVPDDLPTN |
| **LRR1** | 53 | 76 | ITVLNLTHNQLRRLPAANFTRYSQ |
| [**LRR**](http://smart.embl-heidelberg.de/smart/do_annotation.pl?DOMAIN=LRR&TYPE=SMART&START=123&END=144&LENGTH=21&E_VALUE=289.551614689825&BLAST=CMNLTELHLMSNSIQKIQNNPF)**2** | 77 | 100 | LT**S**LDVGFNTISKLEPELCQKLPM |
| [**LRR**](http://smart.embl-heidelberg.de/smart/do_annotation.pl?DOMAIN=LRR&TYPE=SMART&START=171&END=194&LENGTH=23&E_VALUE=57.8362009479994&BLAST=LQNLQELLLSKNKIQALKSEELAF)**3** | 101 | 124 | LKVLNLQHNELSQLSDKTFAFCTN |
| [**LRR**](http://smart.embl-heidelberg.de/smart/do_annotation.pl?DOMAIN=LRR&TYPE=SMART&START=197&END=218&LENGTH=21&E_VALUE=384.070417219697&BLAST=NSSLKKLELSSNLIKEFSPGCF)**4** | 125 | 148 | LTELHLMSNSIQKIKNNPFVKQKN |
| [**LRR**](http://smart.embl-heidelberg.de/smart/do_annotation.pl?DOMAIN=LRR&TYPE=SMART&START=197&END=218&LENGTH=21&E_VALUE=384.070417219697&BLAST=NSSLKKLELSSNLIKEFSPGCF)**5** | 149 | 172 | LITLDLSHNGLSSTKLGTQVQLEN |
| [**LRR**](http://smart.embl-heidelberg.de/smart/do_annotation.pl?DOMAIN=LRR&TYPE=SMART&START=274&END=295&LENGTH=21&E_VALUE=6.4745441770878&BLAST=HTNLTMLDLSHNNLNMIDDDSF)**6** | 173 | 198 | LQELLLSNNKIQALKSEELDIFANSS |
| **LRR7** | 199 | 222 | LKKLELSSNQIKEFSPGCFHAIGR |
| [**LRR**](http://smart.embl-heidelberg.de/smart/do_annotation.pl?DOMAIN=LRR&TYPE=SMART&START=355&END=378&LENGTH=23&E_VALUE=4.44083621375209&BLAST=LRCLEYLNMEDNDIPSIKRNMFTG)**8** | 223 | 249 | LFGLFLNNVQLGPSLTEKLCLELANTS |
| [**LRR**](http://smart.embl-heidelberg.de/smart/do_annotation.pl?DOMAIN=LRR&TYPE=SMART&START=379&END=404&LENGTH=25&E_VALUE=87.3274593046497&BLAST=LINLRYLSLSNSFTNLRTLKNETFSS)**9** | 250 | 275 | IRNLSLSN**S**QLSTTSNTTFLGLKWTN |
| [**LRR**](http://smart.embl-heidelberg.de/smart/do_annotation.pl?DOMAIN=LRR&TYPE=SMART&START=407&END=428&LENGTH=21&E_VALUE=131.25966102461&BLAST=HSPLLILNLTKNKISKIESDAF)**10** | 276 | 299 | LTMLDLSYN**N**LNVVGNDSFAWLPQ |
| [**LRR**](http://smart.embl-heidelberg.de/smart/do_annotation.pl?DOMAIN=LRR&TYPE=SMART&START=431&END=458&LENGTH=27&E_VALUE=324.191955411346&BLAST=LGSLEVLDIGINEIGQELTGQEWRGLEN)**11** | 300 | 323 | LEYFFLEYNNIQHLFSHSLHGLFN |
| [**LRR**](http://smart.embl-heidelberg.de/smart/do_annotation.pl?DOMAIN=LRR&TYPE=SMART&START=506&END=524&LENGTH=18&E_VALUE=124.046876494985&BLAST=LHDLTILDLSNNNLANINE)**12** | 324 | 356 | VRYLNLKRSFTKQSISLASLPKIDDFSFQWLKC |
| [**LRR**](http://smart.embl-heidelberg.de/smart/do_annotation.pl?DOMAIN=LRR&TYPE=SMART&START=530&END=564&LENGTH=34&E_VALUE=72.5089815799162&BLAST=LEKLEVLDLQHNNLARLWKQANPGGPVHFLKGLSH)**13** | 357 | 380 | LEHLNMEDNDIPGIKSNMFTGLIN |
| **LRR14** | 381 | 408 | LKYLSLSNSFTSLRTLTNETFVSLAHSP |
| [**LRR**](http://smart.embl-heidelberg.de/smart/do_annotation.pl?DOMAIN=LRR&TYPE=SMART&START=586&END=605&LENGTH=19&E_VALUE=520.428720428041&BLAST=LFQLKSINLALNNLNVLPQS)**15** | 409 | 432 | LHILNLTKNKISKIESDAFSWLGH |
| [**LRR**](http://smart.embl-heidelberg.de/smart/do_annotation.pl?DOMAIN=LRR&TYPE=SMART&START=611&END=633&LENGTH=22&E_VALUE=25.3611539551777&BLAST=VSLKSLNLQKNLITSVEKKVFGP)**16** | 433 | 457 | LEVLDLGLNEIGQELTGQEWRGLEN |
| [**LRR**](http://smart.embl-heidelberg.de/smart/do_annotation.pl?DOMAIN=LRRCT&TYPE=SMART&START=646&END=698&LENGTH=52&E_VALUE=6.48840098134863e-10&BLAST=NPFDCTCESIAWFVNWINKTRTNISELSSHYLCNTPPQYHGFSVRLFDTSSCK)**17** | 458 | 481 | IFEIYLSYNKYLQLTRNSFALVPS |
| [**LRR**](http://smart.embl-heidelberg.de/smart/do_annotation.pl?DOMAIN=LRRCT&TYPE=SMART&START=646&END=698&LENGTH=52&E_VALUE=6.48840098134863e-10&BLAST=NPFDCTCESIAWFVNWINKTRTNISELSSHYLCNTPPQYHGFSVRLFDTSSCK)**18** | 482 | 507 | LQRLMLRRVALKNVDSSPSPFQPLRN |
| [**LRR**](http://smart.embl-heidelberg.de/smart/do_annotation.pl?DOMAIN=LRRCT&TYPE=SMART&START=646&END=698&LENGTH=52&E_VALUE=6.48840098134863e-10&BLAST=NPFDCTCESIAWFVNWINKTRTNISELSSHYLCNTPPQYHGFSVRLFDTSSCK)**19** | 508 | 531 | LTILDLSNNNIANINDDMLEGLEK |
| [**LRR**](http://smart.embl-heidelberg.de/smart/do_annotation.pl?DOMAIN=LRRCT&TYPE=SMART&START=646&END=698&LENGTH=52&E_VALUE=6.48840098134863e-10&BLAST=NPFDCTCESIAWFVNWINKTRTNISELSSHYLCNTPPQYHGFSVRLFDTSSCK)**20** | 532 | 563 | LEILDLQHNNLARLWKHANPGGPIYFLKGLSH |
| **LRR21** | 564 | 587 | LHILNLESNGFDEIPVEVFKDLFE |
| **LRR22** | 588 | 611 | LKIIDLGLNNLNTLPASVFNNQVS |
| **LRR23** | 612 | 636 | LKSLNLQKNLITSVEKKVFGPAFRN |
| **LRR24** | 637 | 660 | LTELDMRFNPFDCTCESIAWFVNW |
| **LRR-CT** | 645 | 701 | NPFDCTCESIAWFVNWINETHTNIPELSSHYLCNT PPHYHGFPVRLFDTSSCKDSAP |
| **Transmembrane** | 702 | 724 | FELFFMINTS**I**LLIFIFIVLLIH |
| **TIR** | 725 | 904 | FEGWRISFYWNVSVHRVLGFKEID**R**QTEQFEYAAY IIHAYKDKDWVWEHFSSM**E**KEDQSLKFCLEERDFE AGVFELEAIVNSIKRSRKIIFVITHHLLKDPLCKR FKVHHAVQQAIEQNLDSIILVFLEEIPDYKLNHAL CLRRGMFKSHCILNWPVQKERIGAFRHKLQVALGS KNSVH |
