# Supplementary material for: Signatures of positive selection in Toll-like receptor (TLR) genes in mammals
Source: BMC Evol Biol. 2011 Dec 20;11:368. doi: 10.1186/1471-2148-11-368 (PMC3276489; doi:10.1186/1471-2148-11-368)
Supplement: Additional file 24 — Table S24. Domain characterization of TLR4. Microsoft Word document containing the list of domains of Human TLR4 gene, their delimitation and sequence. [file 1471-2148-11-368-S24.DOC]

Table S24. Domain characterization of TLR4.

**The conserved segment of each LRR is underlined. The amino acids identified as under positive selection are in bold.**

| **TLR4 – *Homo sapiens*** | | | |
| --- | --- | --- | --- |
| **Domain** | **Start** | **Stop** | **Sequence** |
| **Signal** | 1 | 23 | MMSASRLAGTLIPAMAFLSCVRP |
| [**LRR**](http://smart.embl-heidelberg.de/smart/do_annotation.pl?DOMAIN=LRR&TYPE=SMART&START=51&END=70&LENGTH=19&E_VALUE=69.0126970495531&BLAST=PTNITVLNLTHNQIKRLPPA)**-NT** | 24 | 55 | ESWEPCVEVVPNITYQCMELNFYKIPDNLPFS |
| **LRR1** | 56 | 79 | TKNLDLSFNPLRHLGSYSFFSFPE |
| [**LRR**](http://smart.embl-heidelberg.de/smart/do_annotation.pl?DOMAIN=LRR&TYPE=SMART&START=123&END=144&LENGTH=21&E_VALUE=289.551614689825&BLAST=CMNLTELHLMSNSIQKIQNNPF)**2** | 80 | 103 | LQVLDLSRCEIQTIEDGAYQSLSH |
| [**LRR**](http://smart.embl-heidelberg.de/smart/do_annotation.pl?DOMAIN=LRR&TYPE=SMART&START=171&END=194&LENGTH=23&E_VALUE=57.8362009479994&BLAST=LQNLQELLLSKNKIQALKSEELAF)**3** | 104 | 127 | LSTLILTGNPIQSLALGAFSGLSS |
| [**LRR**](http://smart.embl-heidelberg.de/smart/do_annotation.pl?DOMAIN=LRR&TYPE=SMART&START=197&END=218&LENGTH=21&E_VALUE=384.070417219697&BLAST=NSSLKKLELSSNLIKEFSPGCF)**4** | 128 | 151 | LQKLVAVETNLASLENFPIGHLKT |
| [**LRR**](http://smart.embl-heidelberg.de/smart/do_annotation.pl?DOMAIN=LRR&TYPE=SMART&START=197&END=218&LENGTH=21&E_VALUE=384.070417219697&BLAST=NSSLKKLELSSNLIKEFSPGCF)**5** | 152 | 176 | LKELNVAHNLIQSFKLPEYFSNLTN |
| [**LRR**](http://smart.embl-heidelberg.de/smart/do_annotation.pl?DOMAIN=LRR&TYPE=SMART&START=274&END=295&LENGTH=21&E_VALUE=6.4745441770878&BLAST=HTNLTMLDLSHNNLNMIDDDSF)**6** | 177 | 204 | LEHLDLSSNKIQSIYCTDLRVLHQMPL**L** |
| **LRR7** | 205 | 227 | NLSLDLSLNPMNFIQPGAFKEIR |
| [**LRR**](http://smart.embl-heidelberg.de/smart/do_annotation.pl?DOMAIN=LRR&TYPE=SMART&START=355&END=378&LENGTH=23&E_VALUE=4.44083621375209&BLAST=LRCLEYLNMEDNDIPSIKRNMFTG)**8** | 228 | 249 | LHKLTLRNNFDS**L**NVMKTCIQG |
| [**LRR**](http://smart.embl-heidelberg.de/smart/do_annotation.pl?DOMAIN=LRR&TYPE=SMART&START=379&END=404&LENGTH=25&E_VALUE=87.3274593046497&BLAST=LINLRYLSLSNSFTNLRTLKNETFSS)**9** | 250 | 279 | LAGLEVHRLVLGEFRNEGNL**E**KFDKS**A**LEG |
| [**LRR**](http://smart.embl-heidelberg.de/smart/do_annotation.pl?DOMAIN=LRR&TYPE=SMART&START=407&END=428&LENGTH=21&E_VALUE=131.25966102461&BLAST=HSPLLILNLTKNKISKIESDAF)**10** | 280 | 309 | LCNLTIEEFRLAYLD**Y**YLDD**II**DLFNCLTN |
| [**LRR**](http://smart.embl-heidelberg.de/smart/do_annotation.pl?DOMAIN=LRR&TYPE=SMART&START=431&END=458&LENGTH=27&E_VALUE=324.191955411346&BLAST=LGSLEVLDIGINEIGQELTGQEWRGLEN)**11** | 310 | 331 | VSSFSLV**S**V**T**IERVKDFSYNFG |
| [**LRR**](http://smart.embl-heidelberg.de/smart/do_annotation.pl?DOMAIN=LRR&TYPE=SMART&START=506&END=524&LENGTH=18&E_VALUE=124.046876494985&BLAST=LHDLTILDLSNNNLANINE)**12** | 332 | 352 | WQHLELVNCKFGQFPTLKLKS |
| [**LRR**](http://smart.embl-heidelberg.de/smart/do_annotation.pl?DOMAIN=LRR&TYPE=SMART&START=530&END=564&LENGTH=34&E_VALUE=72.5089815799162&BLAST=LEKLEVLDLQHNNLARLWKQANPGGPVHFLKGLSH)**13** | 353 | 374 | LKR**L**TFTSNK**G**GNAFSE**V**DLPS |
| **LRR14** | 375 | 400 | LEFLDLS**R**NGLSFKGCCSQ**S**DFGTTS |
| [**LRR**](http://smart.embl-heidelberg.de/smart/do_annotation.pl?DOMAIN=LRR&TYPE=SMART&START=586&END=605&LENGTH=19&E_VALUE=520.428720428041&BLAST=LFQLKSINLALNNLNVLPQS)**15** | 401 | 423 | LKYLDLSFNGVITMSSNFLGLEQ |
| [**LRR**](http://smart.embl-heidelberg.de/smart/do_annotation.pl?DOMAIN=LRR&TYPE=SMART&START=611&END=633&LENGTH=22&E_VALUE=25.3611539551777&BLAST=VSLKSLNLQKNLITSVEKKVFGP)**16** | 424 | 448 | LEHLDFQHSNLKQMSEFSVFLSLRN |
| [**LRR**](http://smart.embl-heidelberg.de/smart/do_annotation.pl?DOMAIN=LRRCT&TYPE=SMART&START=646&END=698&LENGTH=52&E_VALUE=6.48840098134863e-10&BLAST=NPFDCTCESIAWFVNWINKTRTNISELSSHYLCNTPPQYHGFSVRLFDTSSCK)**17** | 449 | 472 | LIYLDISHTHTRVAFNGIF**N**GL**S**S |
| [**LRR**](http://smart.embl-heidelberg.de/smart/do_annotation.pl?DOMAIN=LRRCT&TYPE=SMART&START=646&END=698&LENGTH=52&E_VALUE=6.48840098134863e-10&BLAST=NPFDCTCESIAWFVNWINKTRTNISELSSHYLCNTPPQYHGFSVRLFDTSSCK)**18** | 473 | 497 | LEVLKMAGNSFQEN**F**LPDIFTELRN |
| [**LRR**](http://smart.embl-heidelberg.de/smart/do_annotation.pl?DOMAIN=LRRCT&TYPE=SMART&START=646&END=698&LENGTH=52&E_VALUE=6.48840098134863e-10&BLAST=NPFDCTCESIAWFVNWINKTRTNISELSSHYLCNTPPQYHGFSVRLFDTSSCK)**19** | 498 | 521 | LT**F**LDLSQCQLEQLSPTAFNSLSS |
| [**LRR**](http://smart.embl-heidelberg.de/smart/do_annotation.pl?DOMAIN=LRRCT&TYPE=SMART&START=646&END=698&LENGTH=52&E_VALUE=6.48840098134863e-10&BLAST=NPFDCTCESIAWFVNWINKTRTNISELSSHYLCNTPPQYHGFSVRLFDTSSCK)**20** | 522 | 545 | LQVLNMSHNNFFSLDTFPYK**C**LNS |
| **LRR21** | 546 | 570 | LQVLDYSLNHIMTSKKQELQHFPSS |
| **LRR22** | 571 | 594 | LAFLNLTQNDFACTCEHQSFLQWI |
| **LRR-CT** | 579 | 632 | NDFACTCEHQSFLQWIKDQRQLLVE**V**ERMECATPS DKQGMPVLSLNITCQMNKT |
| **Transmembrane** | 633 | 652 | IIGVSV**L**SVLVVSVVAVLVY |
| **TIR** | 653 | 839 | KFYFHLMLLAGCIKYGRGEN**I**YDAFVIYSSQDEDW VRNELVKNLEEGVPPFQLCLHYRDFIPGVAIAANI IHEGFHKSRKVIVVVSQHFIQSRWCIFEYEIAQTW QFLSSRAGIIFIVLQKVEKTLLRQQVELYRLLSRN TYLEWEDSVLGRHIFWRRLRKALLDGKSWNPEGTV GTGCNWQEATSI |
