# Supplementary material for: Signatures of positive selection in Toll-like receptor (TLR) genes in mammals
Source: BMC Evol Biol. 2011 Dec 20;11:368. doi: 10.1186/1471-2148-11-368 (PMC3276489; doi:10.1186/1471-2148-11-368)
Supplement: Additional file 25 — Table S25. Domain characterization of TLR5. Microsoft Word document containing the list of domains of Human TLR5 gene, their delimitation and sequence. [file 1471-2148-11-368-S25.DOC]

Table S25. Domain characterization of TLR5.

**The conserved segment of each LRR is underlined. The amino acids identified as under positive selection are in bold.**

| **TLR5 – *Homo sapiens*** | | | |
| --- | --- | --- | --- |
| **Domain** | **Start** | **Stop** | **Sequence** |
| **Signal** | 1 | 20 | MGDHLDLLLGVVL**M**AGPVFG |
| [**LRR**](http://smart.embl-heidelberg.de/smart/do_annotation.pl?DOMAIN=LRR&TYPE=SMART&START=51&END=70&LENGTH=19&E_VALUE=69.0126970495531&BLAST=PTNITVLNLTHNQIKRLPPA)**-NT** | 21 | 47 | IPSCSFDGRIAFYRFCNLTQVPQVLNT |
| **LRR1** | 48 | 71 | TERLLLSFNYIRTVTASSFPFLEQ |
| [**LRR**](http://smart.embl-heidelberg.de/smart/do_annotation.pl?DOMAIN=LRR&TYPE=SMART&START=123&END=144&LENGTH=21&E_VALUE=289.551614689825&BLAST=CMNLTELHLMSNSIQKIQNNPF)**2** | 72 | 96 | LQLLELGSQYTPLTIDKEAFRNLPN |
| [**LRR**](http://smart.embl-heidelberg.de/smart/do_annotation.pl?DOMAIN=LRR&TYPE=SMART&START=171&END=194&LENGTH=23&E_VALUE=57.8362009479994&BLAST=LQNLQELLLSKNKIQALKSEELAF)**3** | 97 | 120 | LRILDLGSSKIYFLHPDAFQGLFH |
| [**LRR**](http://smart.embl-heidelberg.de/smart/do_annotation.pl?DOMAIN=LRR&TYPE=SMART&START=197&END=218&LENGTH=21&E_VALUE=384.070417219697&BLAST=NSSLKKLELSSNLIKEFSPGCF)**4** | 121 | 146 | LFELRLY**F**CGLSDAVLKDGYFRNLKA |
| [**LRR**](http://smart.embl-heidelberg.de/smart/do_annotation.pl?DOMAIN=LRR&TYPE=SMART&START=197&END=218&LENGTH=21&E_VALUE=384.070417219697&BLAST=NSSLKKLELSSNLIKEFSPGCF)**5** | 147 | 171 | LTRLDLSKNQIRSLYLHPSFGKLNS |
| [**LRR**](http://smart.embl-heidelberg.de/smart/do_annotation.pl?DOMAIN=LRR&TYPE=SMART&START=274&END=295&LENGTH=21&E_VALUE=6.4745441770878&BLAST=HTNLTMLDLSHNNLNMIDDDSF)**6** | 172 | 197 | LKSIDFSSNQIFLVCEHELEPLQGKT |
| **LRR7** | 198 | 227 | LSFFSLAAN**S**LYSRVSVDWGKCMNPFRNMV |
| [**LRR**](http://smart.embl-heidelberg.de/smart/do_annotation.pl?DOMAIN=LRR&TYPE=SMART&START=355&END=378&LENGTH=23&E_VALUE=4.44083621375209&BLAST=LRCLEYLNMEDNDIPSIKRNMFTG)**8** | 228 | 254 | LEILDVSGNGWTVDITGNFSNAISKSQ |
| [**LRR**](http://smart.embl-heidelberg.de/smart/do_annotation.pl?DOMAIN=LRR&TYPE=SMART&START=379&END=404&LENGTH=25&E_VALUE=87.3274593046497&BLAST=LINLRYLSLSNSFTNLRTLKNETFSS)**9** | 255 | 289 | AFSLILAHHIMGAGFGFHNIKDPDQNTFAGLARSS |
| [**LRR**](http://smart.embl-heidelberg.de/smart/do_annotation.pl?DOMAIN=LRR&TYPE=SMART&START=407&END=428&LENGTH=21&E_VALUE=131.25966102461&BLAST=HSPLLILNLTKNKISKIESDAF)**10** | 290 | 313 | VRHLDLSHGFVFSLNSRVFETLKD |
| [**LRR**](http://smart.embl-heidelberg.de/smart/do_annotation.pl?DOMAIN=LRR&TYPE=SMART&START=431&END=458&LENGTH=27&E_VALUE=324.191955411346&BLAST=LGSLEVLDIGINEIGQELTGQEWRGLEN)**11** | 314 | 337 | LKVLNLAYNKINKIADEAFYGLDN |
| [**LRR**](http://smart.embl-heidelberg.de/smart/do_annotation.pl?DOMAIN=LRR&TYPE=SMART&START=506&END=524&LENGTH=18&E_VALUE=124.046876494985&BLAST=LHDLTILDLSNNNLANINE)**12** | 338 | 361 | LQVLNLSYNLLGELYSSNFYGLPK |
| [**LRR**](http://smart.embl-heidelberg.de/smart/do_annotation.pl?DOMAIN=LRR&TYPE=SMART&START=530&END=564&LENGTH=34&E_VALUE=72.5089815799162&BLAST=LEKLEVLDLQHNNLARLWKQANPGGPVHFLKGLSH)**13** | 362 | 385 | VAYIDLQKNHIAIIQDQTFKFLEK |
| **LRR14** | 386 | 404 | LQTLDLRDNALTTI**H**FIPS |
| [**LRR**](http://smart.embl-heidelberg.de/smart/do_annotation.pl?DOMAIN=LRR&TYPE=SMART&START=586&END=605&LENGTH=19&E_VALUE=520.428720428041&BLAST=LFQLKSINLALNNLNVLPQS)**15** | 405 | 424 | IPDIFLSGNKLVTLPKINLT |
| [**LRR**](http://smart.embl-heidelberg.de/smart/do_annotation.pl?DOMAIN=LRR&TYPE=SMART&START=611&END=633&LENGTH=22&E_VALUE=25.3611539551777&BLAST=VSLKSLNLQKNLITSVEKKVFGP)**16** | 425 | 449 | ANLIHLSENRLENLDILYFLLRVPH |
| [**LRR**](http://smart.embl-heidelberg.de/smart/do_annotation.pl?DOMAIN=LRRCT&TYPE=SMART&START=646&END=698&LENGTH=52&E_VALUE=6.48840098134863e-10&BLAST=NPFDCTCESIAWFVNWINKTRTNISELSSHYLCNTPPQYHGFSVRLFDTSSCK)**17** | 450 | 474 | LQILILNQNRFSSCSGDQTPSENPS |
| [**LRR**](http://smart.embl-heidelberg.de/smart/do_annotation.pl?DOMAIN=LRRCT&TYPE=SMART&START=646&END=698&LENGTH=52&E_VALUE=6.48840098134863e-10&BLAST=NPFDCTCESIAWFVNWINKTRTNISELSSHYLCNTPPQYHGFSVRLFDTSSCK)**18** | 475 | 503 | LEQLFLGENMLQLAWETELCWDVFEGLSH |
| [**LRR**](http://smart.embl-heidelberg.de/smart/do_annotation.pl?DOMAIN=LRRCT&TYPE=SMART&START=646&END=698&LENGTH=52&E_VALUE=6.48840098134863e-10&BLAST=NPFDCTCESIAWFVNWINKTRTNISELSSHYLCNTPPQYHGFSVRLFDTSSCK)**19** | 504 | 527 | LQVLYLNHNYLNSLPPGVFSHLTA |
| [**LRR**](http://smart.embl-heidelberg.de/smart/do_annotation.pl?DOMAIN=LRRCT&TYPE=SMART&START=646&END=698&LENGTH=52&E_VALUE=6.48840098134863e-10&BLAST=NPFDCTCESIAWFVNWINKTRTNISELSSHYLCNTPPQYHGFSVRLFDTSSCK)**20** | 528 | 549 | LRGLSLNSNRLTVLSHNDLPAN |
| **LRR21** | 550 | 570 | LEILDISRNQLLAPNPDVFVS |
| **LRR22** | 571 | 594 | LSVLDITHNKFICECELSTFINWL |
| **LRR-CT** | 579 | 637 | NKFICECELSTFINWLNHTNVTIAGPPADIYCVYP DSFSGVSLFSLSTEGCDEEEVLKS |
| **Transmembrane** | 638 | 660 | LKFSLFIVCTVTLTLFLMTILTV |
| **TIR** | 661 | 858 | TKFRGFCFICYKT**A**QRLVFKDHPQGTEPDMYKYDA YLCFSSKDFTWVQNALLKHLDTQYS**D**QNRFNLCFE ERDFVPGENRI**A**NIQDAIWNSRKIVCLVSRHFLRD GWCLEAFSYAQGRCLSDLNSALIMVVVGSLSQYQL MKHQSIRGFVQKQQYLRWPEDFQDVGWFLHKLSQQ ILKKEKEKKKDNNIPLQTVATIS |
