# Supplementary material for: Signatures of positive selection in Toll-like receptor (TLR) genes in mammals
Source: BMC Evol Biol. 2011 Dec 20;11:368. doi: 10.1186/1471-2148-11-368 (PMC3276489; doi:10.1186/1471-2148-11-368)
Supplement: Additional file 26 — Table S26. Domain characterization of TLR6. Microsoft Word document containing the list of domains of Human TLR6 gene, their delimitation and sequence. [file 1471-2148-11-368-S26.DOC]

Table S26. Domain characterization of TLR6.

**The conserved segment of each LRR is underlined. The amino acids identified as under positive selection are in bold.**

| **TLR6 – *Homo sapiens*** | | | |
| --- | --- | --- | --- |
| **Domain** | **Start** | **Stop** | **Sequence** |
| **Signal** | 1 | 23 | MTKDKEPIVKSFHFVCLMIIIVG |
| [**LRR**](http://smart.embl-heidelberg.de/smart/do_annotation.pl?DOMAIN=LRR&TYPE=SMART&START=51&END=70&LENGTH=19&E_VALUE=69.0126970495531&BLAST=PTNITVLNLTHNQIKRLPPA)**-NT** | 24 | 53 | TRIQFSDGNEFAVDKSKRGLIHVPKDLPLK |
| **LRR1** | 54 | 77 | TKVLDMSQNYIAELQVSDMSFLSE |
| [**LRR**](http://smart.embl-heidelberg.de/smart/do_annotation.pl?DOMAIN=LRR&TYPE=SMART&START=123&END=144&LENGTH=21&E_VALUE=289.551614689825&BLAST=CMNLTELHLMSNSIQKIQNNPF)**2** | 78 | 101 | LTVLRLSHNRIQLLDLSVFKFNQD |
| [**LRR**](http://smart.embl-heidelberg.de/smart/do_annotation.pl?DOMAIN=LRR&TYPE=SMART&START=171&END=194&LENGTH=23&E_VALUE=57.8362009479994&BLAST=LQNLQELLLSKNKIQALKSEELAF)**3** | 102 | 122 | LEYLDLSHNQLQKISCHPIVS |
| [**LRR**](http://smart.embl-heidelberg.de/smart/do_annotation.pl?DOMAIN=LRR&TYPE=SMART&START=197&END=218&LENGTH=21&E_VALUE=384.070417219697&BLAST=NSSLKKLELSSNLIKEFSPGCF)**4** | 123 | 147 | FRHLDLSFNDFKALPICKEFGNLSQ |
| [**LRR**](http://smart.embl-heidelberg.de/smart/do_annotation.pl?DOMAIN=LRR&TYPE=SMART&START=197&END=218&LENGTH=21&E_VALUE=384.070417219697&BLAST=NSSLKKLELSSNLIKEFSPGCF)**5** | 148 | 172 | LNFLGLSAMKLQKLDLLPIAHLHLS |
| [**LRR**](http://smart.embl-heidelberg.de/smart/do_annotation.pl?DOMAIN=LRR&TYPE=SMART&START=274&END=295&LENGTH=21&E_VALUE=6.4745441770878&BLAST=HTNLTMLDLSHNNLNMIDDDSF)**6** | 173 | 197 | YILLDLRNYYIKENETESLQILNAK |
| **LRR7** | 198 | 219 | TLHLVFHPTSLFAIQVNISVNT |
| [**LRR**](http://smart.embl-heidelberg.de/smart/do_annotation.pl?DOMAIN=LRR&TYPE=SMART&START=355&END=378&LENGTH=23&E_VALUE=4.44083621375209&BLAST=LRCLEYLNMEDNDIPSIKRNMFTG)**8** | 220 | 248 | LGCLQLTNIKLNDDNCQVFIKFLSELTRG |
| [**LRR**](http://smart.embl-heidelberg.de/smart/do_annotation.pl?DOMAIN=LRR&TYPE=SMART&START=379&END=404&LENGTH=25&E_VALUE=87.3274593046497&BLAST=LINLRYLSLSNSFTNLRTLKNETFSS)**9** | 249 | 277 | PTLLNFTLNHIETTWKCLVRVFQFLWPKP |
| [**LRR**](http://smart.embl-heidelberg.de/smart/do_annotation.pl?DOMAIN=LRR&TYPE=SMART&START=407&END=428&LENGTH=21&E_VALUE=131.25966102461&BLAST=HSPLLILNLTKNKISKIESDAF)**10** | 278 | 303 | VEYLNIYNLTIIESIREEDFTYSKTT |
| [**LRR**](http://smart.embl-heidelberg.de/smart/do_annotation.pl?DOMAIN=LRR&TYPE=SMART&START=431&END=458&LENGTH=27&E_VALUE=324.191955411346&BLAST=LGSLEVLDIGINEIGQELTGQEWRGLEN)**11** | 304 | 332 | LKALTIEHITNQVFLFSQTALYTVFSEMN |
| [**LRR**](http://smart.embl-heidelberg.de/smart/do_annotation.pl?DOMAIN=LRR&TYPE=SMART&START=506&END=524&LENGTH=18&E_VALUE=124.046876494985&BLAST=LHDLTILDLSNNNLANINE)**12** | 333 | 354 | IMMLTISDTPFIHMLCPHAPST |
| [**LRR**](http://smart.embl-heidelberg.de/smart/do_annotation.pl?DOMAIN=LRR&TYPE=SMART&START=530&END=564&LENGTH=34&E_VALUE=72.5089815799162&BLAST=LEKLEVLDLQHNNLARLWKQANPGGPVHFLKGLSH)**13** | 355 | 378 | FKFLNFTQNVFTDSIFEKCSTLVK |
| **LRR14** | 379 | 404 | LETLILQKNGLKDLFKVGLMTKDMPS |
| [**LRR**](http://smart.embl-heidelberg.de/smart/do_annotation.pl?DOMAIN=LRR&TYPE=SMART&START=586&END=605&LENGTH=19&E_VALUE=520.428720428041&BLAST=LFQLKSINLALNNLNVLPQS)**15** | 405 | 429 | LEILDVSWNSLESGRHKENCTWVES |
| [**LRR**](http://smart.embl-heidelberg.de/smart/do_annotation.pl?DOMAIN=LRR&TYPE=SMART&START=611&END=633&LENGTH=22&E_VALUE=25.3611539551777&BLAST=VSLKSLNLQKNLITSVEKKVFGP)**16** | 430 | 451 | IVVLNLSSNMLTDSVFRCLPPR |
| [**LRR**](http://smart.embl-heidelberg.de/smart/do_annotation.pl?DOMAIN=LRRCT&TYPE=SMART&START=646&END=698&LENGTH=52&E_VALUE=6.48840098134863e-10&BLAST=NPFDCTCESIAWFVNWINKTRTNISELSSHYLCNTPPQYHGFSVRLFDTSSCK)**17** | 452 | 474 | IKVLDLHSNKIKSVPKQVVKLEA |
| [**LRR**](http://smart.embl-heidelberg.de/smart/do_annotation.pl?DOMAIN=LRRCT&TYPE=SMART&START=646&END=698&LENGTH=52&E_VALUE=6.48840098134863e-10&BLAST=NPFDCTCESIAWFVNWINKTRTNISELSSHYLCNTPPQYHGFSVRLFDTSSCK)**18** | 475 | 496 | LQELNVAFNSLTDLPGCGSFSS |
| [**LRR**](http://smart.embl-heidelberg.de/smart/do_annotation.pl?DOMAIN=LRRCT&TYPE=SMART&START=646&END=698&LENGTH=52&E_VALUE=6.48840098134863e-10&BLAST=NPFDCTCESIAWFVNWINKTRTNISELSSHYLCNTPPQYHGFSVRLFDTSSCK)**19** | 497 | 520 | LSVLIIDHNSVSHPSADFFQSCQK |
| [**LRR**](http://smart.embl-heidelberg.de/smart/do_annotation.pl?DOMAIN=LRRCT&TYPE=SMART&START=646&END=698&LENGTH=52&E_VALUE=6.48840098134863e-10&BLAST=NPFDCTCESIAWFVNWINKTRTNISELSSHYLCNTPPQYHGFSVRLFDTSSCK)**20** | 521 | 544 | MRSIKAGDNPFQCTCELREFVKNI |
| **LRR-CT** | 529 | 585 | NPFQCTCELREFVKNIDQVSSEVLEGWPDSYKCDY PESYRGSPLKDFHMSELSCNIT |
| **Transmembrane** | 586 | 608 | LLIVTIGATMLVLAVTVT**S**LC**I**Y |
| **TIR** | 609 | 796 | LDLPWYLRMVCQWTQTRRRARNIPLEELQRNLQFH AFISYSEHDSAWVKSELVPYLEKEDIQICLHERNF VPGKSIVENIINCIEKSYKSIFVLSPNFVQSEWCH YELYFAHHNLFHEGSNNLILILLEPIPQNSIPNKY HKLKALMTQRTYLQWPKEKSKRGLFWANIRAAFNM KLTLVTENNDVK**S** |
