# Supplementary material for: Signatures of positive selection in Toll-like receptor (TLR) genes in mammals
Source: BMC Evol Biol. 2011 Dec 20;11:368. doi: 10.1186/1471-2148-11-368 (PMC3276489; doi:10.1186/1471-2148-11-368)
Supplement: Additional file 27 — Table S27. Domain characterization of TLR7. Microsoft Word document containing the list of domains of Human TLR7 gene, their delimitation and sequence. [file 1471-2148-11-368-S27.DOC]

Table S27. Domain characterization of TLR7.

**The conserved segment of each LRR is underlined. The amino acids identified as under positive selection are in bold.**

| **TLR7 – *Homo sapiens*** | | | |
| --- | --- | --- | --- |
| **Domain** | **Start** | **Stop** | **Sequence** |
| **Signal** | 1 | 26 | MVFPMWTLKRQILILFNIILISKLLG |
| [**LRR**](http://smart.embl-heidelberg.de/smart/do_annotation.pl?DOMAIN=LRR&TYPE=SMART&START=51&END=70&LENGTH=19&E_VALUE=69.0126970495531&BLAST=PTNITVLNLTHNQIKRLPPA)**-NT** | 27 | 66 | ARWFPKTLPCDVTLDVPKNHVIVDCTDKHLTEIPGGIPTN |
| **LRR1** | 67 | 90 | TTNLTLTINHIPDISPASFHRLDH |
| [**LRR**](http://smart.embl-heidelberg.de/smart/do_annotation.pl?DOMAIN=LRR&TYPE=SMART&START=123&END=144&LENGTH=21&E_VALUE=289.551614689825&BLAST=CMNLTELHLMSNSIQKIQNNPF)**2** | 91 | 128 | LVEIDFRCNCVPIPLGSKNNMCIKRLQIKPRSFSGLTY |
| [**LRR**](http://smart.embl-heidelberg.de/smart/do_annotation.pl?DOMAIN=LRR&TYPE=SMART&START=171&END=194&LENGTH=23&E_VALUE=57.8362009479994&BLAST=LQNLQELLLSKNKIQALKSEELAF)**3** | 129 | 149 | LKSLYLDGNQLLEIPQGLPPS |
| [**LRR**](http://smart.embl-heidelberg.de/smart/do_annotation.pl?DOMAIN=LRR&TYPE=SMART&START=197&END=218&LENGTH=21&E_VALUE=384.070417219697&BLAST=NSSLKKLELSSNLIKEFSPGCF)**4** | 150 | 173 | LQLLSLEANNIFSIRKENLTELAN |
| [**LRR**](http://smart.embl-heidelberg.de/smart/do_annotation.pl?DOMAIN=LRR&TYPE=SMART&START=197&END=218&LENGTH=21&E_VALUE=384.070417219697&BLAST=NSSLKKLELSSNLIKEFSPGCF)**5** | 174 | 205 | IEILYLGQNCYYRNPCYVSYSIEKDAFLNLTK |
| [**LRR**](http://smart.embl-heidelberg.de/smart/do_annotation.pl?DOMAIN=LRR&TYPE=SMART&START=274&END=295&LENGTH=21&E_VALUE=6.4745441770878&BLAST=HTNLTMLDLSHNNLNMIDDDSF)**6** | 206 | 226 | LKVLSLKDNNVTAVPTVLPST |
| **LRR7** | 227 | 250 | LTELYLYNNMIAKIQEDDFNNLNQ |
| [**LRR**](http://smart.embl-heidelberg.de/smart/do_annotation.pl?DOMAIN=LRR&TYPE=SMART&START=355&END=378&LENGTH=23&E_VALUE=4.44083621375209&BLAST=LRCLEYLNMEDNDIPSIKRNMFTG)**8** | 251 | 291 | LQILDLSGNCPRCYNAPFPCAPCKNNSPLQIP**V**NAFDALTE |
| [**LRR**](http://smart.embl-heidelberg.de/smart/do_annotation.pl?DOMAIN=LRR&TYPE=SMART&START=379&END=404&LENGTH=25&E_VALUE=87.3274593046497&BLAST=LINLRYLSLSNSFTNLRTLKNETFSS)**9** | 292 | 315 | LKVLRLHSNSLQHVPPRWFKNINK |
| [**LRR**](http://smart.embl-heidelberg.de/smart/do_annotation.pl?DOMAIN=LRR&TYPE=SMART&START=407&END=428&LENGTH=21&E_VALUE=131.25966102461&BLAST=HSPLLILNLTKNKISKIESDAF)**10** | 316 | 341 | LQELDLSQNFLAKEIGDAKFLHFLPS |
| [**LRR**](http://smart.embl-heidelberg.de/smart/do_annotation.pl?DOMAIN=LRR&TYPE=SMART&START=431&END=458&LENGTH=27&E_VALUE=324.191955411346&BLAST=LGSLEVLDIGINEIGQELTGQEWRGLEN)**11** | 342 | 371 | LIQLDLSFNFELQVYRA**S**MNLSQAFSSLKS |
| [**LRR**](http://smart.embl-heidelberg.de/smart/do_annotation.pl?DOMAIN=LRR&TYPE=SMART&START=506&END=524&LENGTH=18&E_VALUE=124.046876494985&BLAST=LHDLTILDLSNNNLANINE)**12** | 372 | 398 | LKILRIRGYVFKEL**K**SFNLSPLHNLQN |
| [**LRR**](http://smart.embl-heidelberg.de/smart/do_annotation.pl?DOMAIN=LRR&TYPE=SMART&START=530&END=564&LENGTH=34&E_VALUE=72.5089815799162&BLAST=LEKLEVLDLQHNNLARLWKQANPGGPVHFLKGLSH)**13** | 399 | 422 | LEVLDLGTNFIKIANLSMFKQFKR |
| **LRR14** | 423 | 494 | LKVIDLSVNKISPSGDSSEVGFCSNARTSVESYEPQVLEQLHYFRYDKYARSCRFKNKEASFMSVNESCYKY |
| [**LRR**](http://smart.embl-heidelberg.de/smart/do_annotation.pl?DOMAIN=LRR&TYPE=SMART&START=586&END=605&LENGTH=19&E_VALUE=520.428720428041&BLAST=LFQLKSINLALNNLNVLPQS)**15** | 495 | 518 | GQTLDLSKNSIFFVKSSDFQHLSF |
| [**LRR**](http://smart.embl-heidelberg.de/smart/do_annotation.pl?DOMAIN=LRR&TYPE=SMART&START=611&END=633&LENGTH=22&E_VALUE=25.3611539551777&BLAST=VSLKSLNLQKNLITSVEKKVFGP)**16** | 519 | 543 | LKCLNLSGNLISQTLNGSEFQPLAE |
| [**LRR**](http://smart.embl-heidelberg.de/smart/do_annotation.pl?DOMAIN=LRRCT&TYPE=SMART&START=646&END=698&LENGTH=52&E_VALUE=6.48840098134863e-10&BLAST=NPFDCTCESIAWFVNWINKTRTNISELSSHYLCNTPPQYHGFSVRLFDTSSCK)**17** | 544 | 567 | LRYLDFSNNRLDLLHSTAFEELHK |
| [**LRR**](http://smart.embl-heidelberg.de/smart/do_annotation.pl?DOMAIN=LRRCT&TYPE=SMART&START=646&END=698&LENGTH=52&E_VALUE=6.48840098134863e-10&BLAST=NPFDCTCESIAWFVNWINKTRTNISELSSHYLCNTPPQYHGFSVRLFDTSSCK)**18** | 568 | 597 | LEVLDISSNSHYFQSEGITHMLNFTKNLKV |
| [**LRR**](http://smart.embl-heidelberg.de/smart/do_annotation.pl?DOMAIN=LRRCT&TYPE=SMART&START=646&END=698&LENGTH=52&E_VALUE=6.48840098134863e-10&BLAST=NPFDCTCESIAWFVNWINKTRTNISELSSHYLCNTPPQYHGFSVRLFDTSSCK)**19** | 598 | 620 | L**Q**KLMMNDNDISSSTSRTMESES |
| [**LRR**](http://smart.embl-heidelberg.de/smart/do_annotation.pl?DOMAIN=LRRCT&TYPE=SMART&START=646&END=698&LENGTH=52&E_VALUE=6.48840098134863e-10&BLAST=NPFDCTCESIAWFVNWINKTRTNISELSSHYLCNTPPQYHGFSVRLFDTSSCK)**20** | 621 | 651 | LRTLEFRGNHLDVLWREGDNRYLQLFKNLLK |
| **LRR21** | 652 | 676 | LEELDISKNSLSFLP**S**GVFDGMPPN |
| **LRR22** | 677 | 700 | LKNLSLAKNGLKSFSWKKLQ**C**LKN |
| **LRR23** | 701 | 724 | LETLDLSHNQLTTVPERLSNCSRS |
| **LRR24** | 725 | 748 | LKNLILKNNQIRSLTKYFLQDAFQ |
| **LRR25** | 749 | 774 | LRYLDLSSNKIQMIQKTSFPENVLNN |
| **LRR26** | 775 | 798 | L**K**MLLLHHNRFLCTCDAVWFVWWV |
| **LRR-CT** | 783 | 840 | NRFLCTCDAVWFVWWVNHTEVTIPYLATDVTCVGPGAHKGQSVISLDLYTCELDLTNL |
| **Transmembrane** | 841 | 863 | ILFSLSISVSLFLMVMMTASHLY |
| **TIR** | 864 | 1049 | FWDVWYIYHFCKAKIKGYQRLISPDCCYDAFIVYDTKDPAVTEWVLAELVAKLEDPREKHFNLCLEERDWLPGQPVLENLSQSIQLSKKTVFVMTDKYAKTENFKIAFYLSHQRLMDEKVDVIILIFLEKPFQKSKFLQLRKRLCGSSVLEWPTNPQAHPYFWQCLKNALATDNHVAYSQVFKETV |
