# Supplementary material for: Signatures of positive selection in Toll-like receptor (TLR) genes in mammals
Source: BMC Evol Biol. 2011 Dec 20;11:368. doi: 10.1186/1471-2148-11-368 (PMC3276489; doi:10.1186/1471-2148-11-368)
Supplement: Additional file 28 — Table S28. Domain characterization of TLR8. Microsoft Word document containing the list of domains of Human TLR8 gene, their delimitation and sequence. [file 1471-2148-11-368-S28.DOC]

Table S28. Domain characterization of TLR8.

**The conserved segment of each LRR is underlined. The amino acids identified as under positive selection are in bold.**

| **TLR8 – *Homo sapiens*** | | | |
| --- | --- | --- | --- |
| **Domain** | **Start** | **Stop** | **Sequence** |
| **Signal** | 1 | 28 | MENMFLQSSMLTCIFLLISGSCELCAEE |
| [**LRR**](http://smart.embl-heidelberg.de/smart/do_annotation.pl?DOMAIN=LRR&TYPE=SMART&START=51&END=70&LENGTH=19&E_VALUE=69.0126970495531&BLAST=PTNITVLNLTHNQIKRLPPA)**-NT** | 29 | 64 | NFSRSYPCDE**K**KQNDSVIAECSNRRLQEVPQTVGKY |
| **LRR1** | 65 | 88 | VTELDLSDNFITHITNESFQGLQN |
| [**LRR**](http://smart.embl-heidelberg.de/smart/do_annotation.pl?DOMAIN=LRR&TYPE=SMART&START=123&END=144&LENGTH=21&E_VALUE=289.551614689825&BLAST=CMNLTELHLMSNSIQKIQNNPF)**2** | 89 | 126 | LTKINLNHNPNVQHQNGNPGIQSNGLNITDGAFLNLKN |
| [**LRR**](http://smart.embl-heidelberg.de/smart/do_annotation.pl?DOMAIN=LRR&TYPE=SMART&START=171&END=194&LENGTH=23&E_VALUE=57.8362009479994&BLAST=LQNLQELLLSKNKIQALKSEELAF)**3** | 127 | 147 | LRELLLEDNQLPQIPSGLP**E**S |
| [**LRR**](http://smart.embl-heidelberg.de/smart/do_annotation.pl?DOMAIN=LRR&TYPE=SMART&START=197&END=218&LENGTH=21&E_VALUE=384.070417219697&BLAST=NSSLKKLELSSNLIKEFSPGCF)**4** | 148 | 171 | LTELSLIQNNIY**N**ITKEGISRLIN |
| [**LRR**](http://smart.embl-heidelberg.de/smart/do_annotation.pl?DOMAIN=LRR&TYPE=SMART&START=197&END=218&LENGTH=21&E_VALUE=384.070417219697&BLAST=NSSLKKLELSSNLIKEFSPGCF)**5** | 172 | 202 | LK**N**LYLAWNCYFNK**V**CEKT**N**IEDGVFETLTN |
| [**LRR**](http://smart.embl-heidelberg.de/smart/do_annotation.pl?DOMAIN=LRR&TYPE=SMART&START=274&END=295&LENGTH=21&E_VALUE=6.4745441770878&BLAST=HTNLTMLDLSHNNLNMIDDDSF)**6** | 203 | 223 | LELLSLSFNSL**S**HVPPKLPSS |
| **LRR7** | 224 | 247 | LRKLFLSNTQIK**Y**ISEEDFKGLIN |
| [**LRR**](http://smart.embl-heidelberg.de/smart/do_annotation.pl?DOMAIN=LRR&TYPE=SMART&START=355&END=378&LENGTH=23&E_VALUE=4.44083621375209&BLAST=LRCLEYLNMEDNDIPSIKRNMFTG)**8** | 248 | 288 | L**T**LLDLSGNCPRCFNAPFPCVPCDGGASINIDRFAFQNLTQ |
| [**LRR**](http://smart.embl-heidelberg.de/smart/do_annotation.pl?DOMAIN=LRR&TYPE=SMART&START=379&END=404&LENGTH=25&E_VALUE=87.3274593046497&BLAST=LINLRYLSLSNSFTNLRTLKNETFSS)**9** | 289 | 312 | LRYLNLSSTSLRKINAAWFKNMPH |
| [**LRR**](http://smart.embl-heidelberg.de/smart/do_annotation.pl?DOMAIN=LRR&TYPE=SMART&START=407&END=428&LENGTH=21&E_VALUE=131.25966102461&BLAST=HSPLLILNLTKNKISKIESDAF)**10** | 313 | 338 | LKVLDLEFNYLVGEIASGAFLTMLP**R** |
| [**LRR**](http://smart.embl-heidelberg.de/smart/do_annotation.pl?DOMAIN=LRR&TYPE=SMART&START=431&END=458&LENGTH=27&E_VALUE=324.191955411346&BLAST=LGSLEVLDIGINEIGQELTGQEWRGLEN)**11** | 339 | 368 | LEILDLSFNY**I**KGSYPQHINISRNFSKLLS |
| [**LRR**](http://smart.embl-heidelberg.de/smart/do_annotation.pl?DOMAIN=LRR&TYPE=SMART&START=506&END=524&LENGTH=18&E_VALUE=124.046876494985&BLAST=LHDLTILDLSNNNLANINE)**12** | 369 | 395 | LRALHLRGYVFQELREDDF**Q**PLMQLPN |
| [**LRR**](http://smart.embl-heidelberg.de/smart/do_annotation.pl?DOMAIN=LRR&TYPE=SMART&START=530&END=564&LENGTH=34&E_VALUE=72.5089815799162&BLAST=LEKLEVLDLQHNNLARLWKQANPGGPVHFLKGLSH)**13** | 396 | 419 | LSTINLGINFIKQIDFKLFQ**N**F**S**N |
| **LRR14** | 420 | 482 | LEIIYLSENRISPLVKDTRQS**Y**ANSSSFQRHIRKRRSTD**F**EFDPHSNFYHFT**R**PLIKPQCA**A**Y |
| [**LRR**](http://smart.embl-heidelberg.de/smart/do_annotation.pl?DOMAIN=LRR&TYPE=SMART&START=586&END=605&LENGTH=19&E_VALUE=520.428720428041&BLAST=LFQLKSINLALNNLNVLPQS)**15** | 483 | 506 | GKALDLSLNSIFFIG**P**NQFENLPD |
| [**LRR**](http://smart.embl-heidelberg.de/smart/do_annotation.pl?DOMAIN=LRR&TYPE=SMART&START=611&END=633&LENGTH=22&E_VALUE=25.3611539551777&BLAST=VSLKSLNLQKNLITSVEKKVFGP)**16** | 507 | 531 | IACLNLSANSNAQVLSGTEFSAIPH |
| [**LRR**](http://smart.embl-heidelberg.de/smart/do_annotation.pl?DOMAIN=LRRCT&TYPE=SMART&START=646&END=698&LENGTH=52&E_VALUE=6.48840098134863e-10&BLAST=NPFDCTCESIAWFVNWINKTRTNISELSSHYLCNTPPQYHGFSVRLFDTSSCK)**17** | 532 | 555 | VKYLDLTNNRLDFDNASALTELSD |
| [**LRR**](http://smart.embl-heidelberg.de/smart/do_annotation.pl?DOMAIN=LRRCT&TYPE=SMART&START=646&END=698&LENGTH=52&E_VALUE=6.48840098134863e-10&BLAST=NPFDCTCESIAWFVNWINKTRTNISELSSHYLCNTPPQYHGFSVRLFDTSSCK)**18** | 556 | 585 | LEVLDLSYNSHYFRIAGVTHHLEFIQNFTN |
| [**LRR**](http://smart.embl-heidelberg.de/smart/do_annotation.pl?DOMAIN=LRRCT&TYPE=SMART&START=646&END=698&LENGTH=52&E_VALUE=6.48840098134863e-10&BLAST=NPFDCTCESIAWFVNWINKTRTNISELSSHYLCNTPPQYHGFSVRLFDTSSCK)**19** | 586 | 609 | LKVLNLSHNNIYTLTDKYNL**E**SKS |
| [**LRR**](http://smart.embl-heidelberg.de/smart/do_annotation.pl?DOMAIN=LRRCT&TYPE=SMART&START=646&END=698&LENGTH=52&E_VALUE=6.48840098134863e-10&BLAST=NPFDCTCESIAWFVNWINKTRTNISELSSHYLCNTPPQYHGFSVRLFDTSSCK)**20** | 610 | 640 | LVELVFSGNRLDILWNDDD**N**RYI**S**IFKGL**K**N |
| **LRR21** | 641 | 665 | LTRLDLSLNRLKHIPNEAFLNLPAS |
| **LRR22** | 666 | 689 | LTELHINDNMLKFFNWTLLQQFPR |
| **LRR23** | 690 | 713 | LELLDLRGNKLLFLTDSLSDFT**S**S |
| **LRR24** | 714 | 737 | LRTLLLSHNRISHLPSGFLSEVSS |
| **LRR25** | 738 | 763 | LKHLDLSSNLLKTINKSALETKT**T**TK |
| **LRR26** | 764 | 787 | LS**M**LELHGNPFECTCDIGDFRRWM |
| **LRR-CT** | 772 | 828 | NPFECTCDIGDFRRWMDEHLNVKIPRLVDVICASPGDQRGKSIVSLELTTCVSDVTA |
| **Transmembrane** | 829 | 851 | VILFFFTFFITTMVMLAALAHHL |
| **TIR** | 852 | 1041 | FYWDVWFIYNVCLAKVKGYRSLSTSQTFYDAYISYDTKDASVTDWVINELRYHLEESRDKNVLLCLEERDWDPGLAIIDNLMQSINQSKKTVFVLTKKYAKSWNFKTAFYLALQRLMDENMDVIIFILLEPVLQHSQYLRLRQRICKSSILQWPDNPKAEGLFWQTLRNVVLTENDSRYNNMYVDSIKQY |
