# Supplementary material for: Signatures of positive selection in Toll-like receptor (TLR) genes in mammals
Source: BMC Evol Biol. 2011 Dec 20;11:368. doi: 10.1186/1471-2148-11-368 (PMC3276489; doi:10.1186/1471-2148-11-368)
Supplement: Additional file 29 — Table S29. Domain characterization of TLR9. Microsoft Word document containing the list of domains of Human TLR9 gene, their delimitation and sequence. [file 1471-2148-11-368-S29.DOC]

Table S29. Domain characterization of TLR9.

**The conserved segment of each LRR is underlined. The amino acids identified as under positive selection are in bold.**

| **TLR9 – *Homo sapiens*** | | | |
| --- | --- | --- | --- |
| **Domain** | **Start** | **Stop** | **Sequence** |
| **Signal** | 1 | 25 | MGFCRSALHPLSLLVQAIMLAMTLA |
| [**LRR**](http://smart.embl-heidelberg.de/smart/do_annotation.pl?DOMAIN=LRR&TYPE=SMART&START=51&END=70&LENGTH=19&E_VALUE=69.0126970495531&BLAST=PTNITVLNLTHNQIKRLPPA)**-NT** | 26 | 64 | LGTLPAFLPCELQPHGLVNCNWLFLKSVPHFSMAAPRGN |
| **LRR1** | 65 | 88 | VTSLSL**S**SNRIHHLHDSDFAHLPS |
| [**LRR**](http://smart.embl-heidelberg.de/smart/do_annotation.pl?DOMAIN=LRR&TYPE=SMART&START=123&END=144&LENGTH=21&E_VALUE=289.551614689825&BLAST=CMNLTELHLMSNSIQKIQNNPF)**2** | 89 | 124 | LRHLNLKWNCPPVGLSPMHFPCHMTIEPSTFLAVPT |
| [**LRR**](http://smart.embl-heidelberg.de/smart/do_annotation.pl?DOMAIN=LRR&TYPE=SMART&START=171&END=194&LENGTH=23&E_VALUE=57.8362009479994&BLAST=LQNLQELLLSKNKIQALKSEELAF)**3** | 125 | 144 | LEELNLSYNNIMTVPALPKS |
| [**LRR**](http://smart.embl-heidelberg.de/smart/do_annotation.pl?DOMAIN=LRR&TYPE=SMART&START=197&END=218&LENGTH=21&E_VALUE=384.070417219697&BLAST=NSSLKKLELSSNLIKEFSPGCF)**4** | 145 | 168 | LISLSLSHTNILMLDS**A**SLAGLHA |
| [**LRR**](http://smart.embl-heidelberg.de/smart/do_annotation.pl?DOMAIN=LRR&TYPE=SMART&START=197&END=218&LENGTH=21&E_VALUE=384.070417219697&BLAST=NSSLKKLELSSNLIKEFSPGCF)**5** | 169 | 200 | LRFLFMDGNCYYKNPCRQALEVAPGALLGLGN |
| [**LRR**](http://smart.embl-heidelberg.de/smart/do_annotation.pl?DOMAIN=LRR&TYPE=SMART&START=274&END=295&LENGTH=21&E_VALUE=6.4745441770878&BLAST=HTNLTMLDLSHNNLNMIDDDSF)**6** | 201 | 221 | LTHLSLKYNNLTVVPR**N**LPSS |
| **LRR7** | 222 | 245 | LEYLLLSYNRIVKLAPEDLANLTA |
| [**LRR**](http://smart.embl-heidelberg.de/smart/do_annotation.pl?DOMAIN=LRR&TYPE=SMART&START=355&END=378&LENGTH=23&E_VALUE=4.44083621375209&BLAST=LRCLEYLNMEDNDIPSIKRNMFTG)**8** | 246 | 285 | LRVLDVGGNCRRCDHAPNPCMECPRHFPQLHPDTFSHLSR |
| [**LRR**](http://smart.embl-heidelberg.de/smart/do_annotation.pl?DOMAIN=LRR&TYPE=SMART&START=379&END=404&LENGTH=25&E_VALUE=87.3274593046497&BLAST=LINLRYLSLSNSFTNLRTLKNETFSS)**9** | 286 | 309 | LEGLVLKDSSLSWLNASWFRGLGN |
| [**LRR**](http://smart.embl-heidelberg.de/smart/do_annotation.pl?DOMAIN=LRR&TYPE=SMART&START=407&END=428&LENGTH=21&E_VALUE=131.25966102461&BLAST=HSPLLILNLTKNKISKIESDAF)**10** | 310 | 335 | LRVLDLSENFLYKCITKTKAFQ**G**LTQ |
| [**LRR**](http://smart.embl-heidelberg.de/smart/do_annotation.pl?DOMAIN=LRR&TYPE=SMART&START=431&END=458&LENGTH=27&E_VALUE=324.191955411346&BLAST=LGSLEVLDIGINEIGQELTGQEWRGLEN)**11** | 336 | 365 | LRKLNLSFNYQKRVSFAHLSLAPSFGSLVA |
| [**LRR**](http://smart.embl-heidelberg.de/smart/do_annotation.pl?DOMAIN=LRR&TYPE=SMART&START=506&END=524&LENGTH=18&E_VALUE=124.046876494985&BLAST=LHDLTILDLSNNNLANINE)**12** | 366 | 392 | LKELDMHGIFFRSLDETTLRPLARLPM |
| [**LRR**](http://smart.embl-heidelberg.de/smart/do_annotation.pl?DOMAIN=LRR&TYPE=SMART&START=530&END=564&LENGTH=34&E_VALUE=72.5089815799162&BLAST=LEKLEVLDLQHNNLARLWKQANPGGPVHFLKGLSH)**13** | 393 | 416 | LQTLRLQMNFINQAQLGIFRAFPG |
| **LRR14** | 17 | 473 | LRYVDLSDNRISGASELTATMGEADGGEKVWLQPGDLAPAPVDTPSSEDFRPNCSTL |
| [**LRR**](http://smart.embl-heidelberg.de/smart/do_annotation.pl?DOMAIN=LRR&TYPE=SMART&START=586&END=605&LENGTH=19&E_VALUE=520.428720428041&BLAST=LFQLKSINLALNNLNVLPQS)**15** | 474 | 497 | NFTLDLSRNNLVTVQPEMFAQLSH |
| [**LRR**](http://smart.embl-heidelberg.de/smart/do_annotation.pl?DOMAIN=LRR&TYPE=SMART&START=611&END=633&LENGTH=22&E_VALUE=25.3611539551777&BLAST=VSLKSLNLQKNLITSVEKKVFGP)**16** | 498 | 522 | LQCLRLSHNCISQAVNGSQFLPLTG |
| [**LRR**](http://smart.embl-heidelberg.de/smart/do_annotation.pl?DOMAIN=LRRCT&TYPE=SMART&START=646&END=698&LENGTH=52&E_VALUE=6.48840098134863e-10&BLAST=NPFDCTCESIAWFVNWINKTRTNISELSSHYLCNTPPQYHGFSVRLFDTSSCK)**17** | 523 | 546 | LQVLDLSHNKLDLYHEHSFTELPR |
| [**LRR**](http://smart.embl-heidelberg.de/smart/do_annotation.pl?DOMAIN=LRRCT&TYPE=SMART&START=646&END=698&LENGTH=52&E_VALUE=6.48840098134863e-10&BLAST=NPFDCTCESIAWFVNWINKTRTNISELSSHYLCNTPPQYHGFSVRLFDTSSCK)**18** | 547 | 576 | LEALDLSYNSQPFGMQGVGHNFSFVAHLRT |
| [**LRR**](http://smart.embl-heidelberg.de/smart/do_annotation.pl?DOMAIN=LRRCT&TYPE=SMART&START=646&END=698&LENGTH=52&E_VALUE=6.48840098134863e-10&BLAST=NPFDCTCESIAWFVNWINKTRTNISELSSHYLCNTPPQYHGFSVRLFDTSSCK)**19** | 577 | 599 | LRHLSLAHNNIHSQVSQQLCSTS |
| [**LRR**](http://smart.embl-heidelberg.de/smart/do_annotation.pl?DOMAIN=LRRCT&TYPE=SMART&START=646&END=698&LENGTH=52&E_VALUE=6.48840098134863e-10&BLAST=NPFDCTCESIAWFVNWINKTRTNISELSSHYLCNTPPQYHGFSVRLFDTSSCK)**20** | 600 | 629 | LRALDFSGNALGHMWAEGDLYLHFFQGLSG |
| **LRR21** | 630 | 654 | LIWLDLSQNRLHTLLPQTLRNLPKS |
| **LRR22** | 655 | 678 | LQVLRLRDNYLAFFKWWSLHFLPK |
| **LRR23** | 679 | 702 | LEVLDLAGNQLKALTNGSLPAGTR |
| **LRR24** | 703 | 726 | LRRLDVSCNSISFVAPGFFSKAKE |
| **LRR25** | 727 | 751 | LRELNLSANALKTVDHSWFGPLASA |
| **LRR26** | 752 | 775 | LQILDVSANPLHCACGAAFMDFLL |
| **LRR-CT** | 760 | 812 | NPLHCACGAAFMDFLLEVQAAVPGLPSRVKCGSPGQLQGLSIFAQDLRLCLDE |
| **Transmembrane** | 813 | 835 | ALSWDCFALSLLAVALGLGVPML |
| **TIR** | 836 | 1032 | HHLCGWDLWYCFHLCLAWLPWRGRQSGRDEDALPYDAFVVFDKTQSAVADWVYNELRGQLEECRGRWALRLCLEERDWLPGKTLFENLWASVYGSRKTLFVLAHTDRVSGLLRASFLLAQQRLLEDRKDVVVLVILSPDG RRSRYVRLRQRLCRQSVLLWPHQPSGQRSFWAQLGMALTRDNHHFYNRNFCQGPTAE |
