# Supplementary material for: Signatures of positive selection in Toll-like receptor (TLR) genes in mammals
Source: BMC Evol Biol. 2011 Dec 20;11:368. doi: 10.1186/1471-2148-11-368 (PMC3276489; doi:10.1186/1471-2148-11-368)
Supplement: Additional file 30 — Table S30. Domain characterization of TLR10. Microsoft Word document containing the list of domains of Human TLR10 gene, their delimitation and sequence. [file 1471-2148-11-368-S30.DOC]

Table S30. Domain characterization of TLR10.

**The conserved segment of each LRR is underlined. The amino acids identified as under positive selection are in bold.**

| **TLR10 – *Homo sapiens*** | | | |
| --- | --- | --- | --- |
| **Domain** | **Start** | **Stop** | **Sequence** |
| **Signal** | 1 | 19 | MRLIRNIYIFCSIVMTAEG |
| [**LRR**](http://smart.embl-heidelberg.de/smart/do_annotation.pl?DOMAIN=LRR&TYPE=SMART&START=51&END=70&LENGTH=19&E_VALUE=69.0126970495531&BLAST=PTNITVLNLTHNQIKRLPPA)**-NT** | 20 | 49 | DAPELPEERELMTNCSNMSLRKVPADLTPA |
| **LRR1** | 50 | 73 | TTTLDLSYNLLFQLQSSDFHSVSK |
| [**LRR**](http://smart.embl-heidelberg.de/smart/do_annotation.pl?DOMAIN=LRR&TYPE=SMART&START=123&END=144&LENGTH=21&E_VALUE=289.551614689825&BLAST=CMNLTELHLMSNSIQKIQNNPF)**2** | 74 | 97 | LRVLILCHNRIQQLDLK**T**FEFNKE |
| [**LRR**](http://smart.embl-heidelberg.de/smart/do_annotation.pl?DOMAIN=LRR&TYPE=SMART&START=171&END=194&LENGTH=23&E_VALUE=57.8362009479994&BLAST=LQNLQELLLSKNKIQALKSEELAF)**3** | 98 | 118 | LRYLDLSNNRLKSVTWYLLAG |
| [**LRR**](http://smart.embl-heidelberg.de/smart/do_annotation.pl?DOMAIN=LRR&TYPE=SMART&START=197&END=218&LENGTH=21&E_VALUE=384.070417219697&BLAST=NSSLKKLELSSNLIKEFSPGCF)**4** | 119 | 143 | LRYLDLSFNDFDTMPICEEAGNMSH |
| [**LRR**](http://smart.embl-heidelberg.de/smart/do_annotation.pl?DOMAIN=LRR&TYPE=SMART&START=197&END=218&LENGTH=21&E_VALUE=384.070417219697&BLAST=NSSLKKLELSSNLIKEFSPGCF)**5** | 144 | 166 | LEILGLSGAKIQKSDFQKIAHLH |
| [**LRR**](http://smart.embl-heidelberg.de/smart/do_annotation.pl?DOMAIN=LRR&TYPE=SMART&START=274&END=295&LENGTH=21&E_VALUE=6.4745441770878&BLAST=HTNLTMLDLSHNNLNMIDDDSF)**6** | 167 | 192 | LNTVFLGFRTLPHYEEGSLPILNTTK |
| **LRR7** | 193 | 216 | LHIVLPMDTNFWVLLRDGIKTSKI |
| [**LRR**](http://smart.embl-heidelberg.de/smart/do_annotation.pl?DOMAIN=LRR&TYPE=SMART&START=355&END=378&LENGTH=23&E_VALUE=4.44083621375209&BLAST=LRCLEYLNMEDNDIPSIKRNMFTG)**8** | 217 | 243 | LEMTNIDGKSQFVSYEMQRNL**S**LENAK |
| [**LRR**](http://smart.embl-heidelberg.de/smart/do_annotation.pl?DOMAIN=LRR&TYPE=SMART&START=379&END=404&LENGTH=25&E_VALUE=87.3274593046497&BLAST=LINLRYLSLSNSFTNLRTLKNETFSS)**9** | 244 | 281 | TSVLLLNKVDLLWDDLFLILQFVWHTSVEHFQIRNVTF |
| [**LRR**](http://smart.embl-heidelberg.de/smart/do_annotation.pl?DOMAIN=LRR&TYPE=SMART&START=407&END=428&LENGTH=21&E_VALUE=131.25966102461&BLAST=HSPLLILNLTKNKISKIESDAF)**10** | 282 | 303 | GGKAYLDHNSFDYSNTVMRTIK |
| [**LRR**](http://smart.embl-heidelberg.de/smart/do_annotation.pl?DOMAIN=LRR&TYPE=SMART&START=431&END=458&LENGTH=27&E_VALUE=324.191955411346&BLAST=LGSLEVLDIGINEIGQELTGQEWRGLEN)**11** | 304 | 327 | LEHVHFRVFYIQQDKIYLLLTKMD |
| [**LRR**](http://smart.embl-heidelberg.de/smart/do_annotation.pl?DOMAIN=LRR&TYPE=SMART&START=506&END=524&LENGTH=18&E_VALUE=124.046876494985&BLAST=LHDLTILDLSNNNLANINE)**12** | 328 | 349 | IENLTISNAQMPHMLFPNYPTK |
| [**LRR**](http://smart.embl-heidelberg.de/smart/do_annotation.pl?DOMAIN=LRR&TYPE=SMART&START=530&END=564&LENGTH=34&E_VALUE=72.5089815799162&BLAST=LEKLEVLDLQHNNLARLWKQANPGGPVHFLKGLSH)**13** | 350 | 373 | FQYLNFANNILTDELFKRTIQLPH |
| **LRR14** | 374 | 398 | LKTLILNGNKLETLSLVS**C**FANNTP |
| [**LRR**](http://smart.embl-heidelberg.de/smart/do_annotation.pl?DOMAIN=LRR&TYPE=SMART&START=586&END=605&LENGTH=19&E_VALUE=520.428720428041&BLAST=LFQLKSINLALNNLNVLPQS)**15** | 399 | 422 | LEHLDLSQNLLQHKNDENCSWPET |
| [**LRR**](http://smart.embl-heidelberg.de/smart/do_annotation.pl?DOMAIN=LRR&TYPE=SMART&START=611&END=633&LENGTH=22&E_VALUE=25.3611539551777&BLAST=VSLKSLNLQKNLITSVEKKVFGP)**16** | 423 | 444 | VVNMNLSYNKLSDSVFRCLPKS |
| [**LRR**](http://smart.embl-heidelberg.de/smart/do_annotation.pl?DOMAIN=LRRCT&TYPE=SMART&START=646&END=698&LENGTH=52&E_VALUE=6.48840098134863e-10&BLAST=NPFDCTCESIAWFVNWINKTRTNISELSSHYLCNTPPQYHGFSVRLFDTSSCK)**17** | 445 | 467 | IQILDLNNNQIQTVPKETIHLMA |
| [**LRR**](http://smart.embl-heidelberg.de/smart/do_annotation.pl?DOMAIN=LRRCT&TYPE=SMART&START=646&END=698&LENGTH=52&E_VALUE=6.48840098134863e-10&BLAST=NPFDCTCESIAWFVNWINKTRTNISELSSHYLCNTPPQYHGFSVRLFDTSSCK)**18** | 468 | 489 | L**R**ELNIAFNFLTDLPGCSHFSR |
| [**LRR**](http://smart.embl-heidelberg.de/smart/do_annotation.pl?DOMAIN=LRRCT&TYPE=SMART&START=646&END=698&LENGTH=52&E_VALUE=6.48840098134863e-10&BLAST=NPFDCTCESIAWFVNWINKTRTNISELSSHYLCNTPPQYHGFSVRLFDTSSCK)**19** | 490 | 513 | LS**V**LNIEMNFILSPSLDFVQSCQE |
| [**LRR**](http://smart.embl-heidelberg.de/smart/do_annotation.pl?DOMAIN=LRRCT&TYPE=SMART&START=646&END=698&LENGTH=52&E_VALUE=6.48840098134863e-10&BLAST=NPFDCTCESIAWFVNWINKTRTNISELSSHYLCNTPPQYHGFSVRLFDTSSCK)**20** | 514 | 537 | VKTLNAGRNPFRCTCELKNFIQLE |
| **LRR-CT** | 522 | 577 | NPFRCTCELKNFIQLETYSEVMM**V**GWSDSYTCEYP LNLRGTRLKDVHLHELSCNTA |
| **Transmembrane** | 578 | 600 | LLIVTIVVIMLVLGLAVAFCCLH |
| **TIR** | 601 | 811 | FDLPWYLRMLGQCTQTWHRVRKTTQEQLKRNVRFHAFISYSEHDSLWVKNELIPNLEKEDGSILICLYESYFDPGKSISENIVSFIEKSYKSIFVLSPNFVQNEWCHYEFYFAHHNLFHENSDHIILILLEPIPFYCIPTRYHKLKALLEKKAYLEWPKDRRKCGLFWANLRAAINVNVLATREMYELQTFTELNEESRGST**I**SLMRTDCL |
