# Supplementary material for: Signatures of positive selection in Toll-like receptor (TLR) genes in mammals
Source: BMC Evol Biol. 2011 Dec 20;11:368. doi: 10.1186/1471-2148-11-368 (PMC3276489; doi:10.1186/1471-2148-11-368)
Supplement: Additional file 31 — Table S31. Characterization of the amino acids possibilities for each residue identified under positive selection in TLR1. Microsoft Word document containing the list of PSC in TLR1 and their localization in domains of Human TLR1 gene. For each site, we list the amino acid possibilities and the characterization of their polarity and charge. [file 1471-2148-11-368-S31.DOC]

**Table S31. Characterization of the amino acids possibilities for each residue identified under positive selection in TLR1.**

| **TLR1** | | | | |
| --- | --- | --- | --- | --- |
| **Position** | **Domain** | **Amino acid possibilities** | **Polarity** | **Charge** |
| **174** | **LRR6** | T | Hydrophilic | Neutral |
|  |  | A | Hydrophobic | Neutral |
|  |  | H | Hydrophilic | Positive |
|  |  | P | Hydrophilic | Neutral |
|  |  | F | Hydrophobic | Neutral |
|  |  | S | Hydrophilic | Neutral |
| **293** | **LRR10** | D | Hydrophilic | Negative |
|  |  | N | Hydrophilic | Neutral |
|  |  | G | Hydrophilic | Neutral |
|  |  | H | Hydrophilic | Positive |
