# Supplementary material for: Signatures of positive selection in Toll-like receptor (TLR) genes in mammals
Source: BMC Evol Biol. 2011 Dec 20;11:368. doi: 10.1186/1471-2148-11-368 (PMC3276489; doi:10.1186/1471-2148-11-368)
Supplement: Additional file 35 — Table S35. Characterization of the amino acids possibilities for each residue identified under positive selection in TLR5. Microsoft Word document containing the list of PSC in TLR5 and their localization in domains of Human TLR5 gene. For each site, we list the amino acid possibilities and the characterization of their polarity and charge. [file 1471-2148-11-368-S35.DOC]

**Table S35. Characterization of the amino acids possibilities for each residue identified under positive selection in TLR5.**

| **TLR5** | | | | |
| --- | --- | --- | --- | --- |
| **Position** | **Domain** | **Amino acid possibilities** | **Polarity** | **Charge** |
| **14** | **Signal** | M | Hydrophobic | Neutral |
|  |  | V | Hydrophobic | Neutral |
|  |  | L | Hydrophobic | Neutral |
|  |  | G | Hydrophilic | Neutral |
|  |  | A | Hydrophobic | Neutral |
|  |  | K | Hydrophilic | Positive |
| **128** | **LRR4** | F | Hydrophobic | Neutral |
|  |  | S | Hydrophilic | Neutral |
|  |  | A | Hydrophobic | Neutral |
|  |  | Y | Hydrophobic | Neutral |
|  |  | G | Hydrophilic | Neutral |
|  |  | D | Hydrophilic | Negative |
|  |  | Q | Hydrophilic | Neutral |
| **207** | **LRR7** | S | Hydrophilic | Neutral |
|  |  | N | Hydrophilic | Neutral |
|  |  | Q | Hydrophilic | Neutral |
|  |  | H | Hydrophilic | Positive |
|  |  | G | Hydrophilic | Neutral |
|  |  | K | Hydrophilic | Positive |
|  |  | T | Hydrophilic | Neutral |
| **400** | **LRR14** | H | Hydrophilic | Positive |
|  |  | Y | Hydrophobic | Neutral |
|  |  | N | Hydrophilic | Neutral |
|  |  | S | Hydrophilic | Neutral |
|  |  | G | Hydrophilic | Neutral |
|  |  | Q | Hydrophilic | Neutral |
|  |  | P | Hydrophilic | Neutral |
| **674** | **TIR** | A | Hydrophobic | Neutral |
|  |  | V | Hydrophobic | Neutral |
|  |  | I | Hydrophobic | Neutral |
|  |  | F | Hydrophobic | Neutral |
| **721** | **TIR** | D | Hydrophilic | Negative |
|  |  | S | Hydrophilic | Neutral |
|  |  | T | Hydrophilic | Neutral |
|  |  | N | Hydrophilic | Neutral |
|  |  | P | Hydrophilic | Neutral |
| **742** | **TIR** | A | Hydrophobic | Neutral |
|  |  | S | Hydrophilic | Neutral |
|  |  | V | Hydrophobic | Neutral |
|  |  | T | Hydrophilic | Neutral |
|  |  | N | Hydrophilic | Neutral |
