# Supplementary material for: Signatures of positive selection in Toll-like receptor (TLR) genes in mammals
Source: BMC Evol Biol. 2011 Dec 20;11:368. doi: 10.1186/1471-2148-11-368 (PMC3276489; doi:10.1186/1471-2148-11-368)
Supplement: Additional file 38 — Table S38. Characterization of the amino acids possibilities for each residue identified under positive selection in TLR8. Microsoft Word document containing the list of PSC in TLR8 and their localization in domains of Human TLR8 gene. For each site, we list the amino acid possibilities and the characterization of their polarity and charge. [file 1471-2148-11-368-S38.DOC]

**Table S38. Characterization of the amino acids possibilities for each residue identified under positive selection in TLR8.**

| **TLR8** | | | | |
| --- | --- | --- | --- | --- |
| **Position** | **Domain** | **Amino acid possibilities** | **Polarity** | **Charge** |
| **39** | **LRR-NT** | K | Hydrophilic | Positive |
|  |  | I | Hydrophobic | Neutral |
|  |  | R | Hydrophilic | Positive |
|  |  | S | Hydrophilic | Neutral |
|  |  | T | Hydrophilic | Neutral |
| **146** | **LRR3** | E | Hydrophilic | Negative |
|  |  | G | Hydrophilic | Neutral |
|  |  | P | Hydrophilic | Neutral |
|  |  | Q | Hydrophilic | Neutral |
|  |  | R | Hydrophilic | Positive |
|  |  | K | Hydrophilic | Positive |
| **160** | **LRR4** | N | Hydrophilic | Neutral |
|  |  | W | Hydrophobic | Neutral |
|  |  | L | Hydrophobic | Neutral |
|  |  | S | Hydrophilic | Neutral |
|  |  | Q | Hydrophilic | Neutral |
|  |  | T | Hydrophilic | Neutral |
|  |  | R | Hydrophilic | Positive |
|  |  | V | Hydrophobic | Neutral |
|  |  | G | Hydrophilic | Neutral |
| **174** | **LRR5** | N | Hydrophilic | Neutral |
|  |  | R | Hydrophilic | Positive |
|  |  | S | Hydrophilic | Neutral |
|  |  | K | Hydrophilic | Positive |
|  |  | Y | Hydrophobic | Neutral |
|  |  | T | Hydrophilic | Neutral |
| **186** | **LRR5** | V | Hydrophobic | Neutral |
|  |  | N | Hydrophilic | Neutral |
|  |  | I | Hydrophobic | Neutral |
|  |  | K | Hydrophilic | Positive |
|  |  | C | Hydrophilic | Neutral |
|  |  | G | Hydrophilic | Neutral |
|  |  | H | Hydrophilic | Positive |
| **191** | **LRR5** | N | Hydrophilic | Neutral |
|  |  | E | Hydrophilic | Negative |
|  |  | D | Hydrophilic | Negative |
|  |  | K | Hydrophilic | Positive |
|  |  | S | Hydrophilic | Neutral |
|  |  | I | Hydrophobic | Neutral |
|  |  | V | Hydrophobic | Neutral |
|  |  | A | Hydrophobic | Neutral |
| **214** | **LRR6** | S | Hydrophilic | Neutral |
|  |  | V | Hydrophobic | Neutral |
|  |  | I | Hydrophobic | Neutral |
|  |  | F | Hydrophobic | Neutral |
|  |  | H | Hydrophilic | Positive |
|  |  | Y | Hydrophobic | Neutral |
| **236** | **LRR7** | Y | Hydrophobic | Neutral |
|  |  | I | Hydrophobic | Neutral |
|  |  | A | Hydrophobic | Neutral |
|  |  | N | Hydrophilic | Neutral |
|  |  | R | Hydrophilic | Positive |
|  |  | T | Hydrophilic | Neutral |
|  |  | K | Hydrophilic | Positive |
| **249** | **LRR8** | T | Hydrophilic | Neutral |
|  |  | I | Hydrophobic | Neutral |
|  |  | R | Hydrophilic | Positive |
|  |  | S | Hydrophilic | Neutral |
|  |  | K | Hydrophilic | Positive |
|  |  | E | Hydrophilic | Negative |
| **338** | **LRR10** | R | Hydrophilic | Positive |
|  |  | V | Hydrophobic | Neutral |
|  |  | N | Hydrophilic | Neutral |
|  |  | S | Hydrophilic | Neutral |
|  |  | H | Hydrophilic | Positive |
| **349** | **LRR11** | I | Hydrophobic | Neutral |
|  |  | V | Hydrophobic | Neutral |
|  |  | Q | Hydrophilic | Neutral |
|  |  | E | Hydrophilic | Negative |
|  |  | L | Hydrophobic | Neutral |
| **388** | **LRR12** | Q | Hydrophilic | Neutral |
|  |  | E | Hydrophilic | Negative |
|  |  | K | Hydrophilic | Positive |
|  |  | R | Hydrophilic | Positive |
| **416** | **LRR13** | N | Hydrophilic | Neutral |
|  |  | D | Hydrophilic | Negative |
|  |  | W | Hydrophobic | Neutral |
|  |  | E | Hydrophilic | Negative |
|  |  | H | Hydrophilic | Positive |
|  |  | S | Hydrophilic | Neutral |
| **418** | **LRR13** | S | Hydrophilic | Neutral |
|  |  | P | Hydrophilic | Neutral |
|  |  | R | Hydrophilic | Positive |
| **441** | **Undefined region (LRR14-LRR15)** | Y | Hydrophobic | Neutral |
|  |  | E | Hydrophilic | Negative |
|  |  | S | Hydrophilic | Neutral |
|  |  | D | Hydrophilic | Negative |
|  |  | F | Hydrophobic | Neutral |
|  |  | N | Hydrophilic | Neutral |
| **459** | **Undefined region (LRR14-LRR15)** | F | Hydrophobic | Neutral |
|  |  | M | Hydrophobic | Neutral |
|  |  | S | Hydrophilic | Neutral |
|  |  | D | Hydrophilic | Negative |
|  |  | I | Hydrophobic | Neutral |
|  |  | P | Hydrophilic | Neutral |
|  |  | G | Hydrophilic | Neutral |
|  |  | Y | Hydrophobic | Neutral |
|  |  | E | Hydrophilic | Negative |
|  |  | T | Hydrophilic | Neutral |
| **472** | **Undefined region (LRR14-LRR15)** | R | Hydrophilic | Positive |
|  |  | K | Hydrophilic | Positive |
|  |  | E | Hydrophilic | Negative |
|  |  | H | Hydrophilic | Positive |
|  |  | N | Hydrophilic | Neutral |
|  |  | Y | Hydrophobic | Neutral |
|  |  | S | Hydrophilic | Neutral |
| **481** | **Undefined region (LRR14-LRR15)** | A | Hydrophobic | Neutral |
|  |  | T | Hydrophilic | Neutral |
|  |  | R | Hydrophilic | Positive |
|  |  | V | Hydrophobic | Neutral |
|  |  | N | Hydrophilic | Neutral |
|  |  | D | Hydrophilic | Negative |
|  |  | S | Hydrophilic | Neutral |
| **498** | **LRR15** | P | Hydrophilic | Neutral |
|  |  | K | Hydrophilic | Positive |
|  |  | V | Hydrophobic | Neutral |
|  |  | Q | Hydrophilic | Neutral |
|  |  | R | Hydrophilic | Positive |
|  |  | I | Hydrophobic | Neutral |
|  |  | S | Hydrophilic | Neutral |
|  |  | A | Hydrophobic | Neutral |
| **606** | **LRR19** | E | Hydrophilic | Negative |
|  |  | K | Hydrophilic | Positive |
|  |  | N | Hydrophilic | Neutral |
|  |  | Q | Hydrophilic | Neutral |
|  |  | R | Hydrophilic | Positive |
|  |  | S | Hydrophilic | Neutral |
| **629** | **LRR20** | N | Hydrophilic | Neutral |
|  |  | D | Hydrophilic | Negative |
|  |  | T | Hydrophilic | Neutral |
|  |  | R | Hydrophilic | Positive |
|  |  | G | Hydrophilic | Neutral |
|  |  | V | Hydrophobic | Neutral |
| **633** | **LRR20** | S | Hydrophilic | Neutral |
|  |  | Q | Hydrophilic | Neutral |
|  |  | K | Hydrophilic | Positive |
|  |  | P | Hydrophilic | Neutral |
|  |  | E | Hydrophilic | Negative |
| **639** | **LRR20** | K | Hydrophilic | Positive |
|  |  | R | Hydrophilic | Positive |
|  |  | S | Hydrophilic | Neutral |
|  |  | Q | Hydrophilic | Neutral |
|  |  | T | Hydrophilic | Neutral |
|  |  | E | Hydrophilic | Negative |
| **712** | **LRR23** | S | Hydrophilic | Neutral |
|  |  | H | Hydrophilic | Positive |
|  |  | T | Hydrophilic | Neutral |
|  |  | P | Hydrophilic | Neutral |
|  |  | N | Hydrophilic | Neutral |
| **761** | **LRR25** | T | Hydrophilic | Neutral |
|  |  | N | Hydrophilic | Neutral |
|  |  | A | Hydrophobic | Neutral |
|  |  | K | Hydrophilic | Positive |
|  |  | S | Hydrophilic | Neutral |
|  |  | I | Hydrophobic | Neutral |
| **766** | **LRR26** | M | Hydrophobic | Neutral |
|  |  | K | Hydrophilic | Positive |
|  |  | I | Hydrophobic | Neutral |
|  |  | V | Hydrophobic | Neutral |
|  |  | S | Hydrophilic | Neutral |
|  |  | L | Hydrophobic | Neutral |
